# Five years of national airborne pollen monitoring in South Africa: biome-specific calendars to inform allergy diagnosis and prevention

**DOI:** 10.1007/s10453-026-09909-w

**Published:** 2026-04-15

**Authors:** Takudzwa Matuvhunye, Dilys M. Berman, Nanike Esterhuizen, Andriantsilavo H. I. Razafimanantsoa, Frank H. Neumann, Dorra Gharbi, Keneilwe Podile, Tshiamo Mmatladi, Boitumelo Langa, Moteng E. Moseri, Linus Ajikah, Angela Effiom, Nikiwe Ndlovu, Lynne J. Quick, Erin Hilmer, Marishka Guscott, Shabeer Davids, Andri C. Van Aardt, J. C. Linde de Jager, Jubilant V. Sithole, Juanette John, Rebecca M. Garland, Trevor Hill, Jemma Finch, Kama Chetty, Werner Hoek, Marion Bamford, Riaz Y. Seedat, Ahmed I. Manjra, Caryn M. Upton, Jonny Peter

**Affiliations:** 1 Division of Allergology and Clinical Immunology, Department of Medicine, Faculty of Health Sciences, University of Cape Town, Cape Town, South Africa; 2 Allergy and Immunology Unit, University of Cape Town Lung Institute, Cape Town, South Africa; 3 Hortgro Science, Stellenbosch, South Africa & Department of Conservation Ecology and Entomology, Faculty of AgriSciences, Stellenbosch University, Stellenbosch, South Africa; 4 Department of Geological Sciences, Human Evolution Research Institute, University of Cape Town, Cape Town, South Africa; 5 Unit for Environmental Sciences and Management, Faculty of Natural and Agricultural Sciences, North West University, Potchefstroom, South Africa; 6 Analyis and Experimentation on Ecosystem (AnaEE Eric), CNRS Campus, Paris, France; 7 Evolutionary Studies Institute, University of the Witwatersrand, Johannesburg, South Africa; 8 Department of Plant and Ecological Studies, Faculty of Biological Sciences, University of Calabar, Calabar, Nigeria; 9 Nelson R. Mandela School of Medicine, College of Health Sciences, University of KwaZulu-Natal, Durban, South Africa; 10 African Centre for Coastal Palaeoscience, Nelson Mandela University, Gqeberha, South Africa; 11 Department of Botany and Plant Biotechnology, University of Johannesburg, Johannesburg, South Africa; 12 Department of Plant Sciences, Faculty of Natural and Agricultural Sciences, University of the Free State, Bloemfontein, South Africa; 13 Smart Places, CSIR, Pretoria, South Africa; 14 Department of Geography, Geoinformatics and Meteorology, University of Pretoria, Pretoria, South Africa; 15 Discipline of Geography, University of KwaZulu-Natal, Pietermaritzburg, South Africa; 16 Grasslands, Forests, Wetlands Node, South African Environmental Observation Network (SAEON), Pietermaritzburg, South Africa; 17 South African Weather Service, Centurion Central, Pretoria, South Africa; 18 Department of Otorhinolaryngology, Gariep Mediclinic, Kimberley, South Africa; 19 Department of Otorhinolaryngology, Faculty of Health Sciences, University of the Free State, Bloemfontein, South Africa; 20 Hiway Medical Centre, Westville Hospital, Durban, South Africa

**Keywords:** Airborne pollen, Pollen season, Pollen allergy, Southern Hemisphere

## Abstract

Pollen monitoring is crucial for understanding seasonal patterns, supporting allergy diagnosis and informing early warning tools to mitigate allergic diseases. The Southern Hemisphere lacks long-term pollen seasonal data, with extremely few records from Africa. We present pollen calendars based on five years of data from South Africa. Airborne pollen from 2019 to 2024 across South African biomes was collected in seven cities using Hirst-type volumetric spore traps and standard protocols. Daily concentrations were analysed by light microscopy. The five-year mean annual pollen integral (APIn) were calculated for each site. The five-year mean APIn was highest in the Grassland Biome (Bloemfontein) and lowest in the Albany Thicket Biome (Gqeberha). The Grassland Biome (Johannesburg) had the highest average tree pollen integral, while the Savanna Biome (Kimberley) had the highest average annual grass pollen integral. The grass family (Poaceae*)* was the most common pollen type across all biomes. Other common taxa were exotic trees Cupressaceae, *Platanus*, *Morus* and *Betula*. Tree seasons were July to September, while grass and weed pollen seasons varied across the biomes. These five-year pollen calendars provide the first biome-specific national reference for airborne pollen exposure in South Africa. The findings provide baseline data for the clinical management of allergic disease.

**Supplementary Information:**

The online version contains supplementary material available at 10.1007/s10453-026-09909-w.

## Introduction

Allergic diseases affect approximately 662 million people worldwide, including 400 million with allergic rhinitis (AR) (Lu et al., 2024). The incidence of allergic diseases has increased in recent years possibly due to anthropogenic climate change (Pershad et al., 2025), with the prevalence of AR and asthma among adolescents and university students in South Africa estimated at 40% and 10%, respectively (Zar et al., 2007; Seedat et al., 2018; Mphahlele et al., 2023). Pollen is a dominant aeroallergen driving allergic respiratory diseases. Increased pollen concentrations, changing seasonality and species assemblages have been associated with increased sensitisation, symptomatic AR and asthma across different populations (Kitinoja et al., 2020; Anuradha et al., 2006; Seedat et al., 2010; Jain et al., 2022). Negative interactions between air pollutants and pollen allergens (Li et al., 2020; Sedghy et al., 2018), together with increasing periods of extreme risk such as thunderstorm asthma events (Davies et al., 2018), highlight the importance of detailed geospatial and temporal information on pollen emissions (Ortega-Rosas et al., 2023).

In Australia, pollen concentrations peak in spring (September to December), with sensitisation of 40–46% is predominantly attributed to grasses (Davies et al., 2022; Kam et al., 2016; Lampugnani et al., 2024). In Europe and North America, spring pollen peaks, primarily from trees (birch, cypress, oak), are associated with an increased risk of AR and asthma (Seth and Bielory et al., 2021; D’Amato et al., 2023). Grass pollen peaks are in late spring and early summer, whereas autumn peaks are predominantly driven by weed (ragweed) pollen (Lappe et al., 2023). In Africa, limited records of airborne pollen are available from selected countries, including Nigeria (Adeniyi et al., 2018; Agwu et al., 2005) (Necib & Boughediri, 2016) and Morocco (EIhassani et al., 2022; Raissouni et al., 2024). Pollen from exotic trees such as *Platanus* and *Quercus* is abundant and has been reported as a major allergen among adults in the Northwest Province of South Africa (Gharbi et al., 2025).

Airborne pollen studies in Africa have been limited to a few cities, using different methods ranging from the gravity method using Vaseline-coated slides (Coetzee & van Zinderen Bakker, 1952) to specialised equipment such as the Lanzoni Seven-Day Recording Volumetric Spore Trap (Neumann et al., 2025), which has made direct comparisons difficult. Pollen seasons are influenced by factors such as rainfall, temperature, humidity, wind speed and direction (Dahl et al., 2013) and vary by geographic location (Camacho et al., 2020), showing the importance of establishing biome-specific pollen calendars. This underscores the importance of identifying seasonal trends and specific pollen taxa using longer-term datasets across diverse biomes.

Note that, for aerobiological standard terminology, we followed Galan et al. (2017). A pollen calendar visually summarises longitudinal trends in the timing, duration, and intensity of allergenic pollen seasons throughout the year for key plant taxa, thereby supporting the diagnosis and management of allergic diseases (Galan et al., 2017; Lo et al., 2019). Pollen calendars guide allergy sufferers in taking precautions or early preventive medication and assist clinicians in identifying potential triggers and initiating appropriate therapies (Ortega-Rosas et al., 2023). Long-term pollen calendars exist predominantly in the Northern Hemisphere (Camacho et al., 2020; Cervigón et al., 2024). The South African Pollen Monitoring Network (SAPNET), initiated for national pollen monitoring in 2019 (Ajikah et al., 2020), has published pollen calendars for seven cities representing the diverse biomes of South Africa, based on two years of airborne monitoring (Esterhuizen et al., 2023). Here, we present the first biome-specific five-year national pollen calendars for South Africa, extending the previous report and demonstrating substantial spatial variability in pollen intensity and seasonality.

## Material and methods

### Sites and airborne pollen sampling

**Fig. 1:**
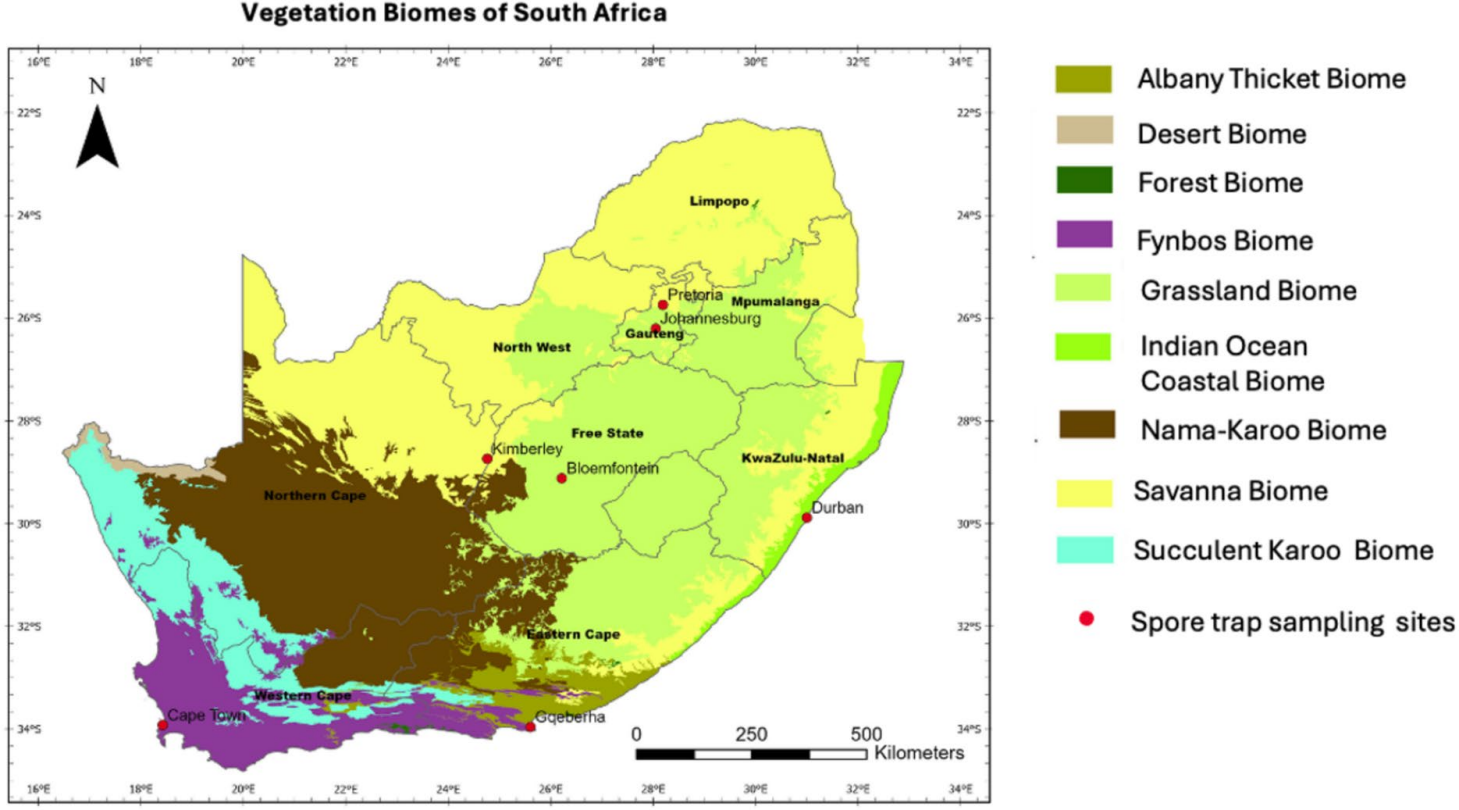
Map of South African biomes (Mucina et al., 2006) showing the seven cities where airborne pollen monitoring was conducted from 2019 to 2024

Airborne pollen was collected from August 2019 to August 2024 across seven South African cities representing different biomes, these being, the inland sites: Grassland (Johannesburg and Bloemfontein) and Savanna (Pretoria and Kimberley); and the coastal sites: Fynbos (Cape Town), Indian Ocean Coastal Belt (Durban) and Albany Thicket (Gqeberha, formerly Port Elizabeth) (Fig. 1). South Africa’s biomes and their climatic characteristics have been described previously (Esterhuizen et al., 2023) (Supplementary Table S1). The standard methods for instrument setup, and pollen collection using Hirst-type volumetric spore traps (manufactured by Burkard, UK), and light microscopy were followed (Esterhuizen et al., 2023). After seven days of sampling, the Melinex strip was stained and mounted, and analysed using a light microscope at a magnification of × 400 (Galán et al., 2014). For each slide representing a 24-h section, three longitudinal transects of the prepared slide were counted (Comtois et al., 1999; Mandrioli et al., 1998).

### Pollen metrics

Daily pollen concentrations were obtained by converting raw scores into a concentration per cubic metre of air per 24-h period and applying a correction factor to account for the Hirst-type volumetric trap flow rate of 10 L per minute (Esterhuizen et al., 2023). Weekly pollen counts were derived from daily pollen concentrations. Missing daily data were substituted by the average daily pollen count for that week. The average daily concentrations for the corresponding calendar week in other years were used to fill gaps of greater than six days (Rodríguez et al., 2021). This included data for the period 1 July 2019 to 11 August 2019, as monitoring of airborne pollen started on 12 August 2019 (Supplementary Fig. S1). The annual pollen integral (APIn) was calculated as the sum of weekly pollen concentrations for each seasonal year (52 weeks). For all analyses, we defined the seasonal year as 1 July to 30 June of the following year. For the pollen calendars, daily pollen counts were summed to weekly totals and averaged across the 5-year period to generate the average weekly profile over the year. The weekly pollen counts were grouped to determine intensity based on the following scale: 0 (0–3 pollen/m^3^), 1 (3–9 pollen/m^3^), 2 (10–29 pollen/m^3^), 3 (30–99 pollen/m^3^) and 4 (> 100 pollen/m^3^) (Esterhuizen et al., 2023). For each city, pollen types contributing at least 3% of the five-year mean APIn were included in the calendars. The start and end of the pollen season were defined as the first and last days in the seasonal year when daily grass and weeds (herbaceous shrubs) counts were above 10 pg/m^3^ (Davies et al., 2022) and above 15 pg/m^3^ for trees (Esterhuizen et al., 2023). All statistical analyses were performed using the statistical software R, version 4.5.2 (R Core Team, 2024).

## Results

### Diversity of pollen taxa

A total of 103 pollen taxa were identified across the biomes of South Africa, including 50 trees and 51 weed pollen types (Supplementary Tables S2 and S3). Weeds were the most diverse pollen type in the Indian Ocean Coastal Belt (Durban) and Albany Thicket Biome (Gqeberha), while trees were more diverse than weeds in the Grassland (Bloemfontein) and the Savanna biomes (Pretoria) (Supplementary Fig. S2). Tree pollen showed greater diversity during September and October in all biomes, except in the Grassland (Johannesburg) where it peaked in August. The grasses included *Zea mays* and other species of the Poaceae family. The pollen species of Poaceae that are common in South African biomes, based on data from the Global Biodiversity Information Facility (GBIF), are listed in the supplementary data (Supplementary Fig. S3).

### Average annual pollen integral

**Fig. 2:**
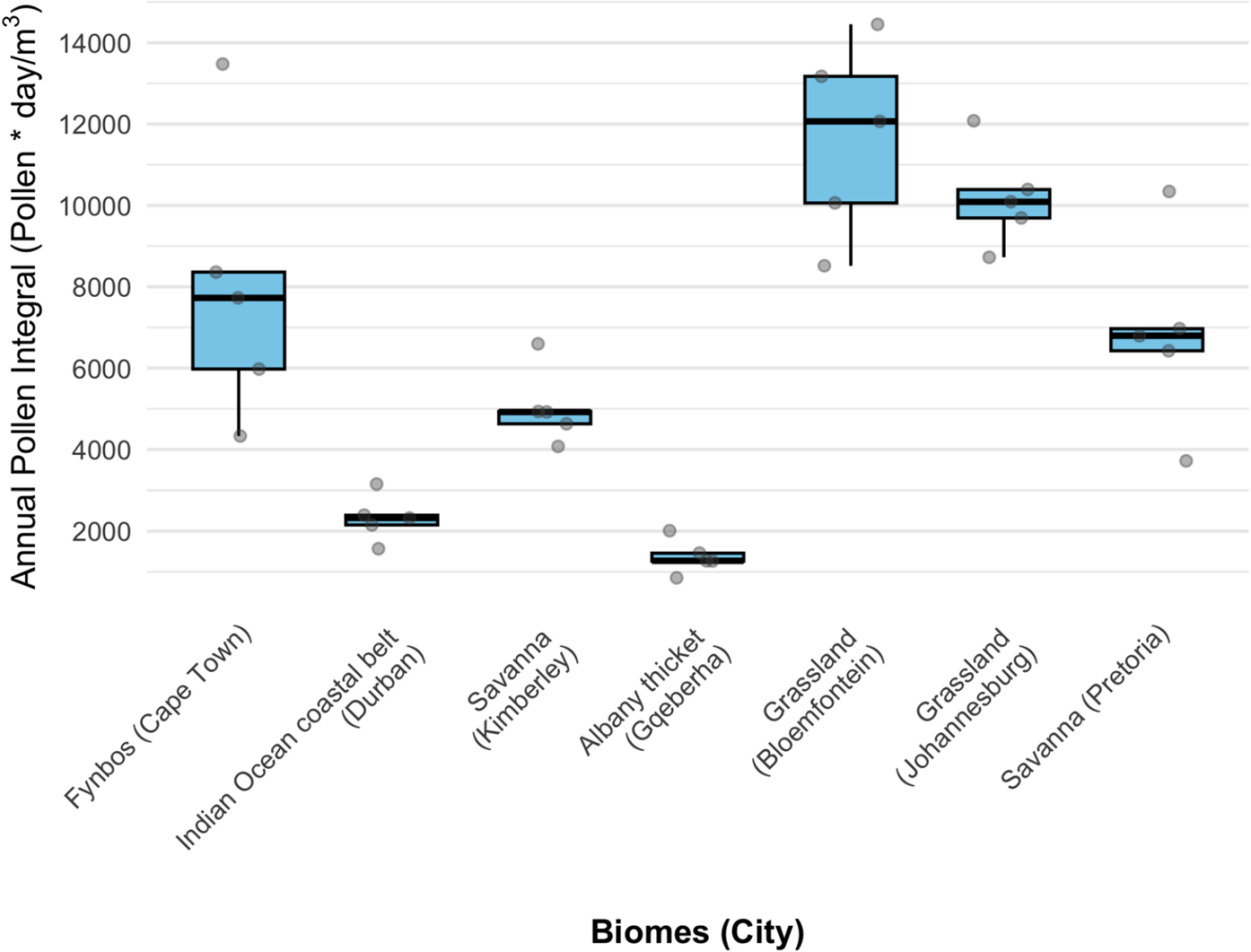
Annual pollen integral for each of the seven South African cities representing distinct biomes over five seasonal years (2019–2024). Each dot represents the yearly APIn at each city. The boxplots show the median, interquartile range (IQR) and variability of APIn values within each city

The five-year mean APIn was highest in the Grassland Biome (Bloemfontein) (Fig. 2; Table 1). The inland Savanna (Kimberley) had the highest grass pollen, followed by Bloemfontein, with substantially lower counts in the coastal biomes. Johannesburg’s Grassland had the highest APIn for trees, reflecting a strong urban forest component. High tree pollen counts were observed in Bloemfontein, Pretoria and Cape Town. APIn for weeds were lower than for trees and grasses at all sites (Supplementary Fig. S4).

**Table 1:** A summary of the annual pollen integral across the South African biomes

**Biome (City**)	**Group**	**Season year**					**Seasonal period**		**Total APIn**^**c**^	**Mean APIn**^**d**^
		**2019–2020**	**2020–2021**	**2021–2022**	**2022–2023**	**2023–2024**	**2019–2021**^a^	**2022–2024**^**b**^		
*Coastal*										
Fynbos (Cape town)	Grass	859	2591	1481	1057	915	3450	1972	6903	1381
	Trees	5389	3573	10,063	5788	2669	8962	8457	27,482	5496
	Weeds	654	874	2147	1431	777	1528	2208	5883	1177
	Total	6902	7038	13,691	8276	4361	13,940	12,637	40,268	8054
Indian Ocean coastal belt (Durban)	Grass	781	581	589	305	301	1362	606	2557	511
	Trees	857	930	1457	1247	784	1787	2031	5275	1055
	Weeds	459	666	931	531	402	1125	933	2989	598
	Total	2097	2177	2977	2083	1487	4274	3570	10,821	2164
Albany thicket (Gqeberha)	Grass	336	410	309	305	568	746	873	1928	386
	Trees	268	360	476	473	472	628	945	2049	410
	Weeds	247	502	479	681	967	749	1648	2876	575
	Total	851	1272	1264	1459	2007	2123	3466	6853	1371
*Inland*										
Grassland (Johannesburg)	Grass	1850	1782	1129	1396	1448	3632	2844	7605	1521
	Trees	6650	8803	6575	7748	8013	15,453	15,761	37,789	7558
	Weeds	1147	1423	961	902	895	2570	1797	5,328	1066
	Total	9647	12,008	8665	10,046	10,356	21,655	20,402	50,981	10,197
Savanna (Pretoria)	Grass	1848	1304	1679	1089	1334	3152	2423	7254	1451
	Trees	4214	1950	3562	8554	5006	6164	13,560	23,286	4657
	Weeds	714	456	1157	659	614	1170	1273	3600	720
	Total	6776	3710	6398	10,302	6954	10,486	17,256	34,140	6828
Grassland (Bloemfontein)	Grass	5895	3884	2803	1336	1318	9779	2654	15,236	3047
	Trees	3676	9444	4528	9582	10,334	13,120	19,916	37,564	7513
	Weeds	487	1114	1179	1139	1510	1601	2649	5388	1078
	Total	10,058	14,442	8510	12,057	13,162	24,500	25,242	58,229	11,646
Savanna (Kimberley)	Grass	3785	3515	4128	1826	2498	7300	4324	15,752	3150
	Trees	576	1010	1599	2475	1157	1586	3632	6817	1363
	Weeds	253	399	863	610	415	652	1040	2540	508
	Total	4614	4924	6590	4911	4070	9538	8996	25,109	5022

^a^Sum of daily pollen concentration in the first two season years (2019–2020 and 2020–2021). Pollen concentration is expressed per cubic metre (pollen/m^3^)

^b^Sum of daily pollen concentration in the last two season years (2022–2023 and 2023–2024)

^c^Sum of daily pollen concentration across all five years (2019–2024)

^d^Mean annual pollen integral across the five years (2019–2024)

**Fig. 3:**
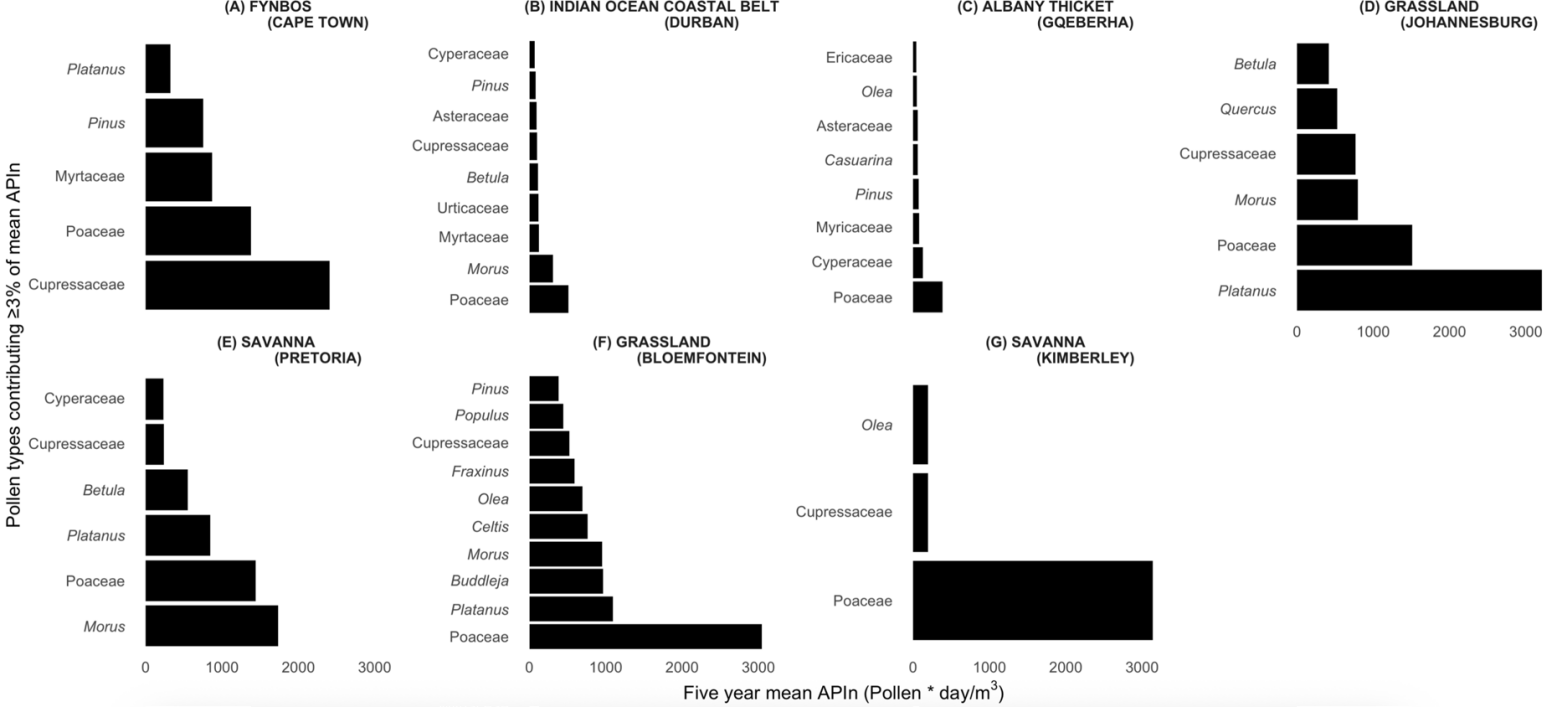
Relative contribution of pollen taxa to the annual pollen integral (APIn) across seven South African cities. Only taxa contributing more than 3% to the five-year mean APIn are shown. Bars represent the cumulative contribution of each taxon from 2019 to 2024, illustrating differences in dominant pollen types across biomes

Among the pollen types that contributed at least 3% to the city-specific five-year mean, Poaceae was detected in all biomes, while *Cupressus* was detected in all biomes, except the Albany Thicket Biome. Other common pollen types included *Platanus* (Fynbos, Savanna and Grassland sites), *Morus* (Savanna, Grassland sites and the Indian Ocean Coastal Belt) and *Betula* (Savanna, Grassland and the Indian Ocean Coastal Belt) (Fig. 3). The following taxa contributed at least 3% to the city-specific five-year mean, but only at a single site each: Urticaceae (Indian Ocean Coastal Belt), Ericaceae and *Casuarina* (Albany Thicket), *Buddleja*, *Celtis*, *Fraxinus*, *Populus* and *Quercus* (Grassland).

A comparison of the total APIn between the first two seasonal years (2019–2020 and 2020–2021) and the last two years (2022–2023 and 2023–2024) showed a drop in APIn over time across most biomes, except in Pretoria (10 486 vs. 17 256 pollen * day/m^3^), Bloemfontein (24 500 vs. 25 242 pollen * day/m^3^) and Gqeberha (2 123 vs. 3 466 pollen * day/m^3^) (Table 1). A similar pattern was observed for grass pollen, with integrals almost halving over time across all biomes, except in Gqeberha where there was a slight increase (746 vs. 873 pollen * day/m^3^). In contrast, tree pollen integrals doubled during the last two years in Pretoria (6 164 vs. 13 560 pollen * day/m^3^), Bloemfontein (13 120 vs. 19 916 pollen * day/m^3^) and Kimberley (1 586 vs. 3 632 pollen * day/m^3^). There was no clear pattern for weed pollen, although three biomes showed increases: Cape Town (1528 vs. 2208 pollen * day/m^3^), Bloemfontein (1601 vs. 2649 pollen * day/m^3^) and Kimberley (652 vs. 1040 pollen * day/m^3^). Analyses of years with high and low pollen concentrations revealed that seasonal patterns were similar and differences were mainly in the concentration of pollen (Supplementary Fig. S5). The taxa contributing ≥ 3% of the APIn were consistent across the years (Supplementary Table S4).

### Pollen calendars

**Fig. 4:**
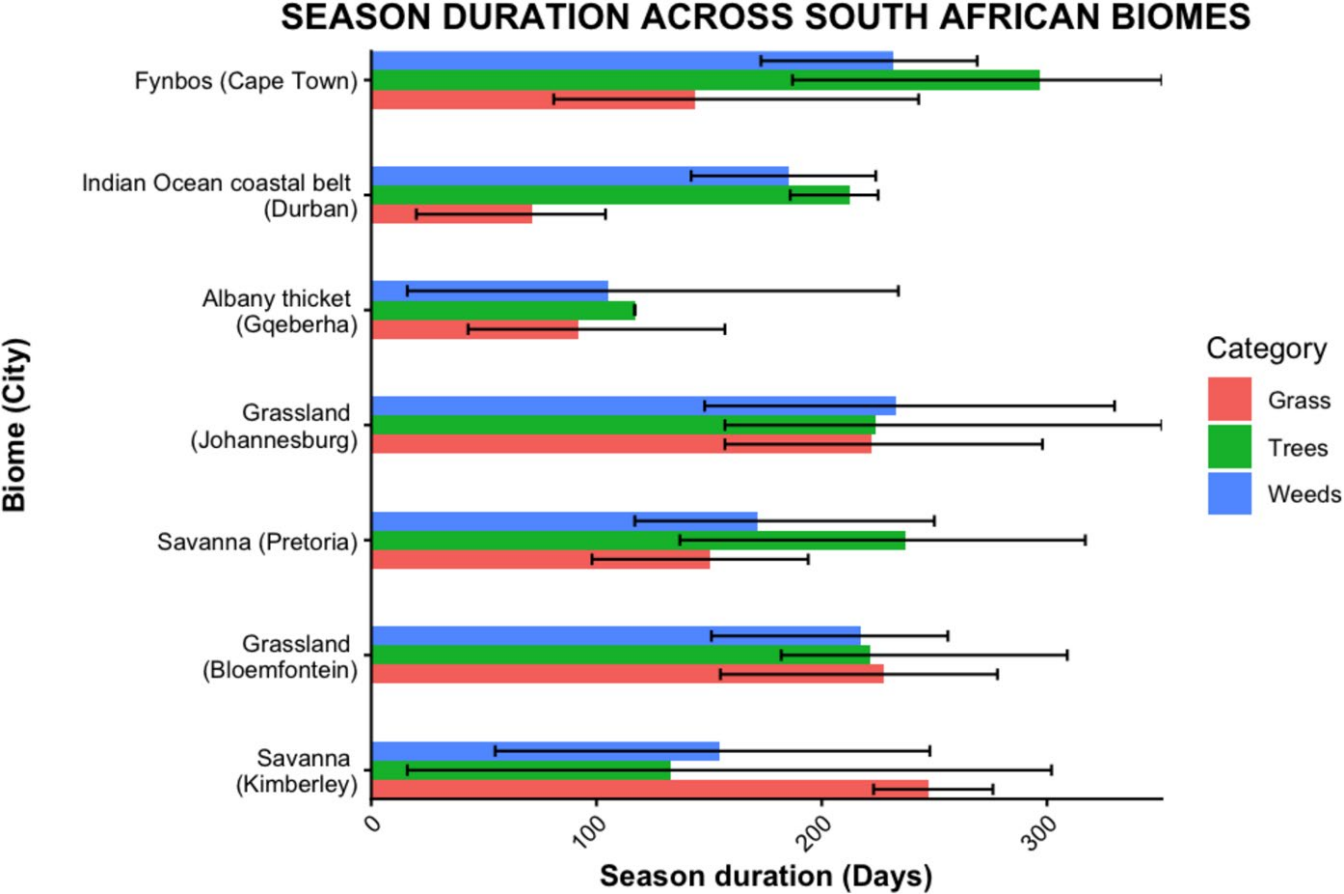
Pollen season duration for grass, trees and weeds across the seven South African cities representing distinct biomes over five seasonal years (2019–2024). The bars represent the average season duration, while the lines indicate the range

The start dates (Supplementary Fig. S6) and season durations (Fig. 4) of the pollen seasons for trees, grasses, and weeds varied between taxa and across biomes, with grass seasons being generally longer in inland regions and tree seasons longest in the Fynbos Biome. Across all the biomes, the start month of the tree pollen season was consistently between late July and September. Fynbos (Cape Town) had the longest tree pollen season (mean 297 days), while the Albany Thicket (Gqeberha) had the shortest tree pollen season (mean 116 days) (Table S3). The grass flowering season was longest in the Savanna (Kimberley; mean 247 days) and shortest in the Indian Ocean Coastal Belt Biome (Durban; mean 71 days).

### Biome characterisation

The biome pollen profiles including start, end and peak timing for all pollen groups are presented in Supplementary Table S5.

#### Fynbos Biome (Cape Town)

**Fig. 5:**
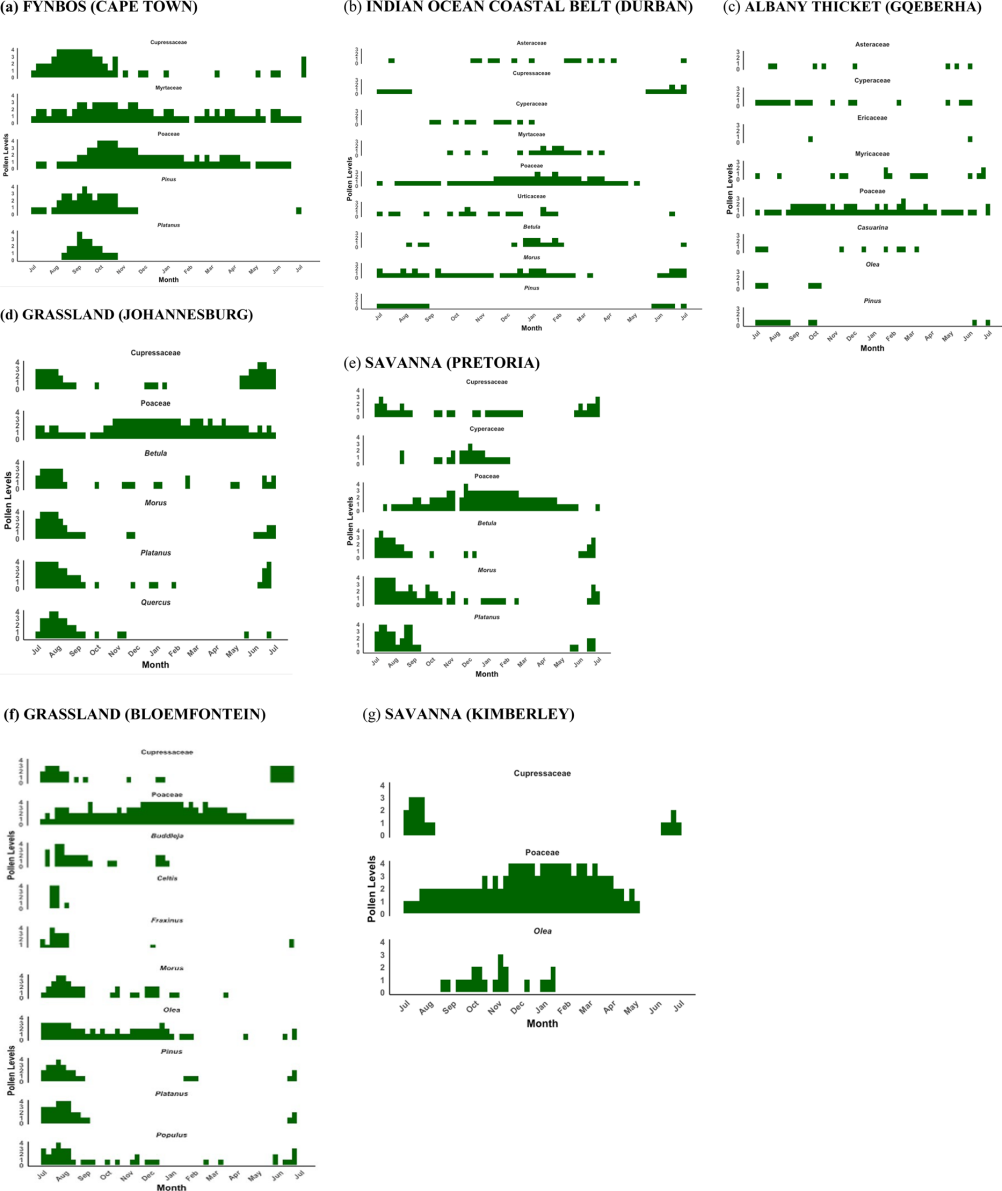
Five-year monthly pollen calendars for seven South African biomes. Stacked blocks represent mean weekly concentrations for each taxon, averaged over five seasonal years (2019–2024). Rows represent pollen taxa contributing more than 3% to the annual pollen integral at each biome, as shown in A to G. Block height increases with concentration: one block for 0–3 pollen/m^3^, two blocks for 3–10 pollen/m^3^, three blocks for 10–30 pollen/m^3^ and four blocks for concentrations above 30 pollen/m^3^. These calendars highlight differences in timing, duration and intensity of pollen seasons across biomes

**Fig. 6:**
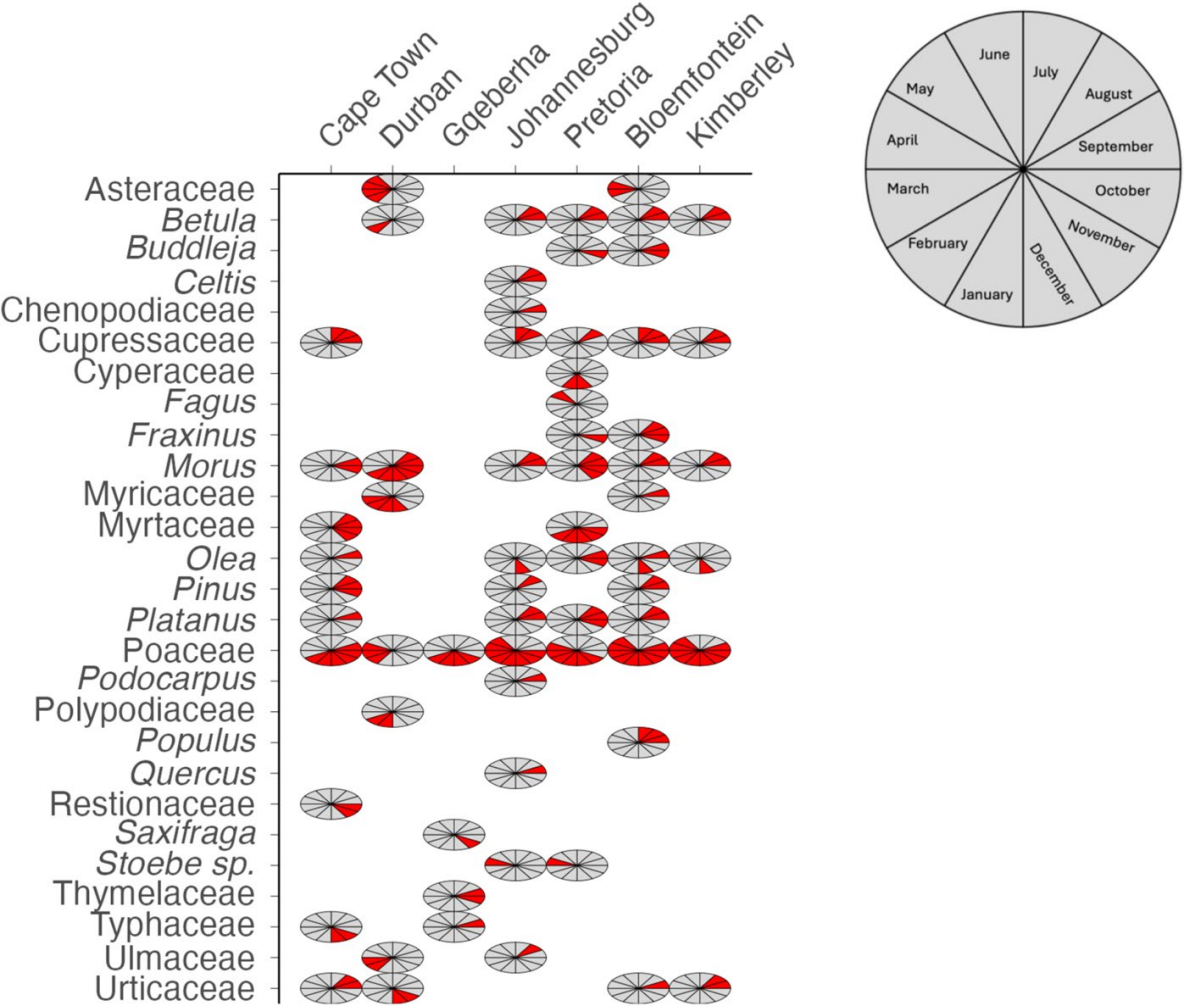
Pollen calendar for taxa with distinct pollen seasons across South African biomes. The pie charts are divided into monthly segments, starting from July, and the red shaded segments depicting the months in which pollen season concentration exceeded the seasonal daily thresholds of 15 pollen/m^3^ for trees, and 10 pollen/m^3^ for grass and weeds

The Fynbos Biome (Cape Town) showed an APIn that is high among the coastal cities but lower than the highest inland biomes (Table 1). The biome is characterised by winter rain, high plant biodiversity and a largely treeless landscape although pines and other exotic trees have been planted (Mucina et al., 2006). The grass season started in September (spring) and lasted 144 days, ending in February (summer) (Figs. 5a and 6). The Fynbos Biome featured the longest average tree season nationally (297 days), starting consistently in late winter (July). The grass pollen load showed marked interannual variability. The pollen profile was dominated by tree taxa (Table 1 and Supplementary Fig. S4). Notably, Cupressaceae accounted for the largest percentage (30%) of the five-year mean APIn. Other significant tree taxa included Myrtaceae (11%), *Pinus* (9%) and *Platanus* (4%). Poaceae contributed substantially (17%). Less common but consistently detected tree pollen types are *Olea* (2.6%) and Urticaceae (2.4%). These low-abundance taxa contribute yearly to the total pollen load.

#### Indian Ocean Coastal Belt Biome (Durban)

The Indian Ocean Coastal Belt Biome (Durban), characterized by (sub)tropical climate with high summer rainfall and abundant tree taxa including palms and mangroves (Mucina et al., 2006), had a low overall pollen catch, consistent with the pattern observed in other coastal biomes (Fig. 2, Table 1). The total pollen catch was evenly distributed across the major groups. Tree pollen was slightly higher than grass and weeds (Table 1). The grass season was the shortest of all the cities measured, lasting an average of 71 days and starting late in summer. The tree season was long, averaging 212 days, starting in winter. The weed season averaged 186 days, beginning in spring (Fig. 5b). Poaceae contributed 21.9% to the mean APIn, while the exotic tree *Morus*, contributed 13.2%. Other significant trees included Myrtaceae (5.4%) and *Pinus* (3.4%).

#### The Albany Thicket Biome (Gqeberha, formerly Port Elizabeth)

The Albany Thicket Biome (Gqeberha) features dense evergreen, sclerophyllous or succulent shrubland with high biodiversity under predominantly all-year rainfall conditions (Mucina et al., 2006). The lowest five-year mean APIn was observed among all seven cities, and this biome featured the shortest pollen seasons nationally for trees and grasses. The tree and grass pollen loads were lower than in the other biomes, and their seasons were shorter, lasting an average of 116 days and 92 days, respectively. Weed seasons were short, lasting an average of 105 days, and showed low-to-moderate levels of pollen throughout, resulting in no clear seasons (Fig. 5c). Poaceae was the leading contributor, accounting for 28% of the mean APIn. Other prominent taxa contributing more than 3% of the APIn yearly included Cyperaceae (9%), Myricaceae (5%) and *Pinus* (5%).

#### Grassland Biome (Johannesburg)

The Grassland had the highest averaged tree pollen concentration among all monitored sites, mainly contributed by exotic trees. The biome is situated in the summer-rainfall region, largely treeless and often highly transformed due to agriculture and urbanisation, including the introduction of abundant exotic trees (Mucina et al., 2006). The high five-year mean APIn included a high contribution of tree and grass pollen (Table 1). The pollen season was characterised by a similarly long period of high tree concentration that lasted an average of 224 days, starting in mid-winter (Table S3). The grass season was similarly long, lasting an average of 222 days. Weed seasons had the longest average duration nationally at 233 days, beginning in early spring. The pollen profile was highly diverse and dominated by several key tree taxa (Fig. 5d). The leading contributor was the alien tree *Platanus*, accounting for the largest percentage of the mean APIn (31%). Other highly relevant tree taxa included *Morus* (7.8%), Cupressaceae (7.6%), *Quercus* (5.2%) and *Betula* (4.1%). Poaceae also contributed substantially to the overall pollen load (14.8%). Other pollen types consistently detected every year included Asteraceae, *Fraxinus* and *Celtis* (Supplementary Table 4).

#### Savanna Biome (Pretoria)

Savanna (Pretoria) pollen spectra recorded a high overall APIn (Fig. 2, Table 1). The Savanna Biome is a grass-dominated ecosystem with subtropical trees such as acacias (Mucina et al., 2006). The largest contributor to the APIn was tree pollen, with grass and weed pollen contributing a smaller but substantial portion (Table 1, Supplementary Fig. 4). This biome featured a long pollen seasons, where the tree season averaged 237 days, starting early in spring. The grass season was shorter, averaging 150 days, while the weed season averaged 172 days, both starting in spring. *Morus* (25.3%) dominated the tree pollen catch. Poaceae was the second-highest contributor (21%). Other significant tree taxa included *Platanus* (12%), *Betula* (8%), Cupressaceae (3.5%) and Cyperaceae (3%) (Supplementary Table S2).

#### Grassland (Bloemfontein)

The temperate Grassland Biome, where Bloemfontein is situated, recorded the highest five-year mean APIn in the study, due to a combination of high tree and grass pollen concentrations. The overall pollen load was dominated by the combined contribution of tree and grass pollen (Table 1, Supplementary Fig. S4). Long pollen seasons were consistently observed for all major groups. The grass flowering season was the longest, averaging 246 days and starting in early spring (Supplementary Figs. S7 and S8). The tree season was also long, averaging 221 days and beginning in winter. The weed season averaged 217 days, starting in spring. Unlike most other biomes, Poaceae was the only pollen type that consistently contributed more than 3% of the APIn throughout the five years, accounting for 26% of the mean APIn. Other pollen types that were common every year, though contributing slightly below the 3% threshold, included Cyperaceae (2.7%) and Asteraceae (2.4%).

#### Savanna Biome (Kimberley)

Savanna (Kimberley), with low rainfall and an open, grassy landscape (Mucina et al., 2006), recorded the highest averaged grass pollen concentration among all seven monitored sites. The overall five-year mean APIn was characterized by a distinct grass dominance, with grass pollen contributing the most to the total load (Table 1). The grass season was notably long, averaging 247 days, and starting relatively early in spring. The tree season was shorter, lasting an average of 163 days, and starting in winter. The weed season was intermediate, averaging 155 days. The pollen profile was dominated by grasses (Poaceae). In terms of tree taxa, *Ole*a and Cupressaceae were the only types that contributed more than 3% of the APIn yearly. Other consistent contributors that were above 2% included *Morus* (2.9%) and *Betula*.

## Discussion

The African continent’s aerobiology is in early stages of development, with a paucity of long-term pollen monitoring. We provide national biome-resolved pollen calendars for South Africa, based on five years of standardised airborne pollen monitoring across seven cities. These calendars extend the earlier two-year report from SAPNET (Esterhuizen et al., 2023) and establish the first long-term evidence base for understanding national airborne allergen exposure.

Our data provide the critical baseline for understanding temporal and spatial patterns of airborne pollen across the diverse biomes in South Africa. The pollen calendars support comparative studies across Africa and offer the first steps towards developing predictive models for pollen trends in understudied regions. Our results offer a practical reference to support area-specific clinical practice, anticipate exacerbation risks, plan public health measures and develop early warning systems for allergic diseases.

Inland biomes, particularly the Grassland (Bloemfontein) and Savanna (Kimberley), had markedly higher annual pollen loads and longer seasons relative to coastal biomes. Potential reasons are complex and might encompass higher rainfall rates at the coast, which wash out pollen from the atmosphere, and stronger temperature fluctuations including urban heat island effects in more continental, interior cities (Schramm et al., 2021). Coastal biomes, where Cape Town, Durban and Gqeberha are situated, had shorter, concentrated seasons reflecting differences in vegetation structure, land use, humidity and wind patterns (Esterhuizen et al., 2023; González Minero et al., 1993). Inland grass seasons were greater than 200 days, whereas Durban and Gqeberha had short seasons of less than 100 days. Similar patterns have been reported in Australia and southern Europe, where Mediterranean and subtropical climates are associated with shorter, more intense pollen seasons compared with temperate continental climates which sustain extended flowering, and longer seasons (Davies et al., 2022; Frenguelli et al., 2014; Montiel et al., 2025).

Tree pollen seasons were consistent across all biomes, starting in late winter, a pattern reported in Europe and North America (Monroy-Colín et al., 2020; Paschalidou et al., 2020). Cape Town had an exceptionally long tree season lasting nearly 300 days, which may blur the distinction between seasonal and perennial allergens. The need for biome-specific pollen calendars rather than referencing calendars from Europe or North America is a result of complex climate zones in South Africa. Here we have shown that risk profiles differ substantially by biome and require region-specific interpretation. The dominant allergenic taxa varied by biome. Grasses (Poaceae) were detected in all cities and formed the primary aeroallergen, consistent with global patterns (Camacho et al., 2020; Medek et al., 2016; Ortega-Rosas et al., 2023). Poaceae includes over 12 000 wind-pollinated species and is a major allergen source in Europe (Frisk et al., 2023; García-Mozo, 2017).

Tree pollen contributed disproportionately to the pollen spectra of Johannesburg and Pretoria, where the urban forest is dominated by allergenic alien tree species such as *Platanus*, Cupressaceae, *Quercus*, *Betula, Pinus,* and Myrtaceae (mostly pollen of Australian exotic *Eucalyptus*) (Caillaud et al., 2014; Huang et al., 2024). Birch (*Betula*), although alien to South Africa, was detected in all biomes and is a major cause of allergic rhinitis in the Northern Hemisphere (Biedermann et al., 2019; D’Amato et al., 1998), highlighting exposure not reflected in typical local allergen panels (Murray et al., 2022; Pedretti et al., 2025). Weed pollen was the least abundant group but showed taxonomic diversity in coastal areas, where fynbos-like erica and protea species were regularly identified from sampling. We observed a decline in pollen concentrations between the first two and the last two years. This anomaly requires further investigation together with meteorological parameters and land use changes that may have affected pollen concentrations.

Strengths of this study include the use of standardised Hirst-based methods across multiple biomes, enabling quantitative comparison between cities, and the generation of clinically accessible weekly pollen calendars. However, a limitation is coverage, with pollen monitoring restricted to only seven urban centres, leaving many provinces and rural areas without direct coverage, although other cities have been mapped previously, as reported (Cadman et al., 1994; Gharbi et al., 2024). The calendars therefore provide a national reference framework of substantial data but may not capture local exposure in underrepresented cities. Another limitation is the inability of light microscopy to determine Poaceae beyond the family level, despite its importance as the dominant allergenic group nationally. There is no consensus on defining the start or end of the pollen season; we therefore used a threshold-based method for consistency with our previous work (Esterhuizen et al., 2023). Other methods for determining the start or end of pollen season are based on percentages (such as 5% / 95% of the APIn), quantiles and moving thresholds (Anderegg et al., 2021; Glick et al., 2021; Zhang et al., 2015). Furthermore, our pollen calendars were constructed using weekly summed pollen counts averaged across the study period following Esterhuizen et al. (2023). Advanced methods such as the progressive pollen calendar methods that account for interannual variability and extreme events (e.g. Gehrig et al., 2018; Scevkova et al., 2025) were not applied in this study, as they require longer datasets.

These findings have significant implications for healthcare and public health. Pollen calendars can guide diagnostic testing, inform treatment plans and advice patients on when to initiate or escalate preventive therapy. For public health agencies, the data provide a foundation for the development of an early warning system that integrates pollen, air quality and weather information to anticipate surges in allergic rhinitis and asthma. Maintaining and expanding pollen monitoring in South Africa is therefore a core component of environmental health surveillance. Without continuous data, changing exposure cannot be tracked, and early warnings cannot be implemented or scaled. Strengthening the network through expanded geographic coverage, incorporation of molecular identification such as DNA metabarcoding for Poaceae (Van Haeften et al., 2024) and fungal spores (Banchi et al., 2020), and integration into national health information systems will be critical for supporting allergy care, reducing respiratory morbidity and mortality and preparing for the impacts of climate variability. The calendars presented here provide both a baseline and a compelling rationale for sustaining and enhancing pollen monitoring as part of South Africa’s broader climate and health response.

## Conclusion

In this study, we have provided the first five-year, biome-specific pollen calendars for South Africa. Our biomes show substantial variation in pollen exposure between regions, taxa and years. Poaceae and alien tree taxa such as *Platanus*, Cupressaceae, *Betula* and Myrtaceae are the dominant aeroallergens. These findings establish a national reference for pollen exposure patterns and underscore the need for ongoing monitoring. Expansion to map unexplored areas and to understand local variability and the impact of alien taxa is necessary.

## Supplementary Information

Below is the link to the electronic supplementary material.

Supplementary file1 (DOCX 7013 KB)

## Data Availability

Additional results are in the Supplementary. All datasets used or analysed during the current study are available from the corresponding author upon reasonable request.
