# Supplementary material for: Five years of national airborne pollen monitoring in South Africa: biome-specific calendars to inform allergy diagnosis and prevention
Source: Aerobiologia (Bologna). 2026 Apr 15;42(1):23. doi: 10.1007/s10453-026-09909-w (PMC13083431; doi:10.1007/s10453-026-09909-w)
Supplement: Supplementary file 1 — Supplementary file1 (DOCX 7013 KB) [file 10453_2026_9909_MOESM1_ESM.docx]

Five years of national airborne pollen monitoring in South Africa: Biome- specific calendars to inform allergy diagnosis and prevention.

Takudzwa Matuvhunye^1,2^, Dilys M. Berman^1,2^, Nanike Esterhuizen^1,2,3^, Andriantsilavo H. I. Razafimanantsoa^2,4^, Frank H. Neumann^5^, Dorra Gharbi^5,6^, Keneilwe Podile^5^, Tshiamo Mmatladi^5^, Boitumelo Langa^5^, Moteng E. Moseri^5^, Linus Ajikah^7,8^, Angela Effiom^7^, Nikiwe Ndlovu^5,9^, Lynne J. Quick^10^, Erin Hilmer^10^, Marishka Guscott^10^, Shabeer Davids^10,11^, Andri C. Van Aardt^12^, J.C Linde de Jager^12^, Jubilant V. Sithole^12^, Juanette John^13^, Rebecca M. Garland^13,14^, Trevor Hill^15^, Jemma Finch^15,16^, Kama Chetty^17^, Werner Hoek^18^, Marion Bamford^7^, Riaz Y. Seedat^19^, Ahmed I. Manjra^20^, Caryn M Upton^1,2^ and Jonny Peter^1,2^, on behalf of the SAPNET consortium.

1. Division of Allergology and Clinical Immunology, Department of Medicine, University of Cape Town, Cape Town, South Africa.
2. Allergy and Immunology Unit, University of Cape Town Lung Institute, Cape Town, South Africa
3. Hortgro Science, Stellenbosch, South Africa & Department of Conservation Ecology and Entomology, Faculty of AgriSciences, Stellenbosch University, Stellenbosch, South Africa.
4. Human Evolution Research Institute, Department of Geological Sciences, University of Cape Town, Cape Town, South Africa
5. Unit for Environmental Sciences and Management, Faculty of Natural and Agricultural Sciences, North West University, Potchefstroom, South Africa
6. Analyis and Experimentation on Ecosystem (AnaEE Eric), CNRS Campus, Paris, France
7. Evolutionary Studies Institute , University of the Witwatersrand, Johannesburg, South Africa
8. Department of Plant and Ecological Studies, Faculty of Biological Sciences, University of Calabar, Calabar, Nigeria
9. Nelson R. Mandela School of Medicine, College of Health Sciences, University of KwaZulu-Natal, Durban, South Africa.
10. African Centre for Coastal Palaeoscience, Nelson Mandela University, Gqeberha, South Africa
11. Department of Botany and Plant Biotechnology, University of Johannesburg, Johannesburg, South Africa
12. Department of Plant Sciences, Faculty of Natural and Agricultural Sciences, University of the Free State, Bloemfontein, South Africa
13. Smart Places, CSIR, Pretoria, South Africa
14. Department of Geography, Geoinformatics and Meteorology, University of Pretoria, Pretoria, South Africa
15. Discipline of Geography, University of KwaZulu-Natal, Pietermaritzburg, South Africa
16. South African Environmental Observation Network (SAEON), Grasslands, Forests, Wetlands Node, Montrose, 3201, South Africa.
17. South African Weather Service**,** Centurion Central**,** South Africa.
18. Department of Otorhinolaryngology, Gariep Mediclinic, Kimberley, South Africa
19. Department of Otorhinolaryngology, Faculty of Health Sciences, University of the Free State, Bloemfontein, South Africa
20. Hiway Medical Centre, Westville Hospital, Durban, South Africa

**Correspondence**

Jonny Peter, H52 Old Main Building, Groote

Schuur Hospital, Observatory, Cape Town,

South Africa.

Email: [Jonny.Peter@uct.ac.za](mailto:Jonny.Peter@uct.ac.za)

**SUPPLEMENTARY FIGURES**

**Fig. S1** Daily pollen concentrations from 2019 to 2024 for the seven biomes (A to G) across South Africa. For each biome, the original data with gaps is shown in (i), and the data after gaps were filled using weekly averages is shown in (ii).

(a) **FYNBOS BIOME (CAPE TOWN)**

| (i)  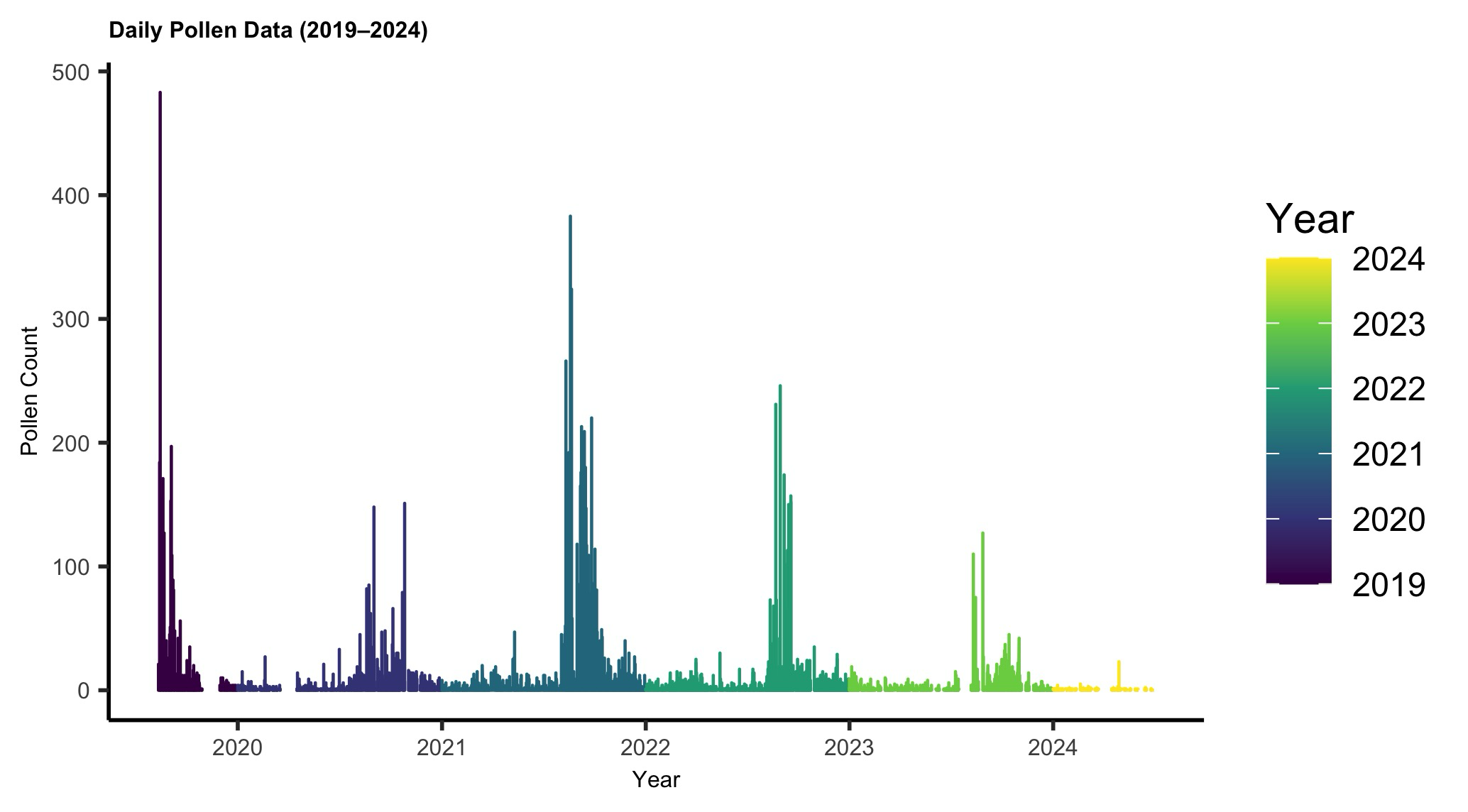  (ii)  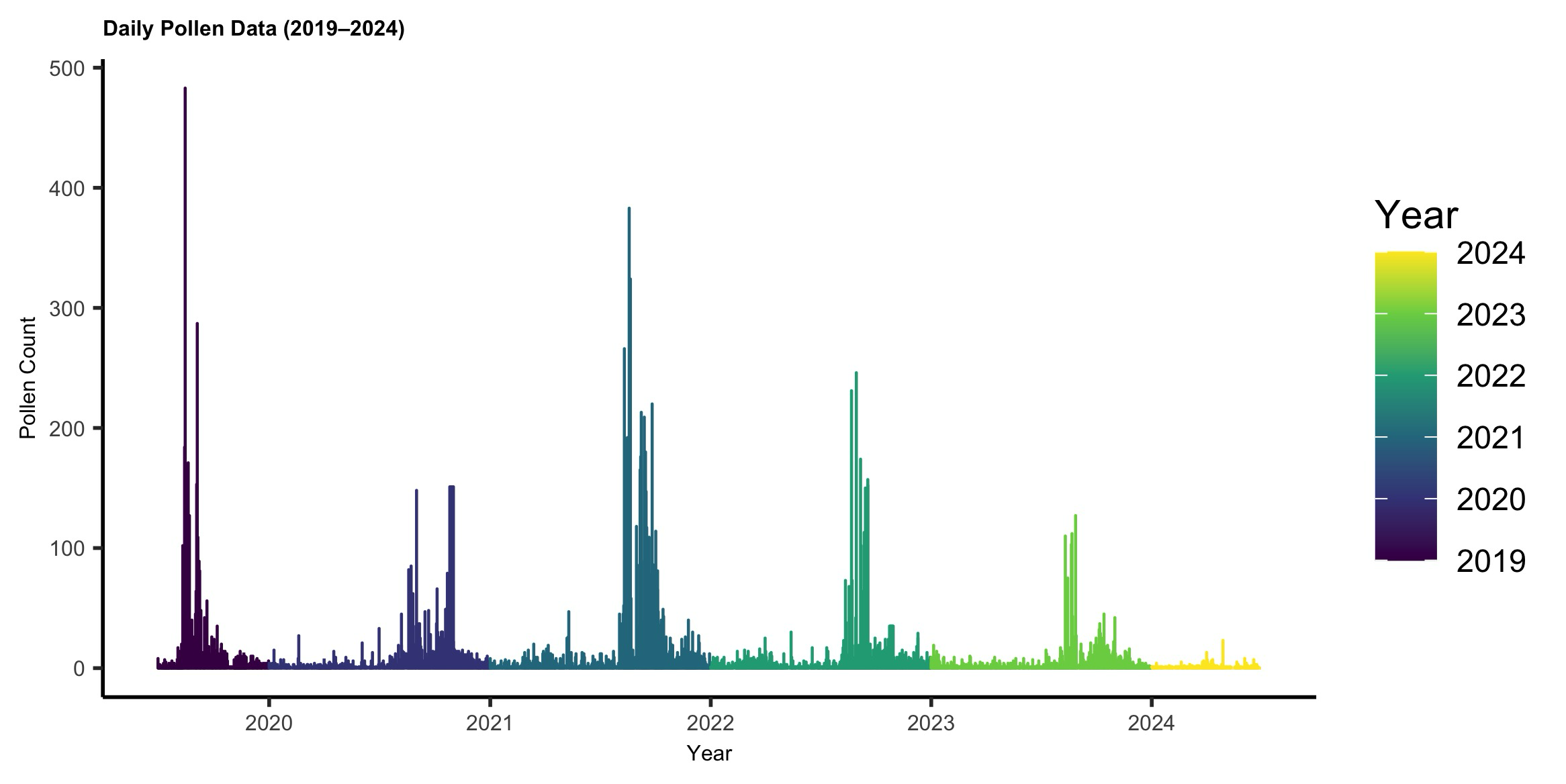 |
| --- |

1. **SAVANNA BIOME (PRETORIA)**

(i)

**
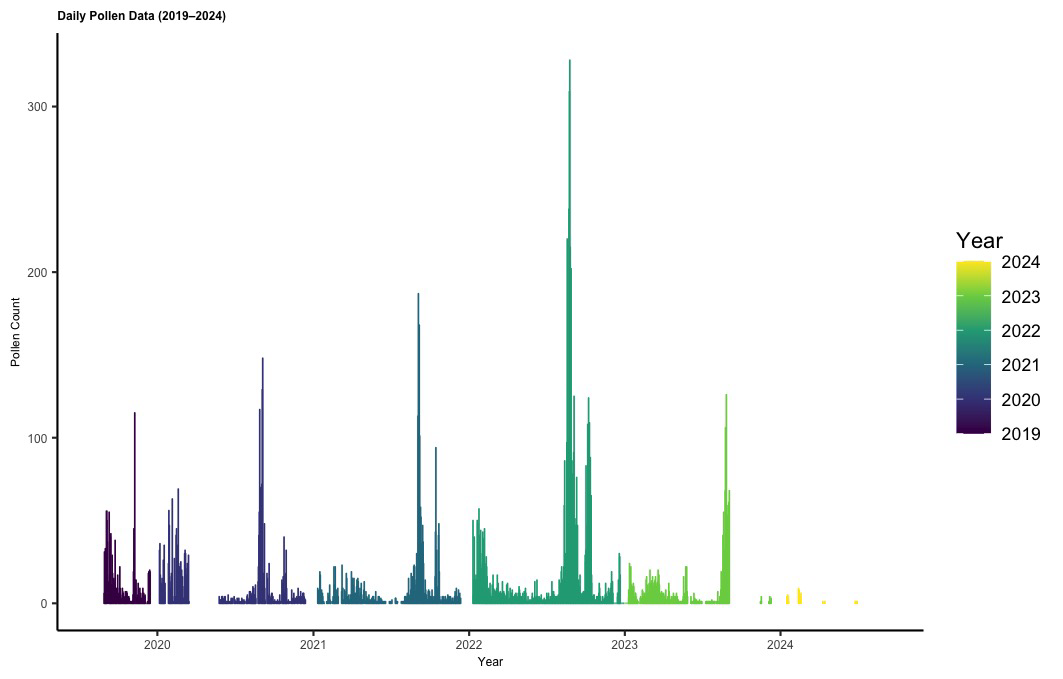
**

(ii)

**
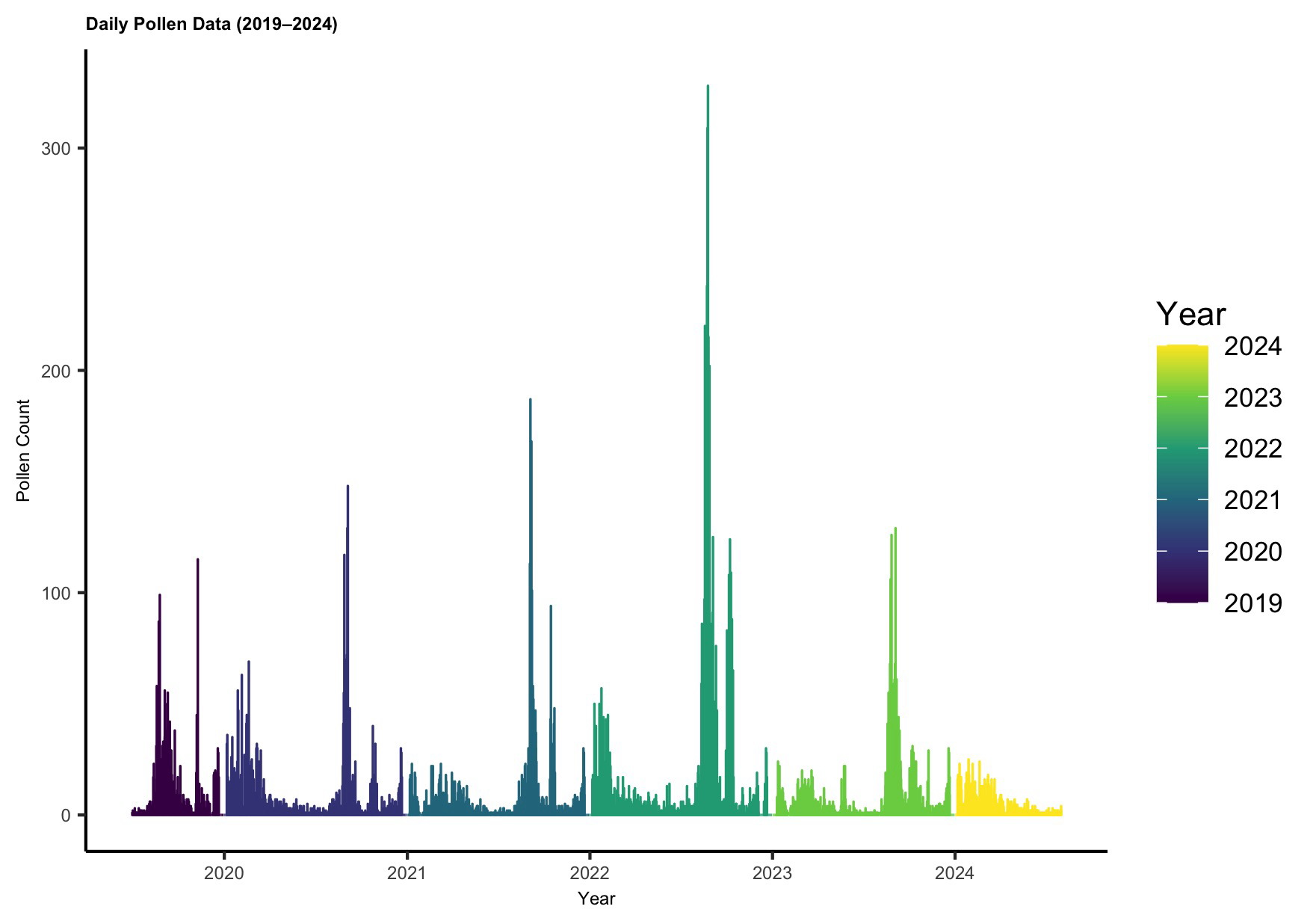
**

1. **GRASSLAND BIOME (JOHANNESBURG)**

**(i)**


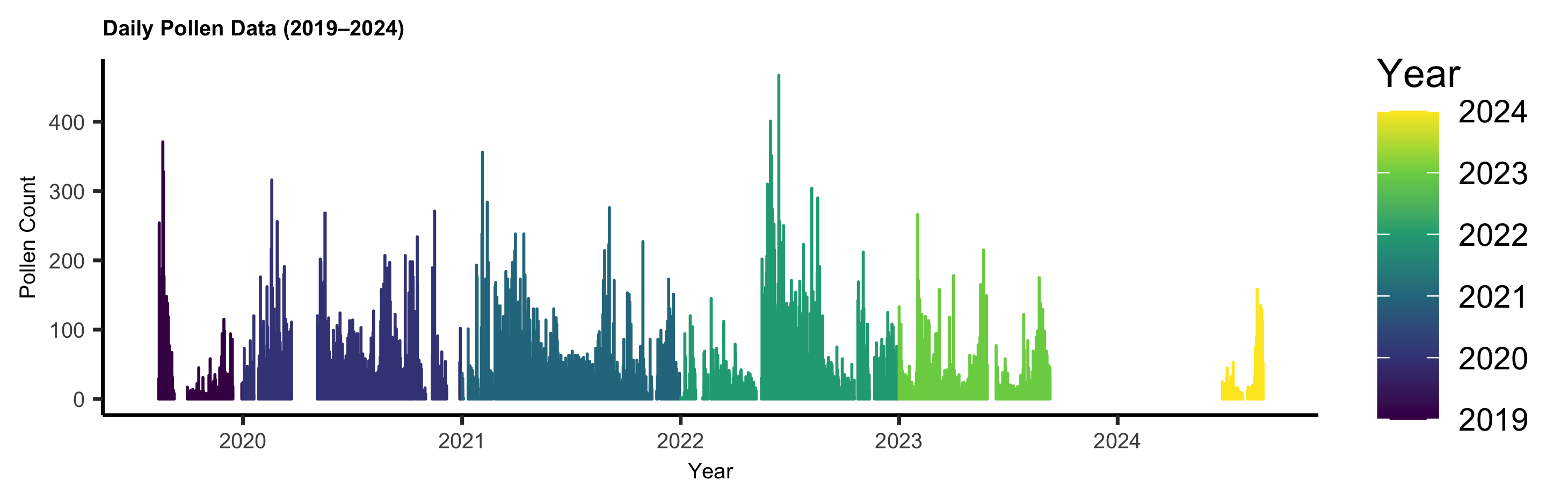


(ii)


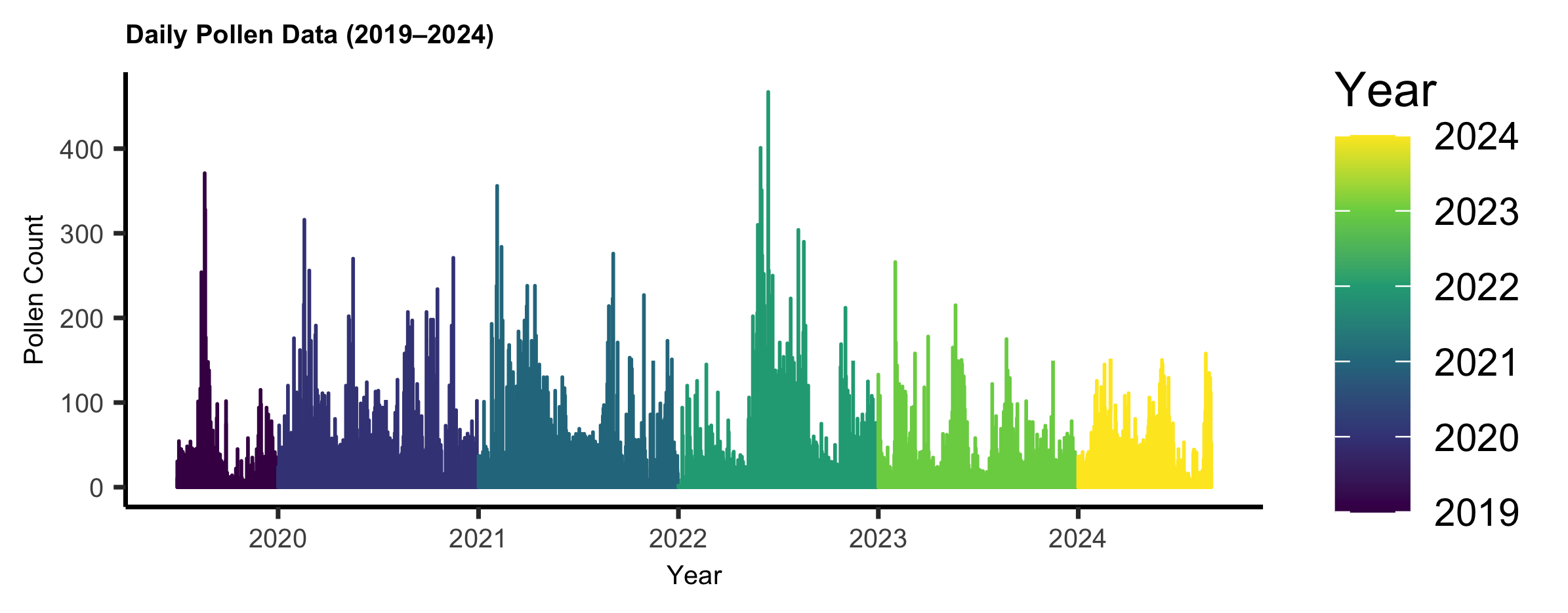


1. **INDIAN OCEAN COASTAL BELT BIOME (DURBAN)**

**(i)**


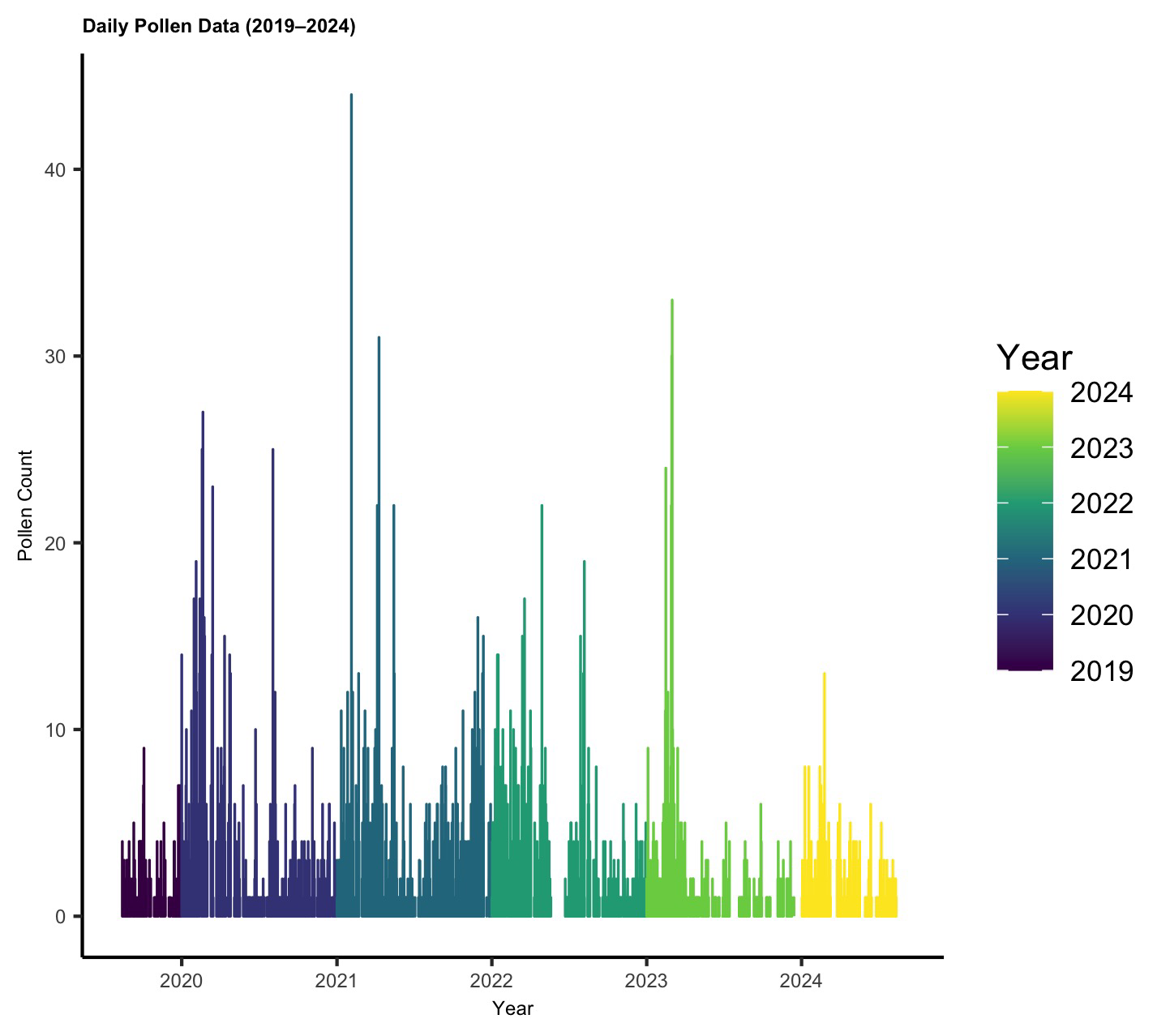


(ii)

**
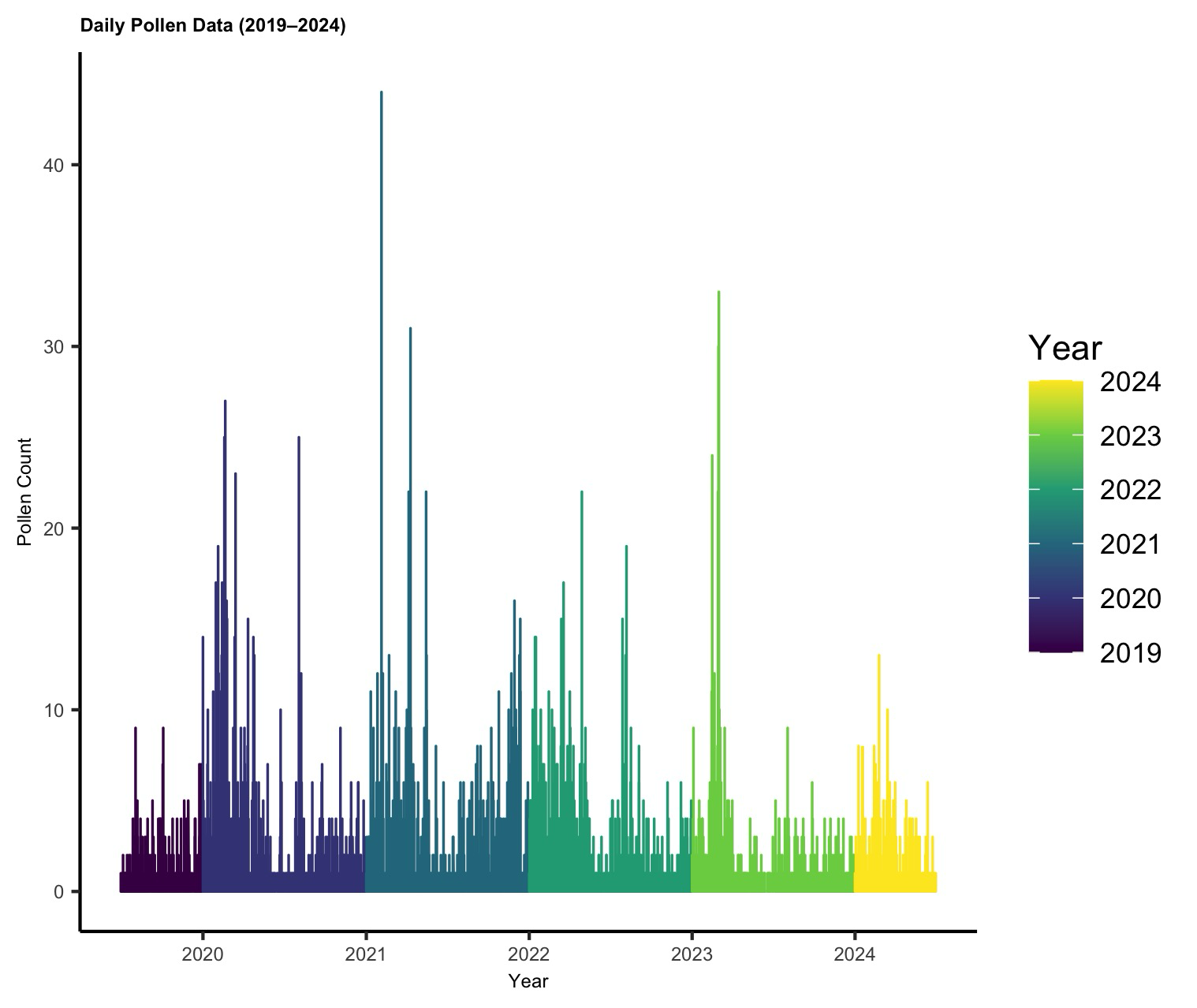
**

1. **SAVANNA BIOME (KIMBERLEY)**

**(i)**


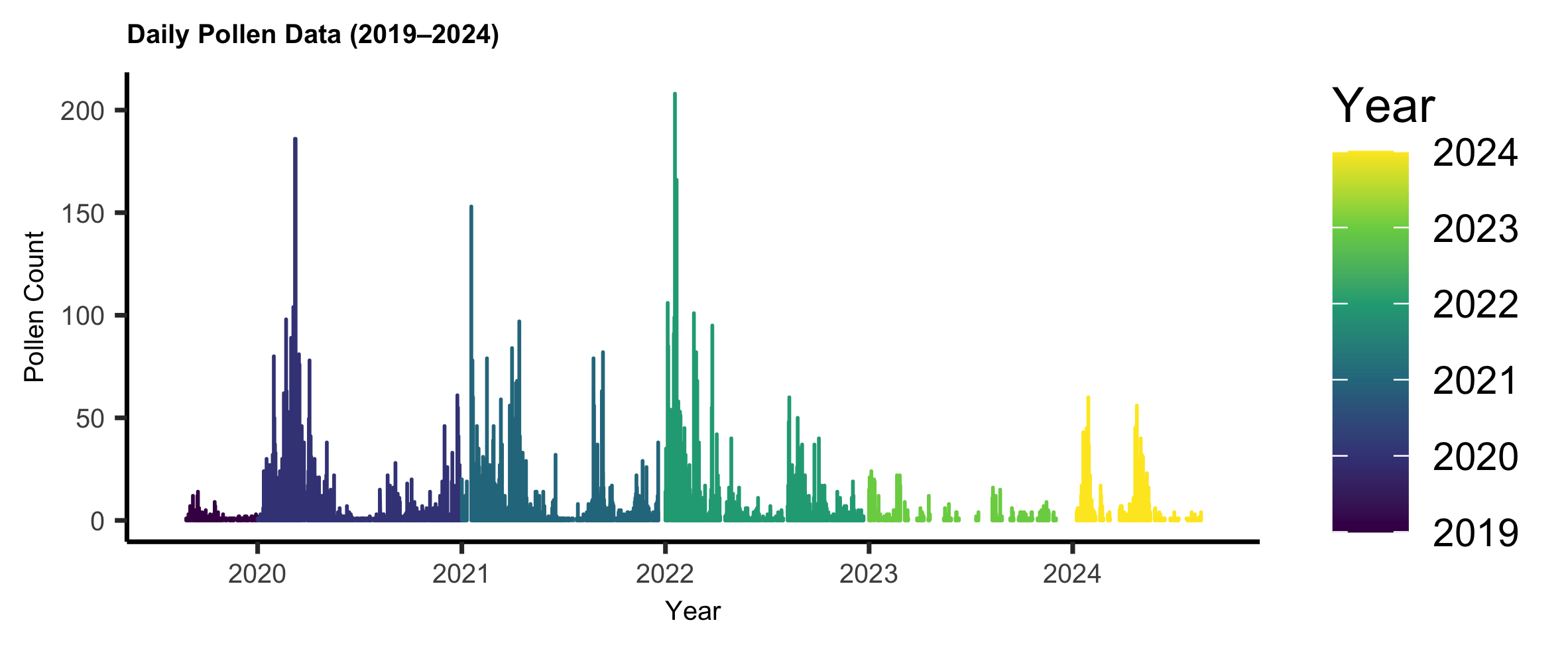


**(ii)**


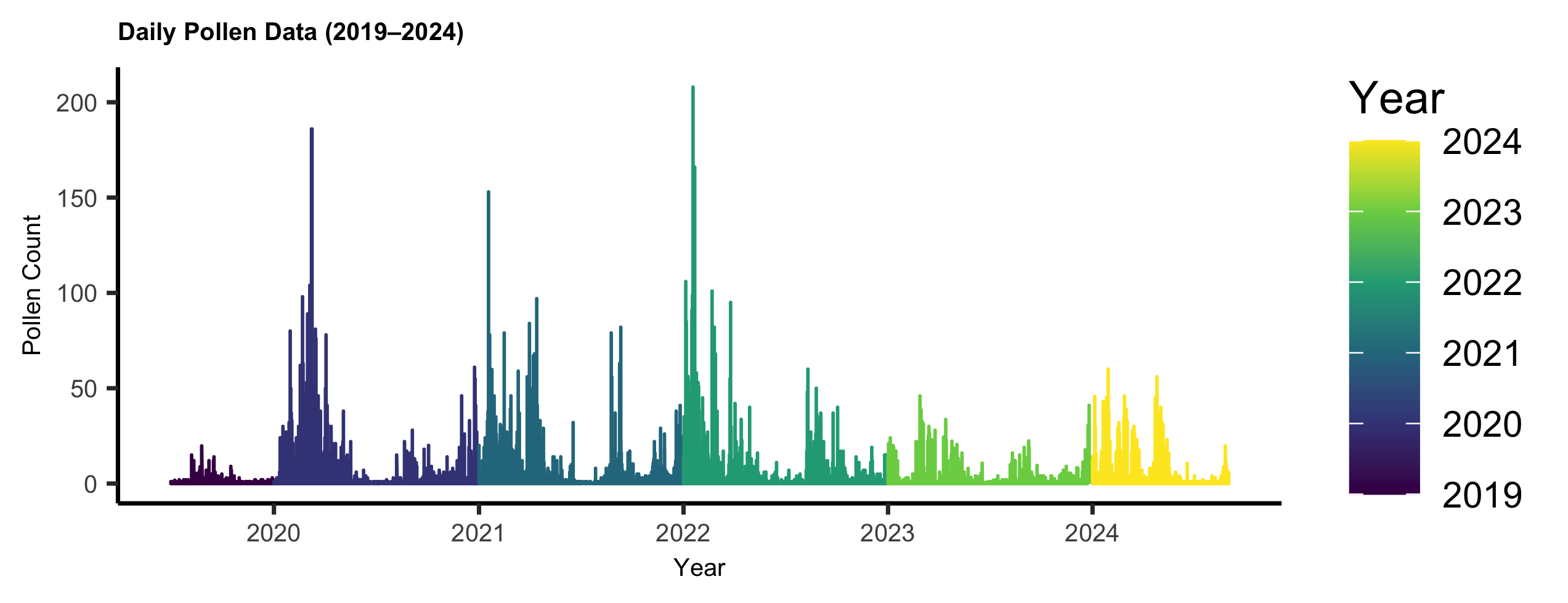


1. **ALBANY THICKET BIOME (GQERBERHA)**

(i)


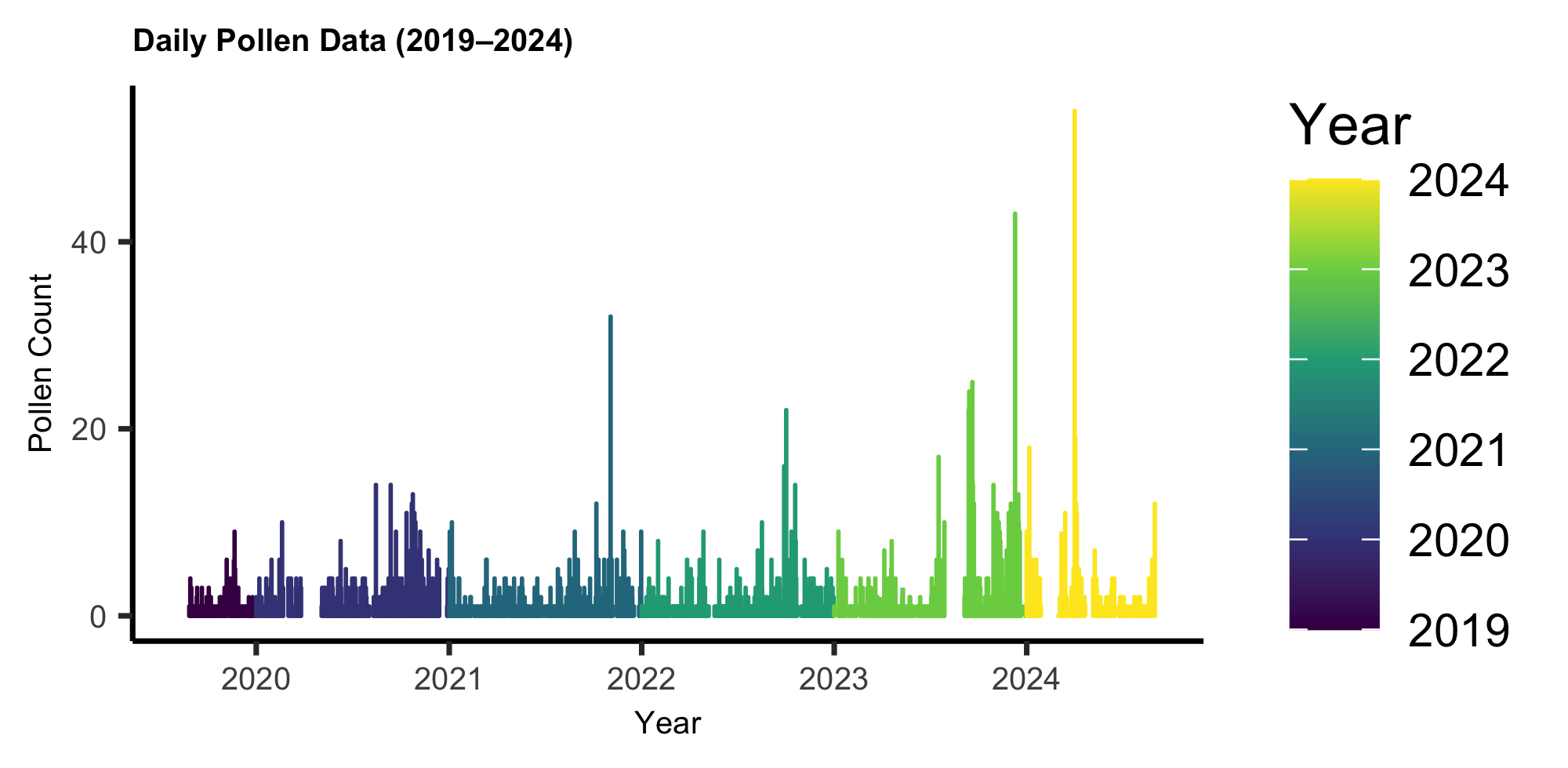


(ii)


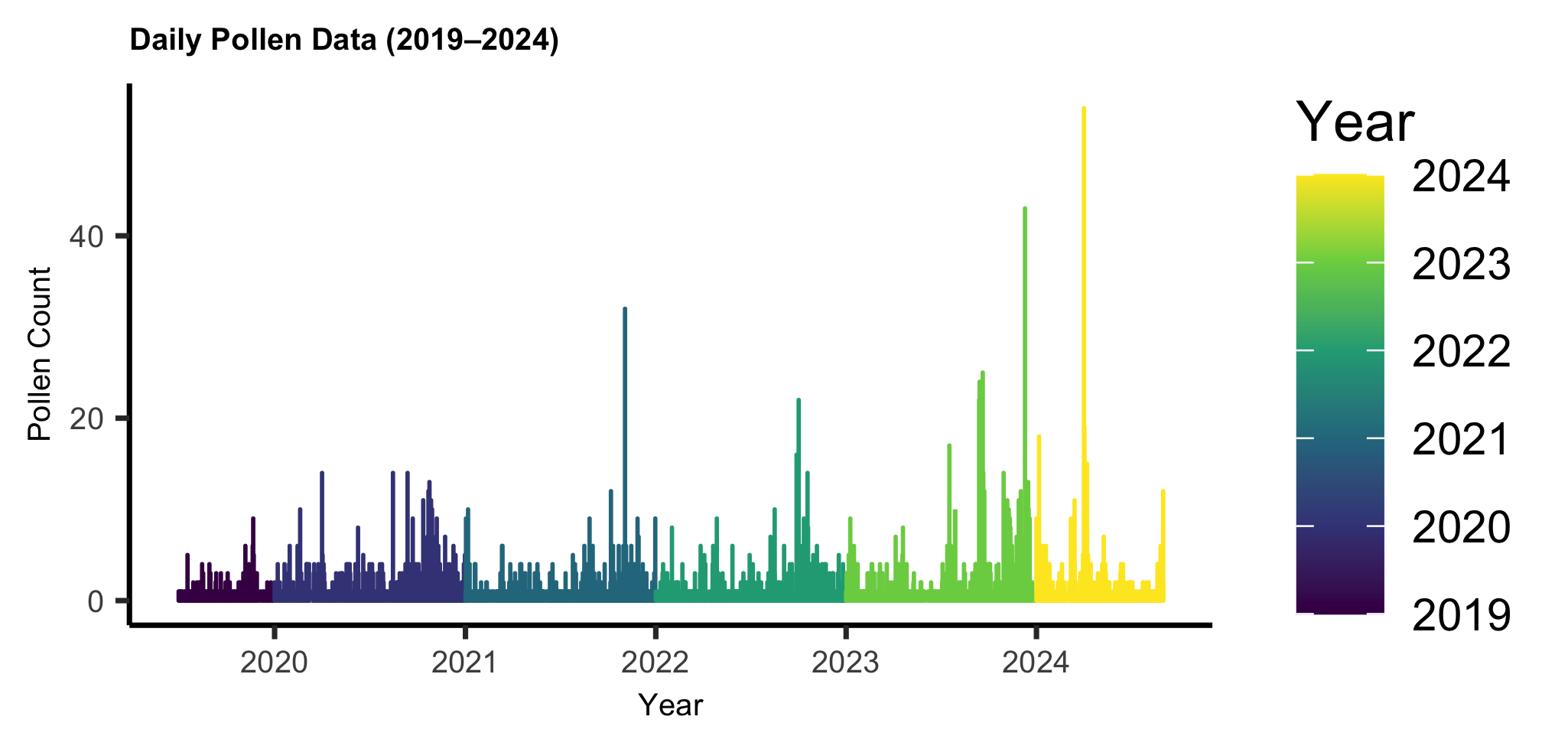


1. **GRASSLAND (BLOEMFONTEIN)**

(i)


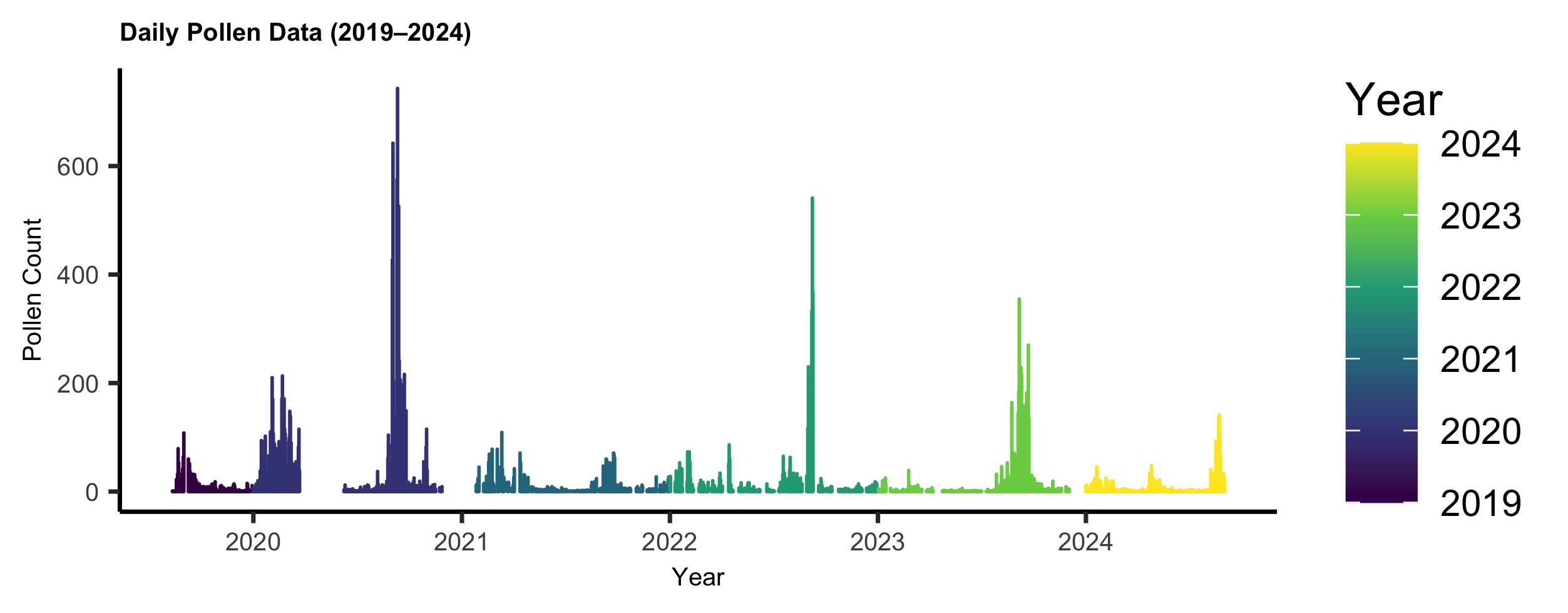


(ii)


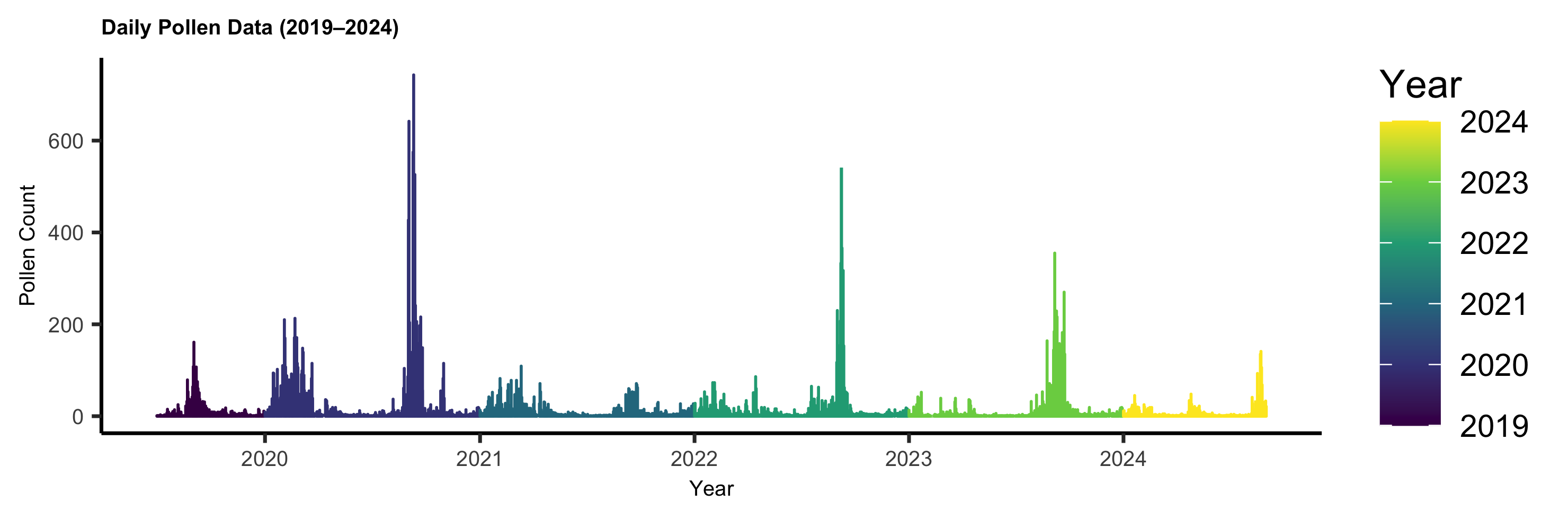


**Fig. S2** The number of pollen taxa detected at each city across the five years and grouped by month.

1. **FYNBOS (CAPE TOWN)**

**
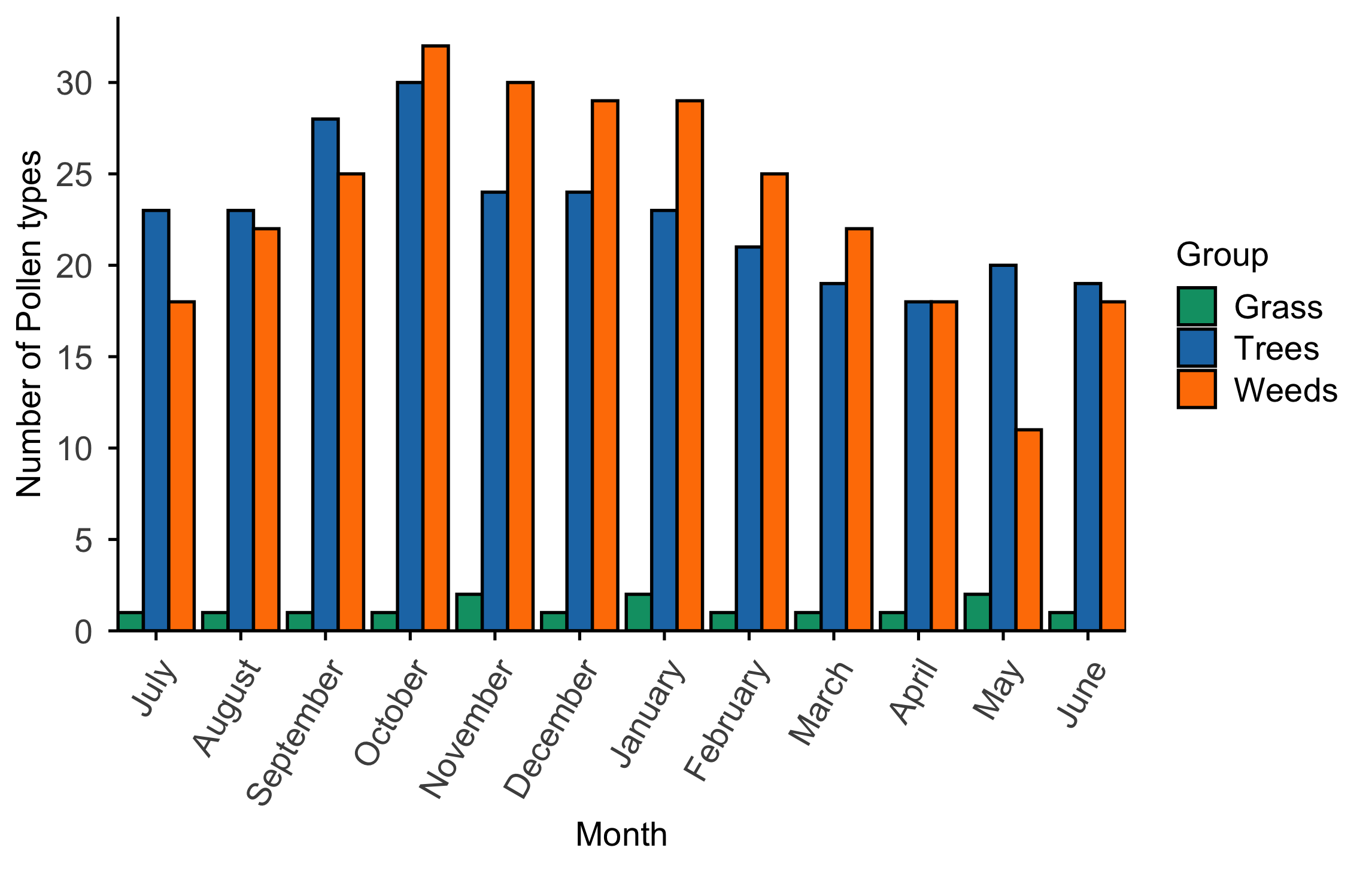
**

1. **INDIAN OCEAN COASTAL BELT (DURBAN)**


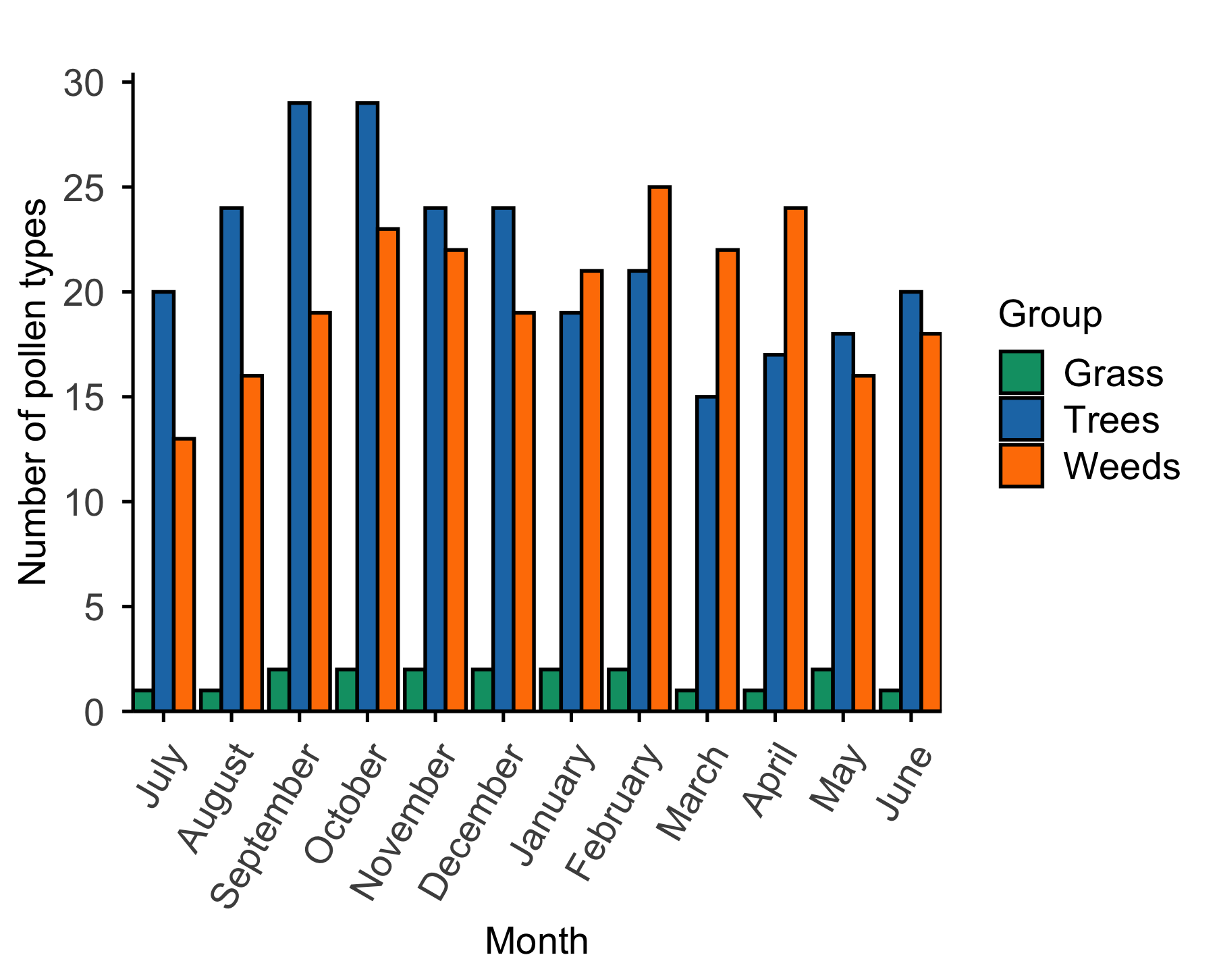


1. **ALBANY THICKET (GQEBERHA)**

**
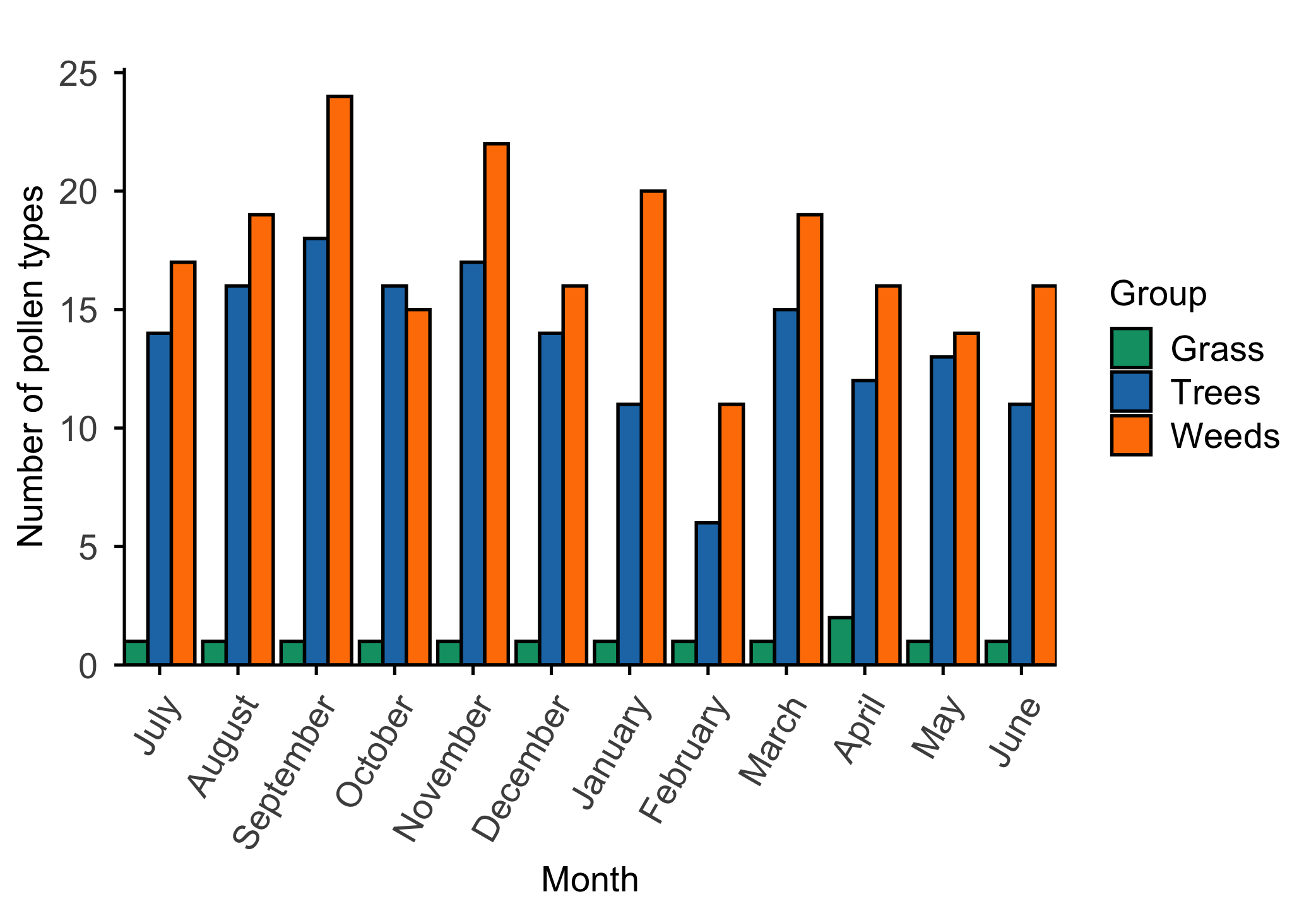
**

1. **GRASSLAND (JOHANNESBURG)**


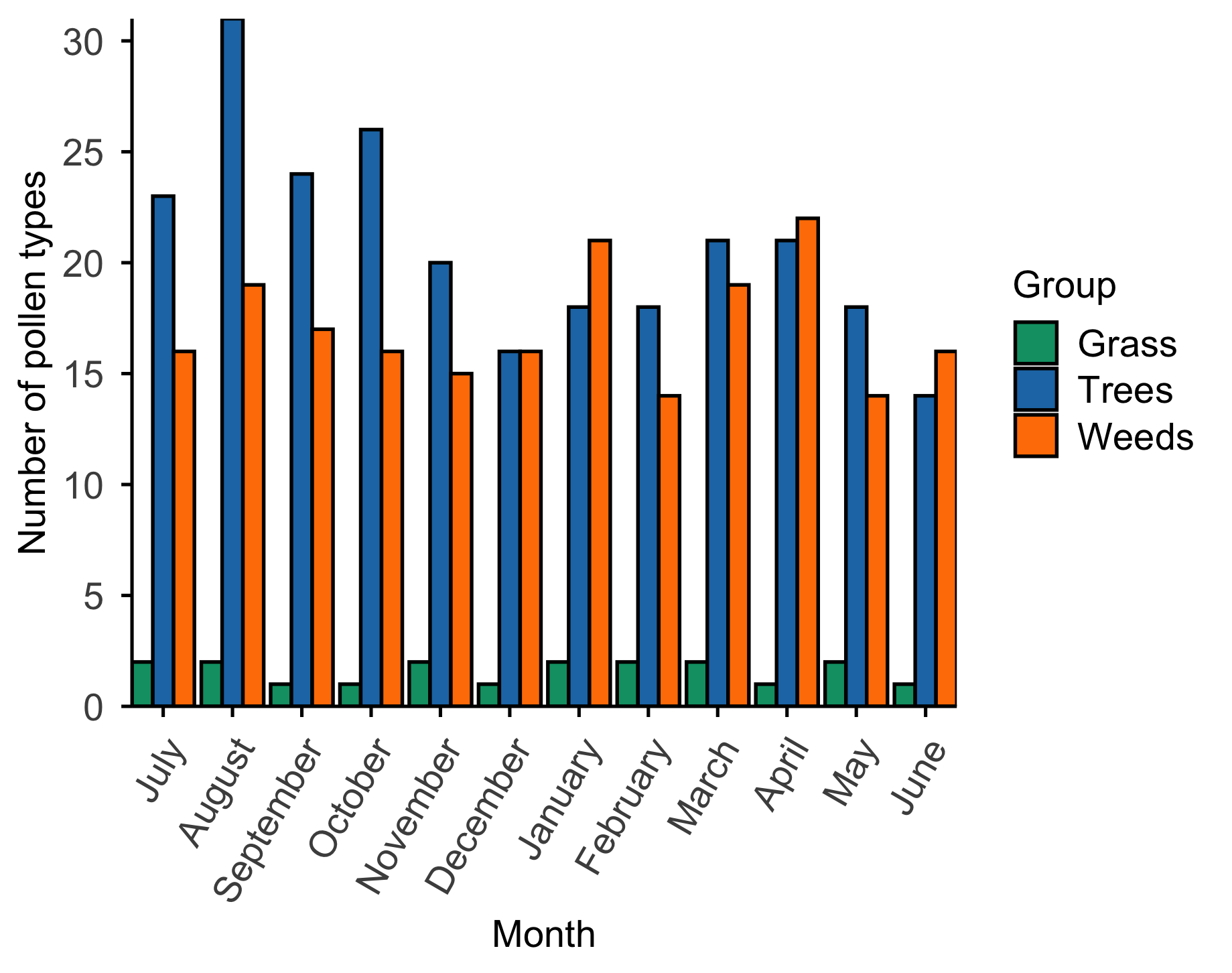


1. **SAVANNA (PRETORIA)**

**
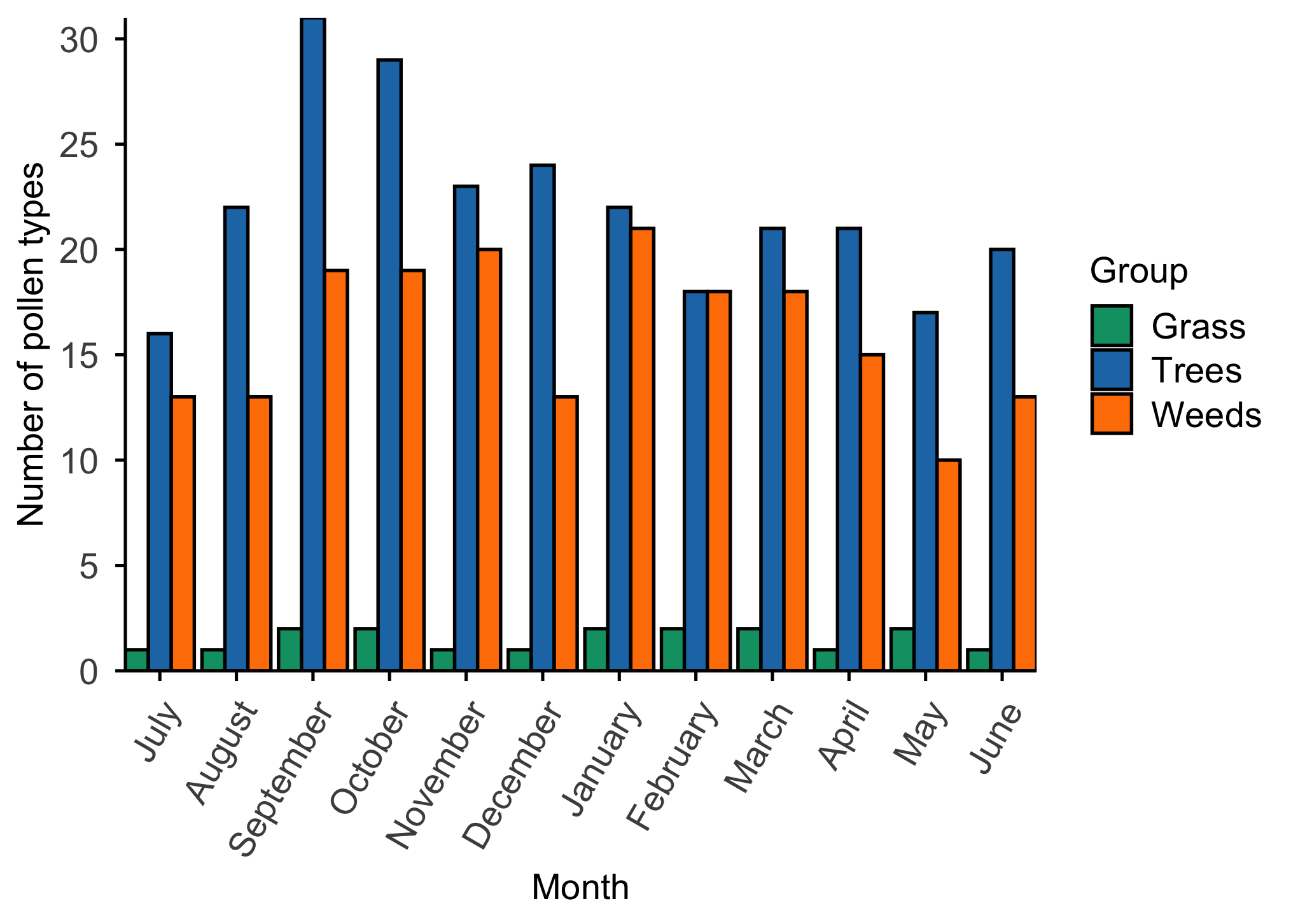
**

1. **GRASSLAND (BLOEMFONTEIN)**

**
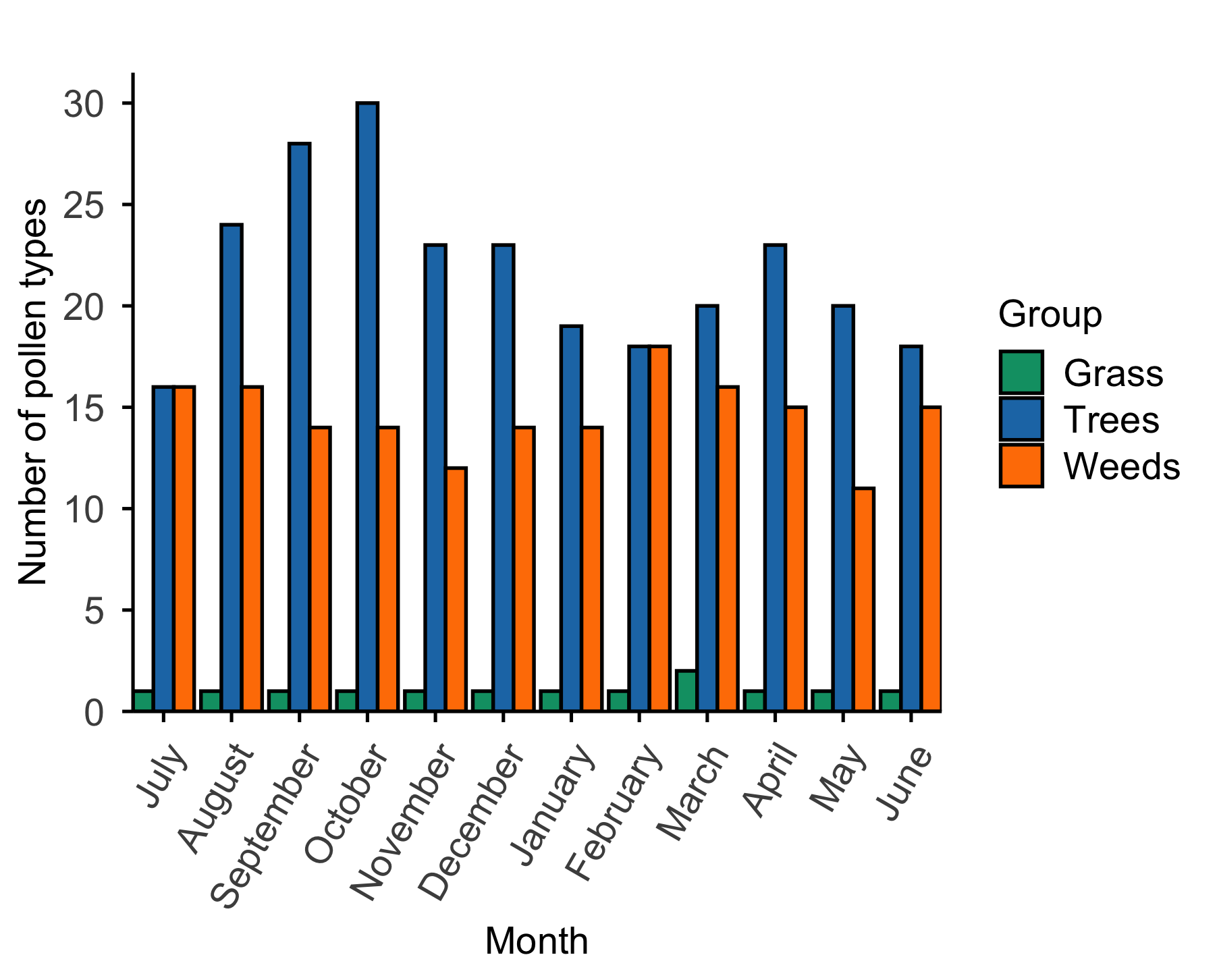
**

1. **SAVANNA (KIMBERLEY)**

**
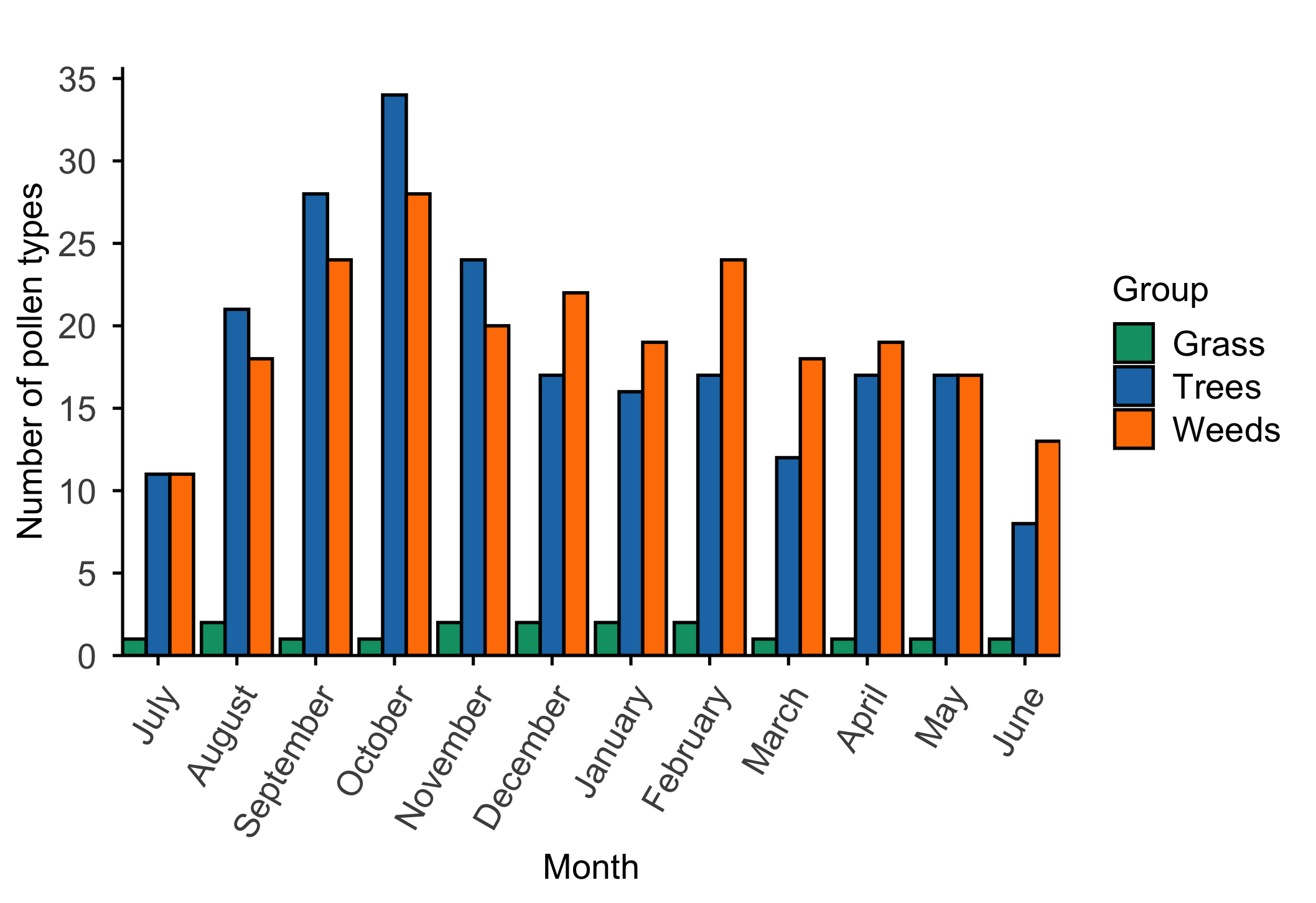
**

**Fig. S3** List of the top 20 grass species that occur in each province of South Africa based on the global biodiversity information facility (35).


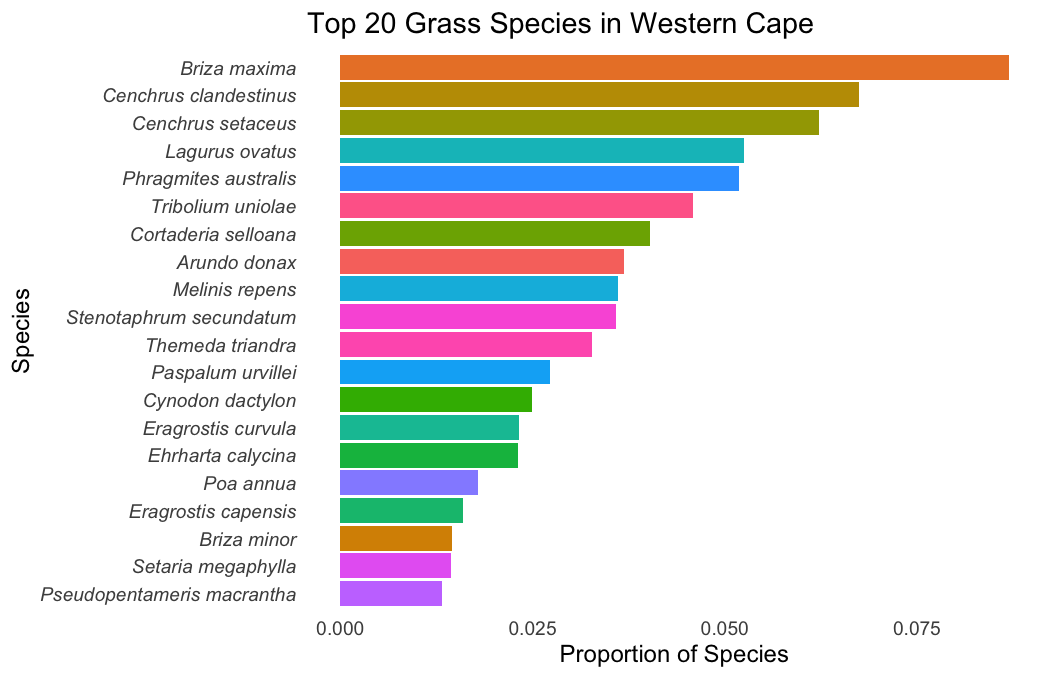


(b)


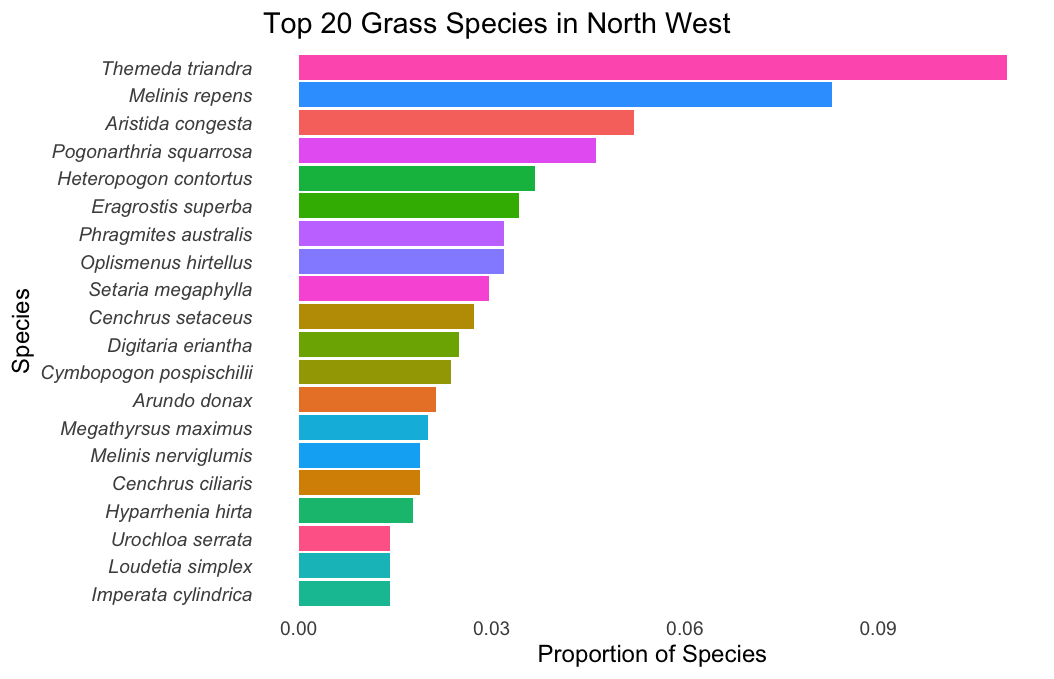


(c)


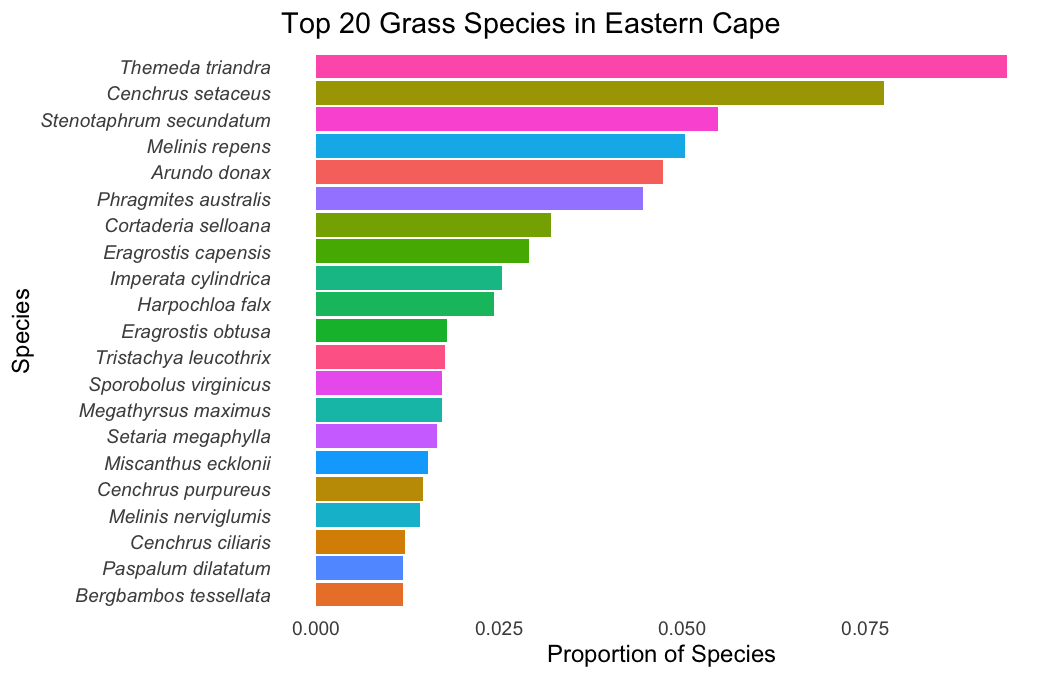


(d)


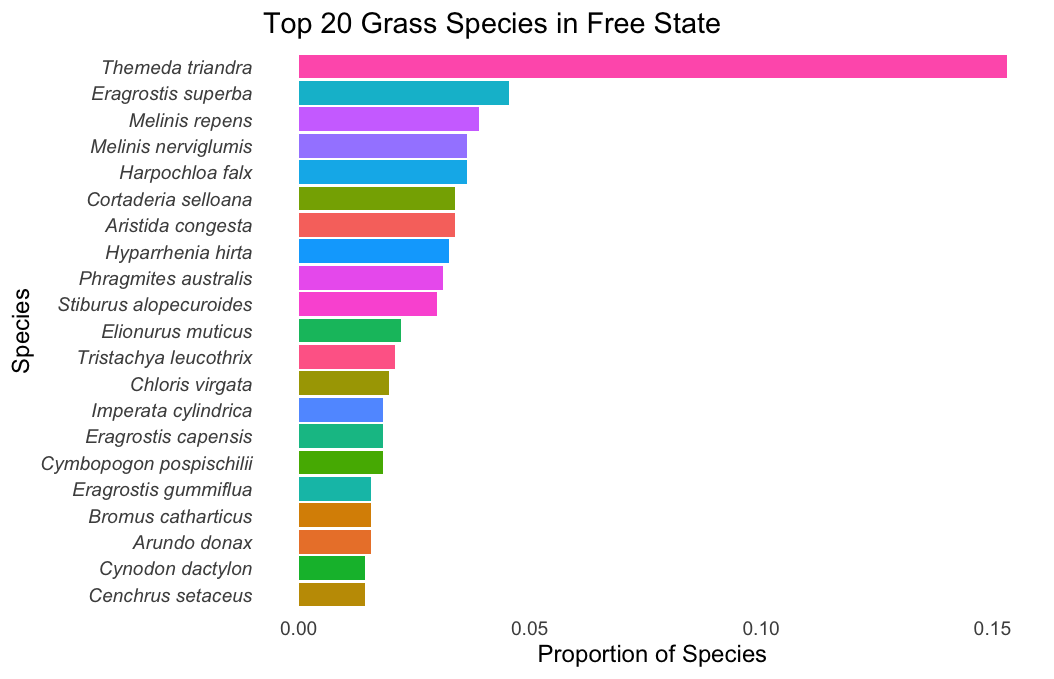


(e)


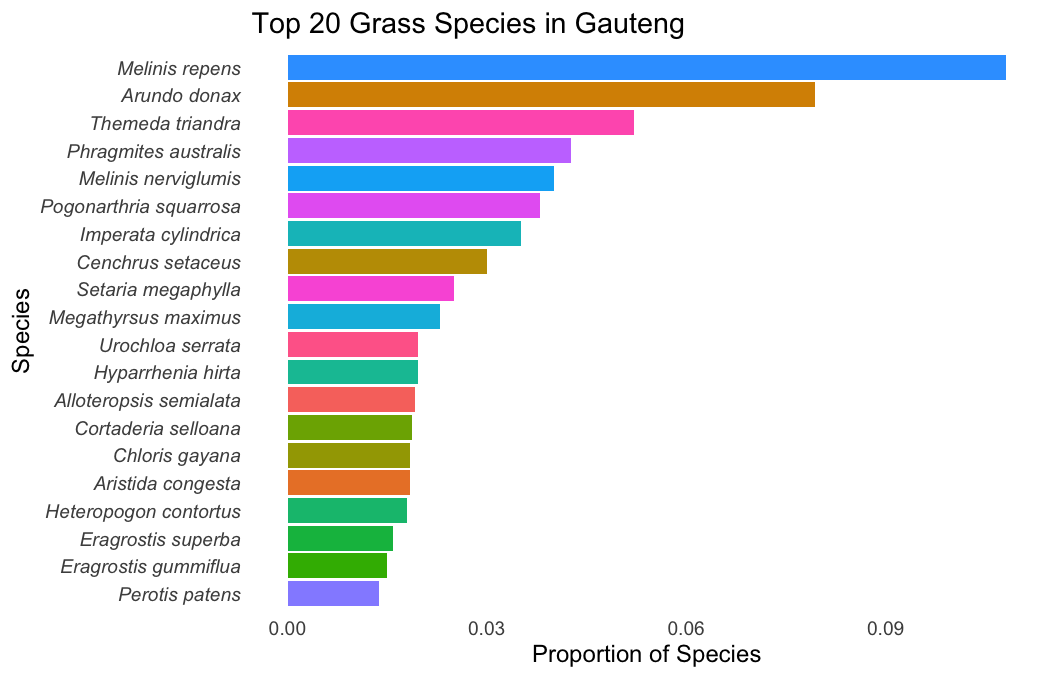


(f)


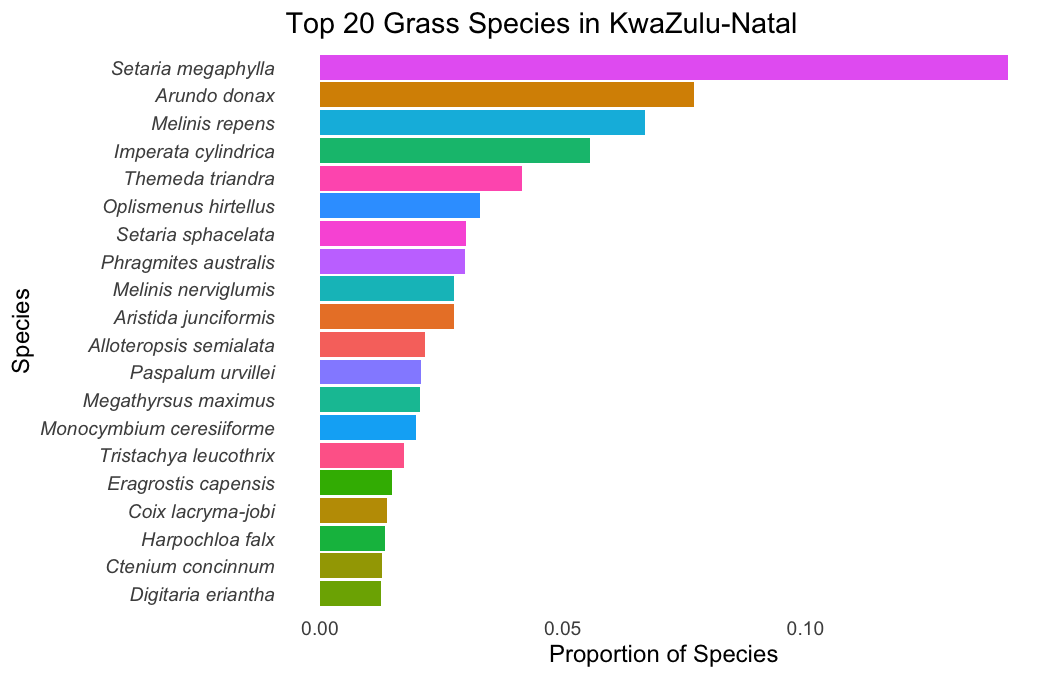


**(g)**


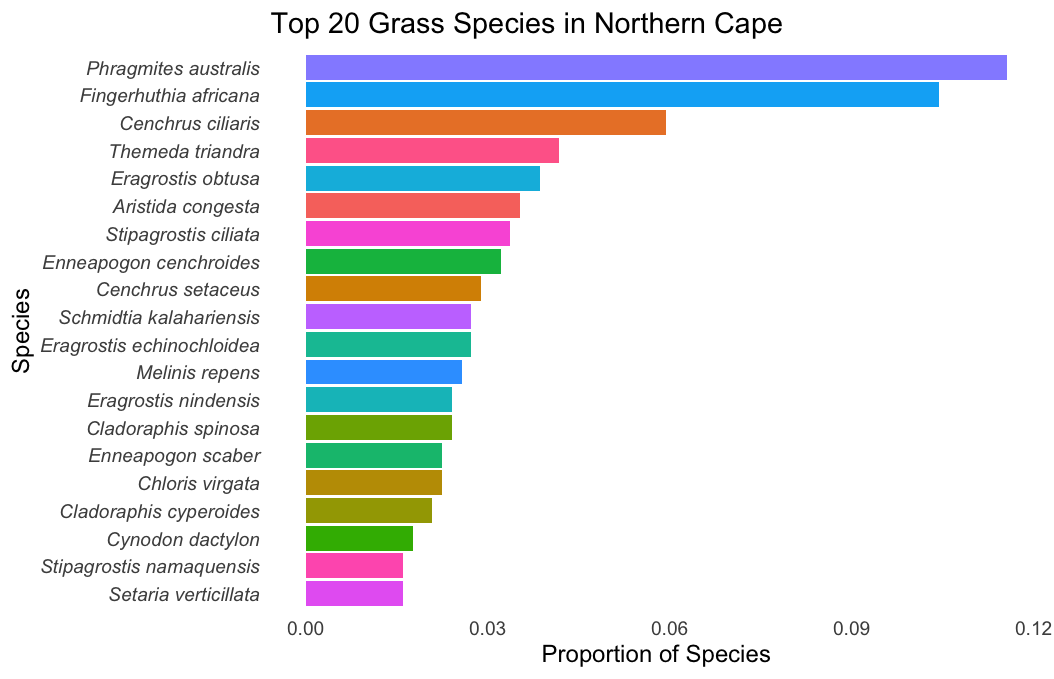


**Fig. S4** The averaged pollen integral across the biomes in South Africa.


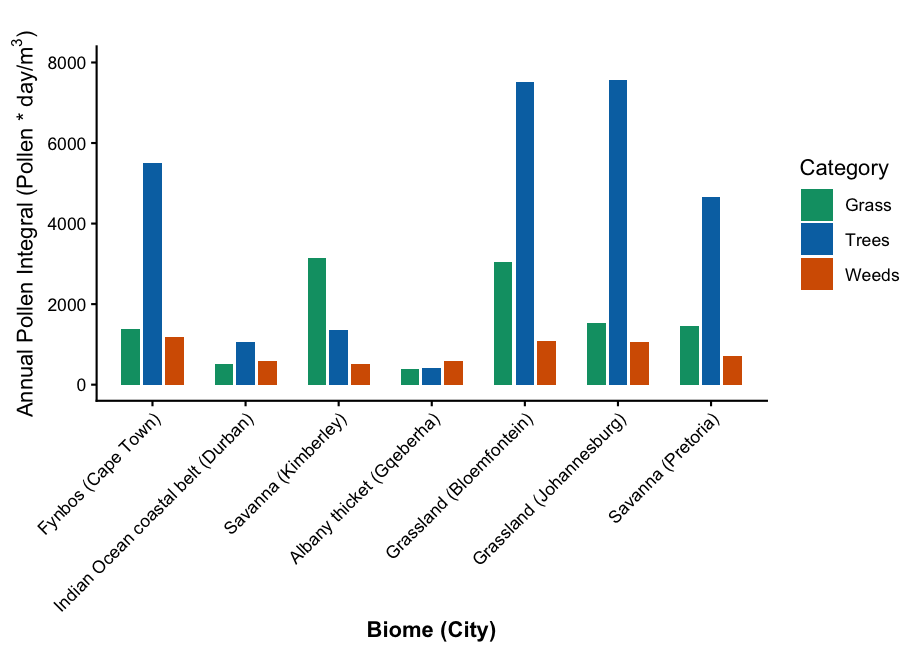


**Fig. S5** Pollen trends of taxa in seasons with high and low pollen concentrations. The graphs shows pollen concentration of different taxa in years with the highest and the lowest pollen levels (A-G).

1. **FYNBOS (CAPE TOWN)**


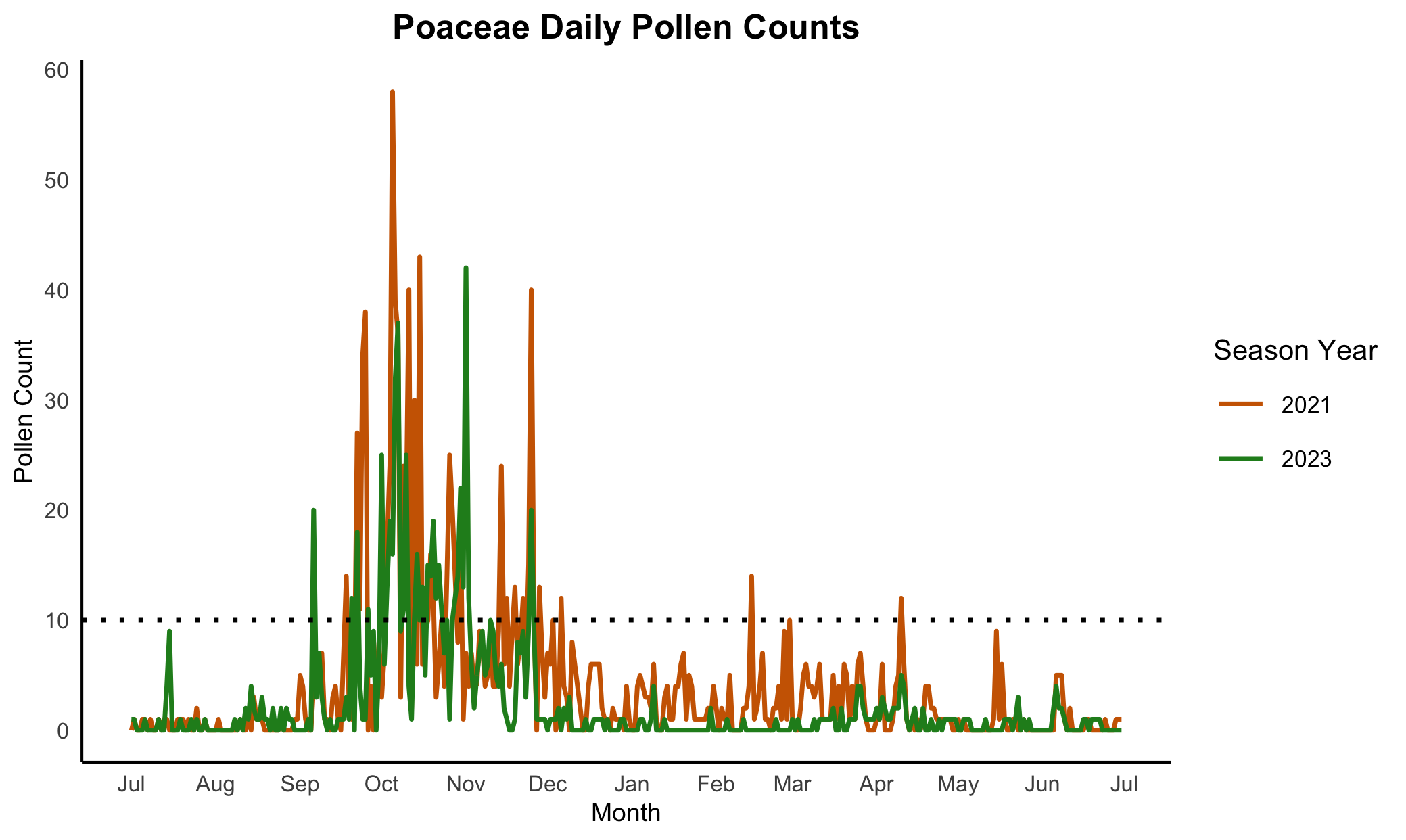


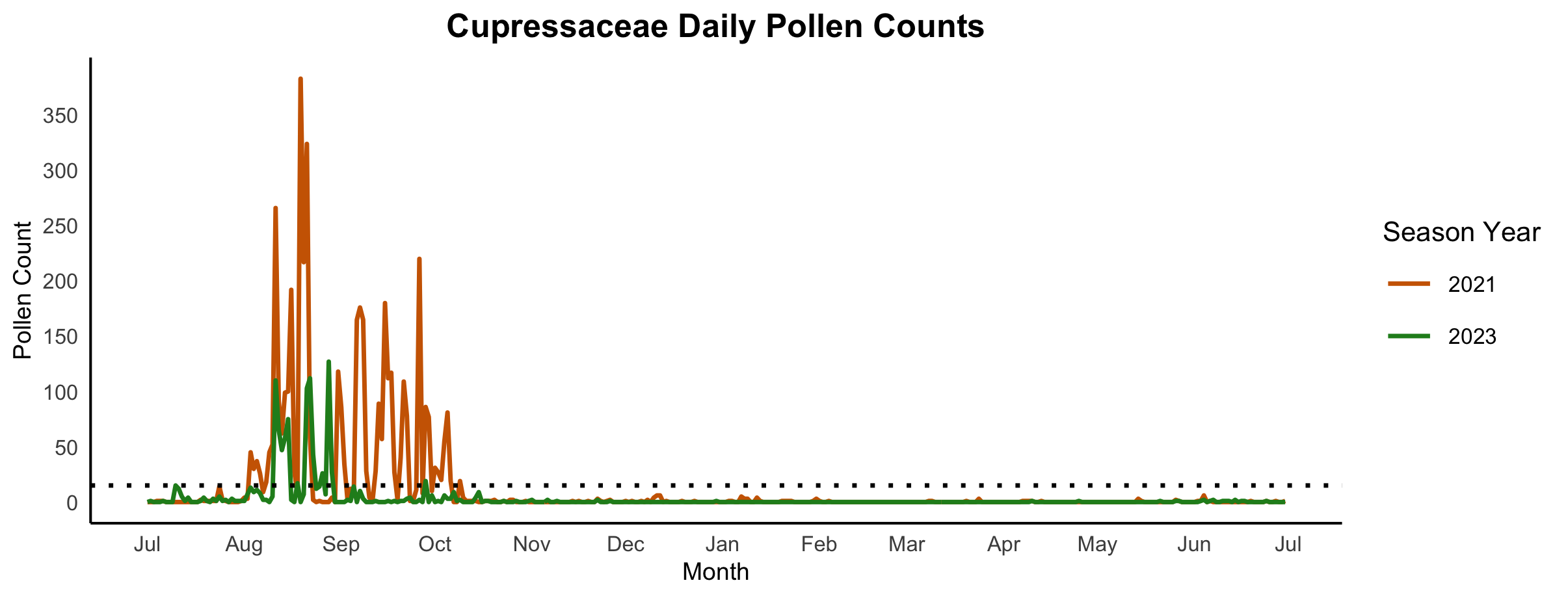


1. **GRASSLAND (JOHANNESBURG)**


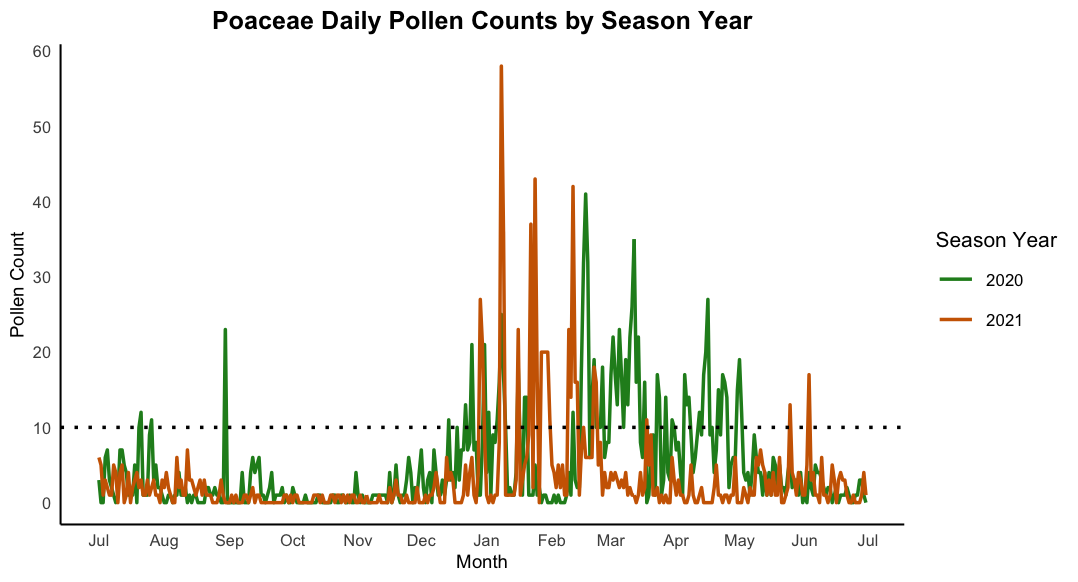


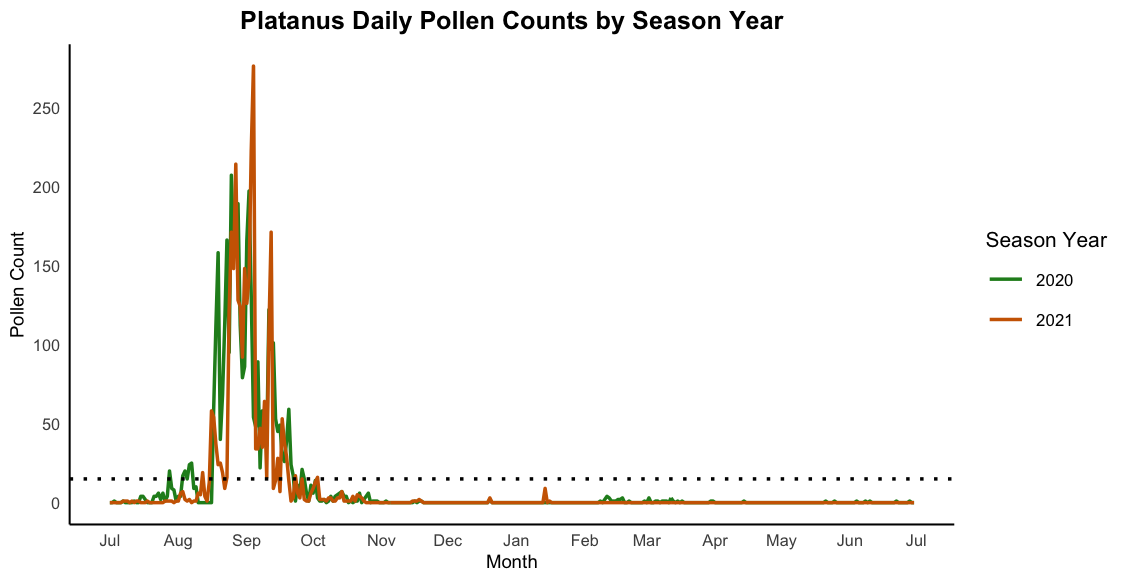


1. **SAVANNA (PRETORIA)**


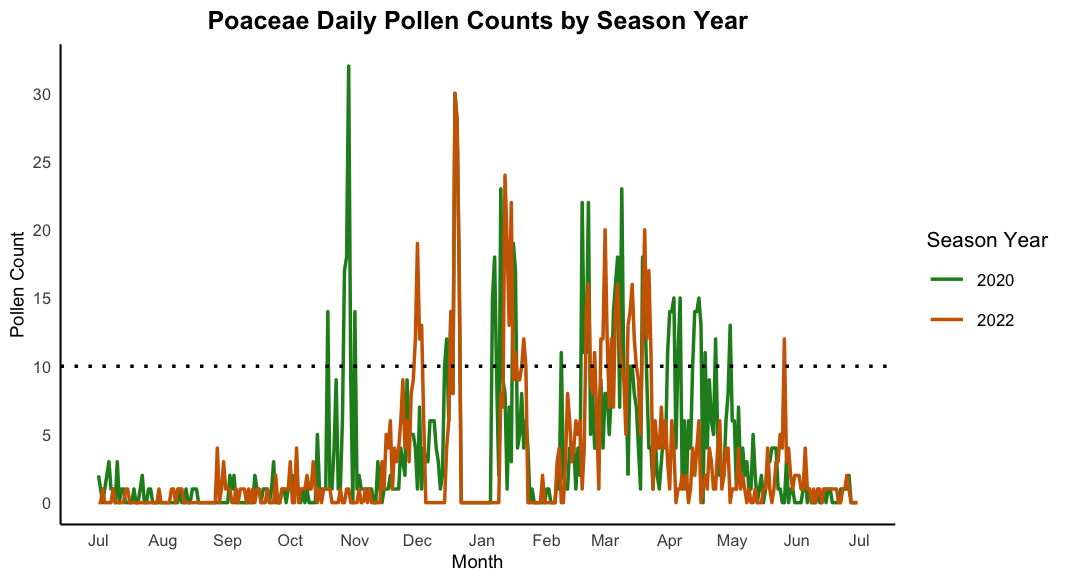


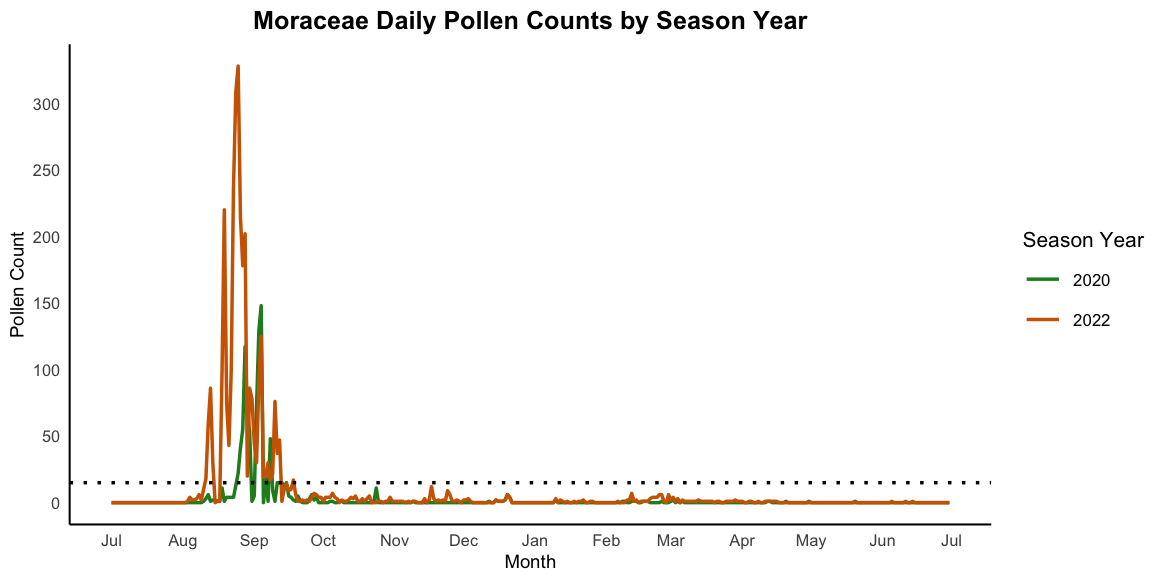


1. **INDIAN OCEAN COASTAL BELT (DURBAN)**


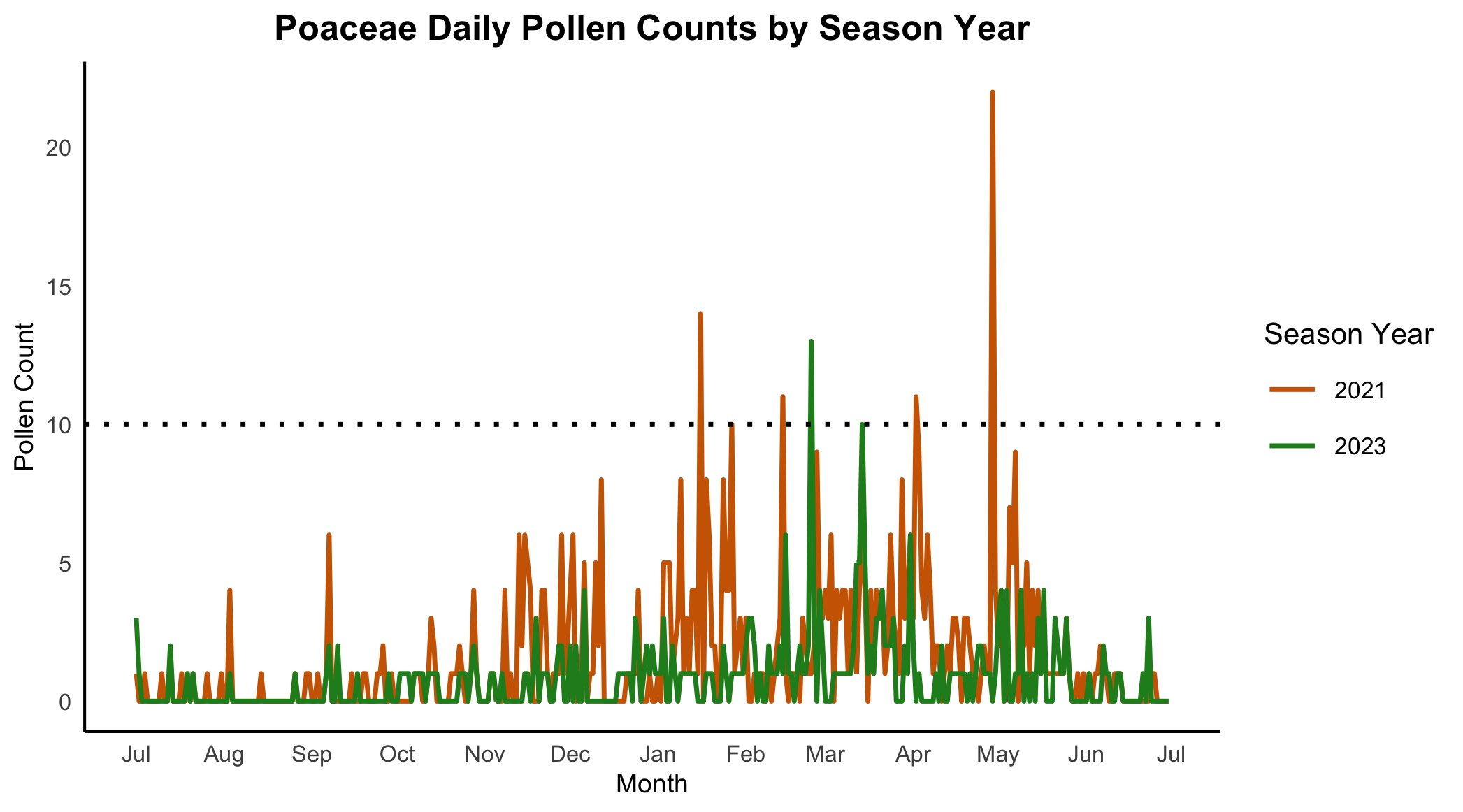


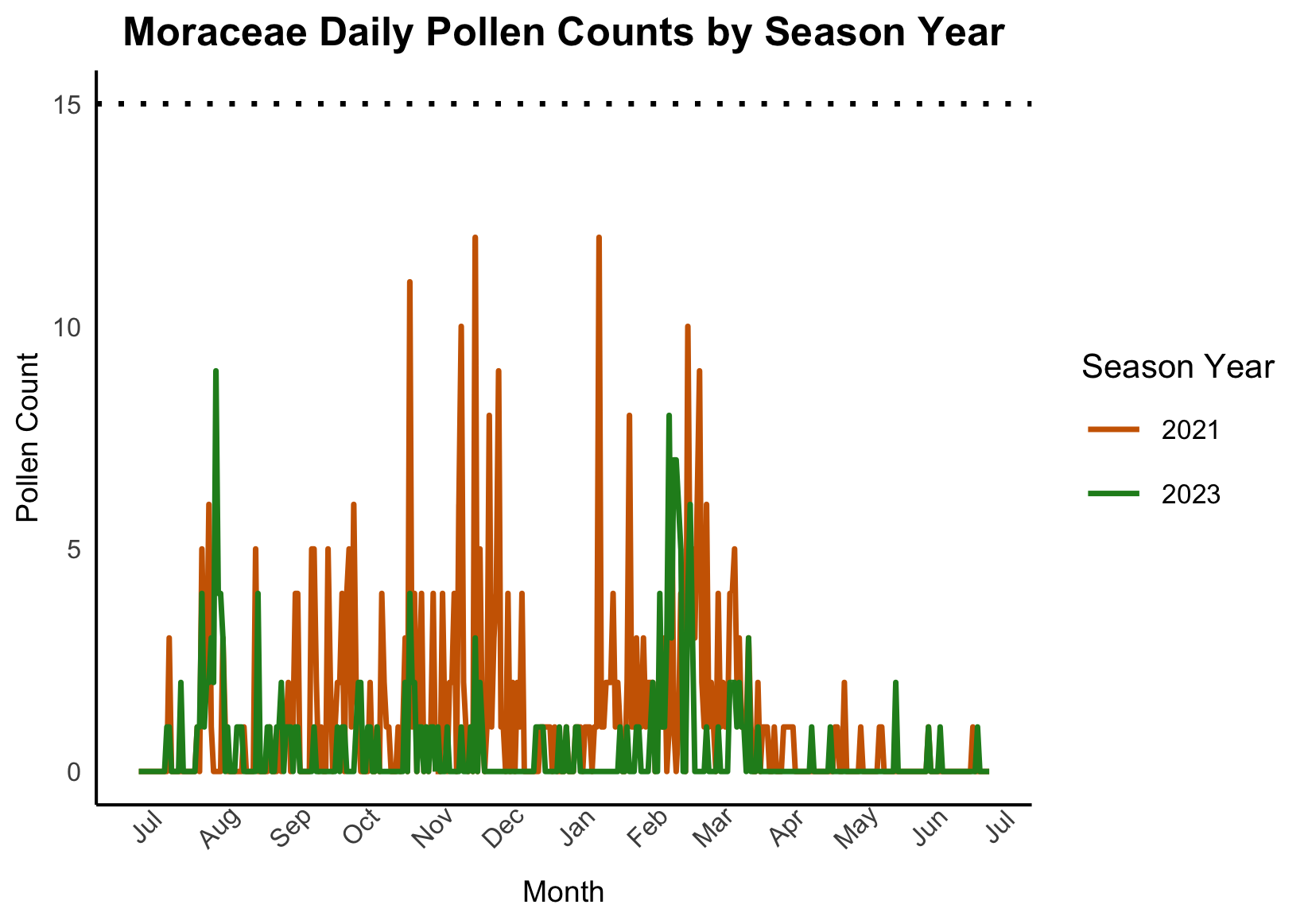


1. **GRASSLAND (BLOEMFONTEIN)**


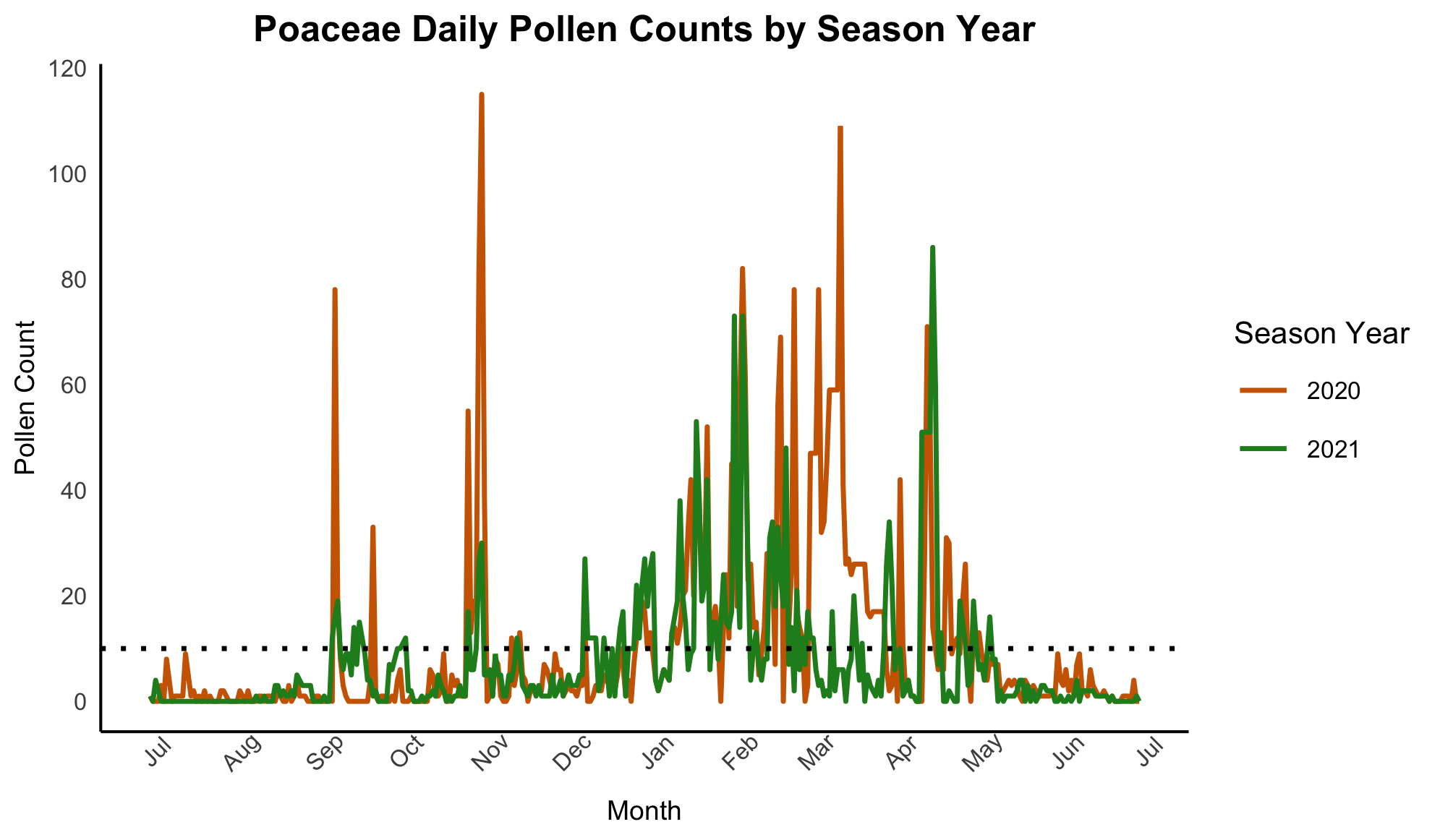


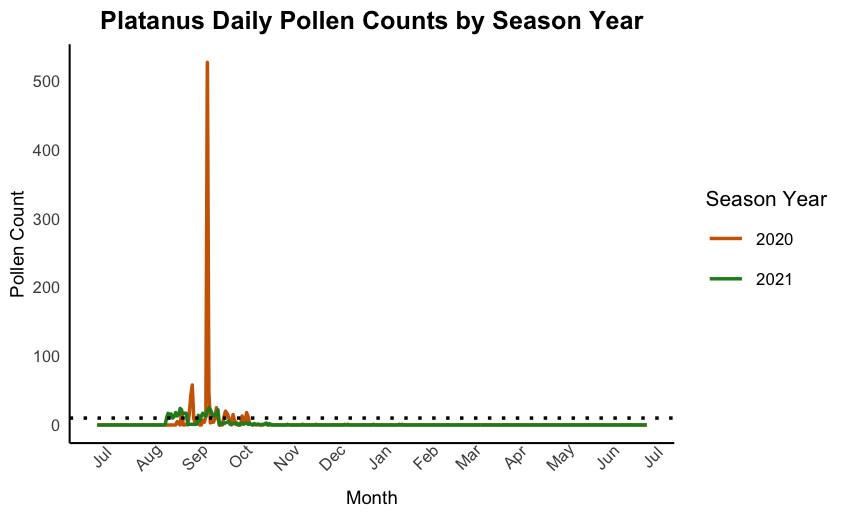


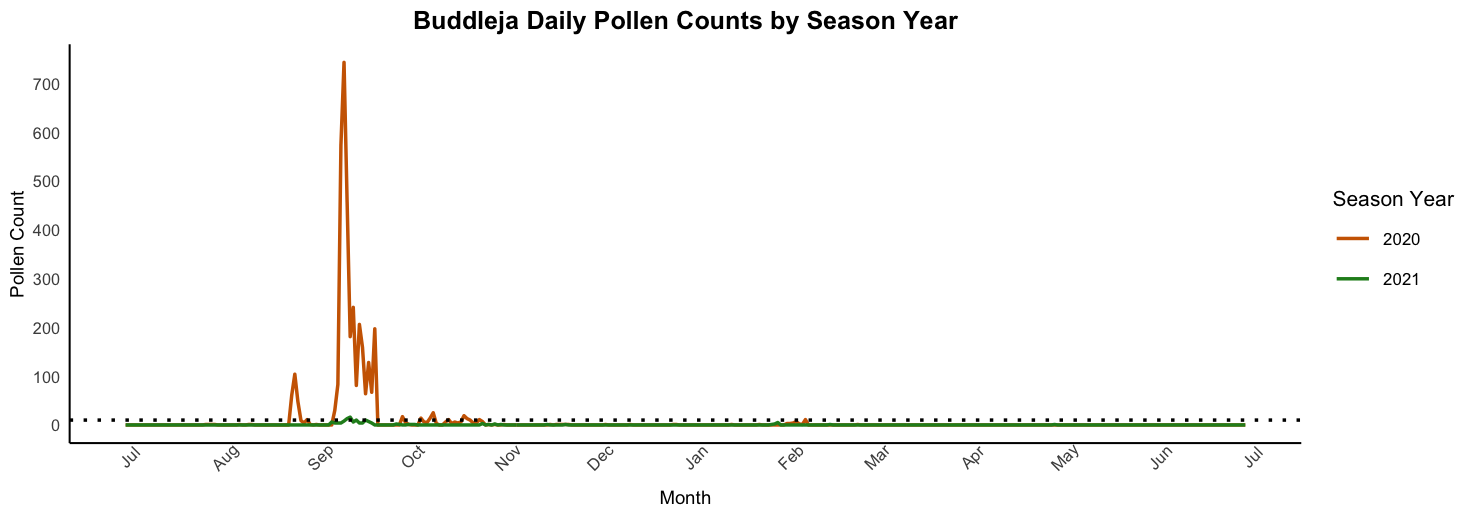


1. **ALBANY THICKET (GQEBERHA)**


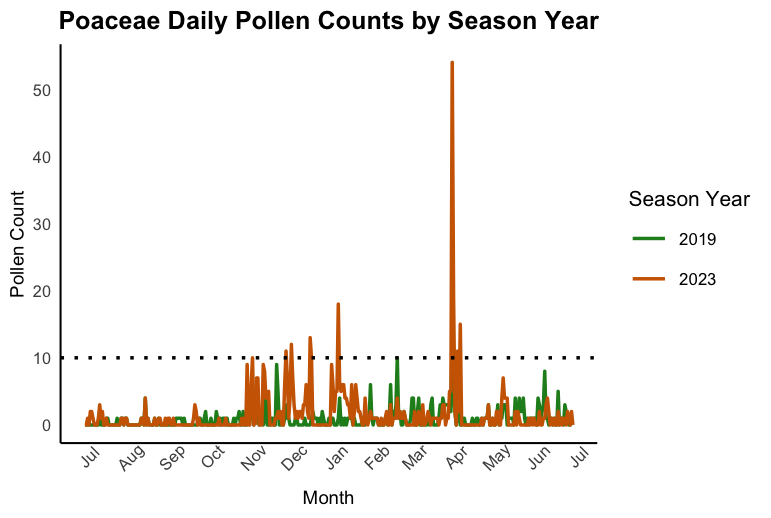


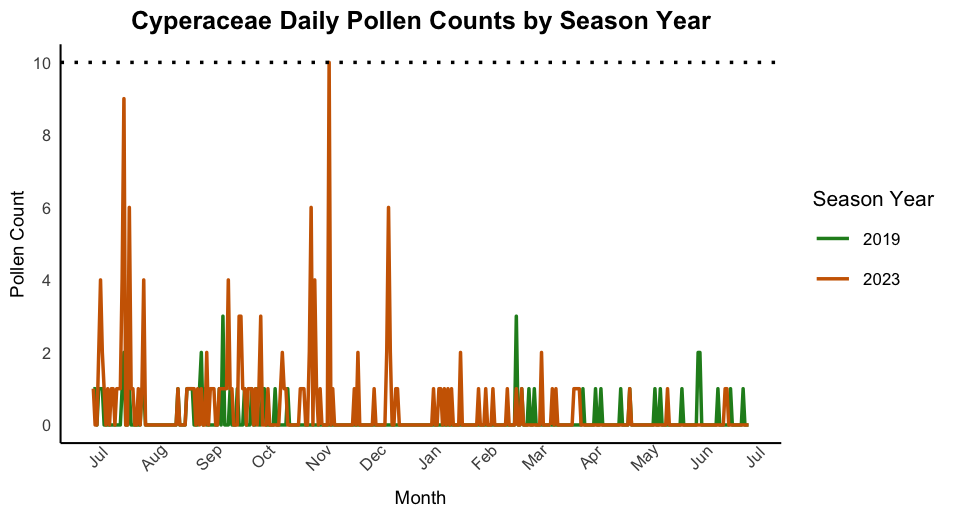


1. **SAVANNA (KIMBERLEY)**


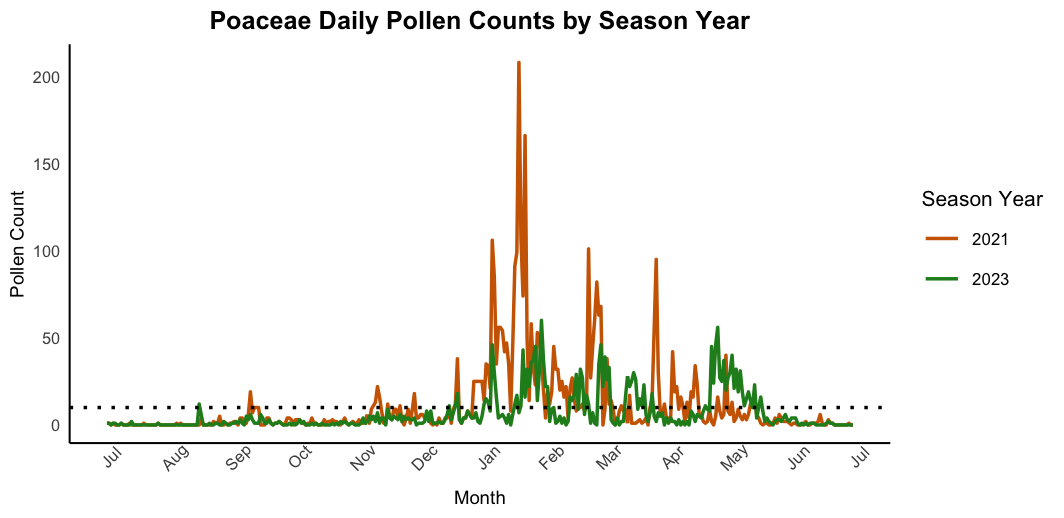


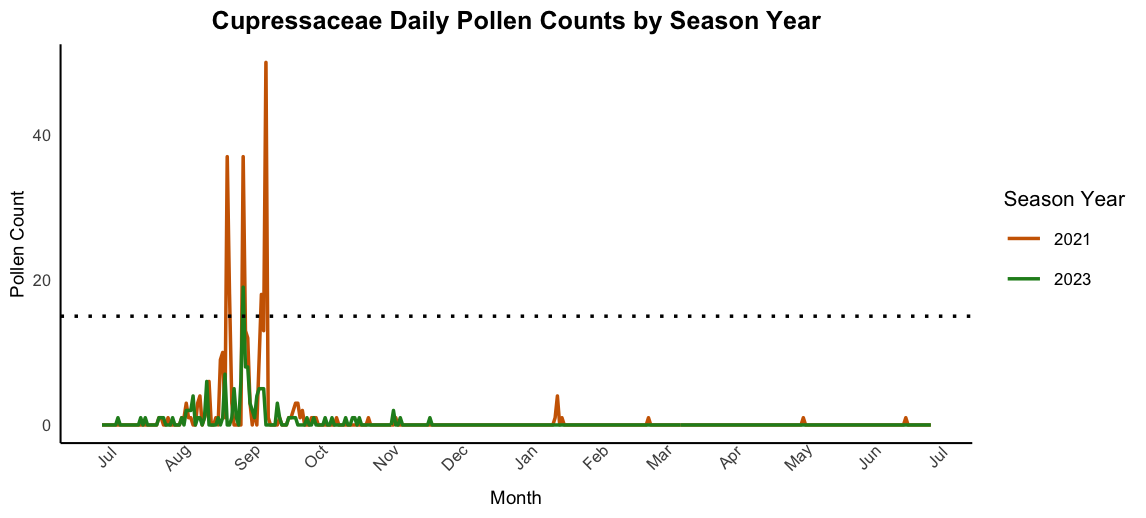


**Fig. S6** The start day number of the pollen season by each group across the five years.


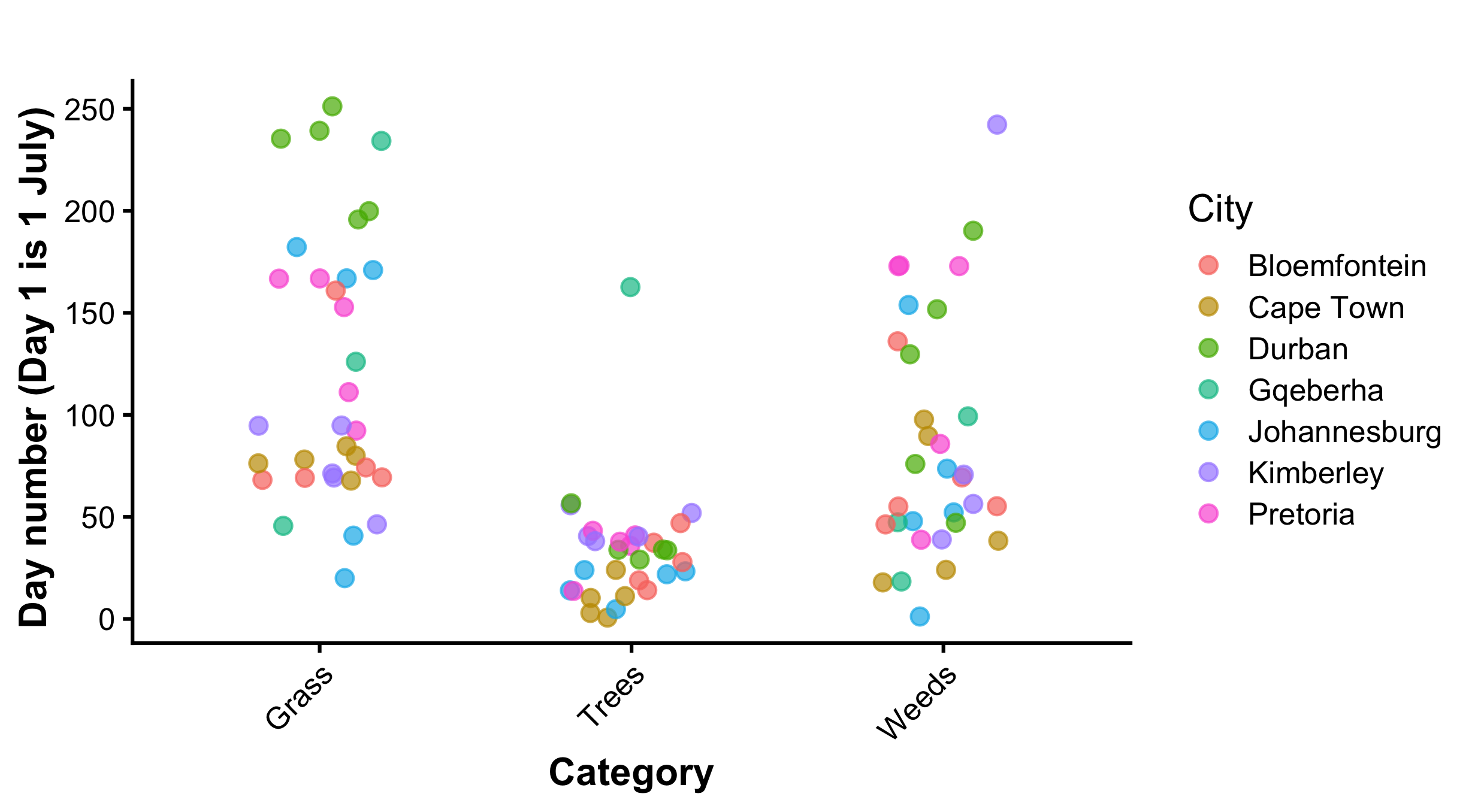


**Fig. S7** A heatmap showing climate and weather seasons of cities in South Africa


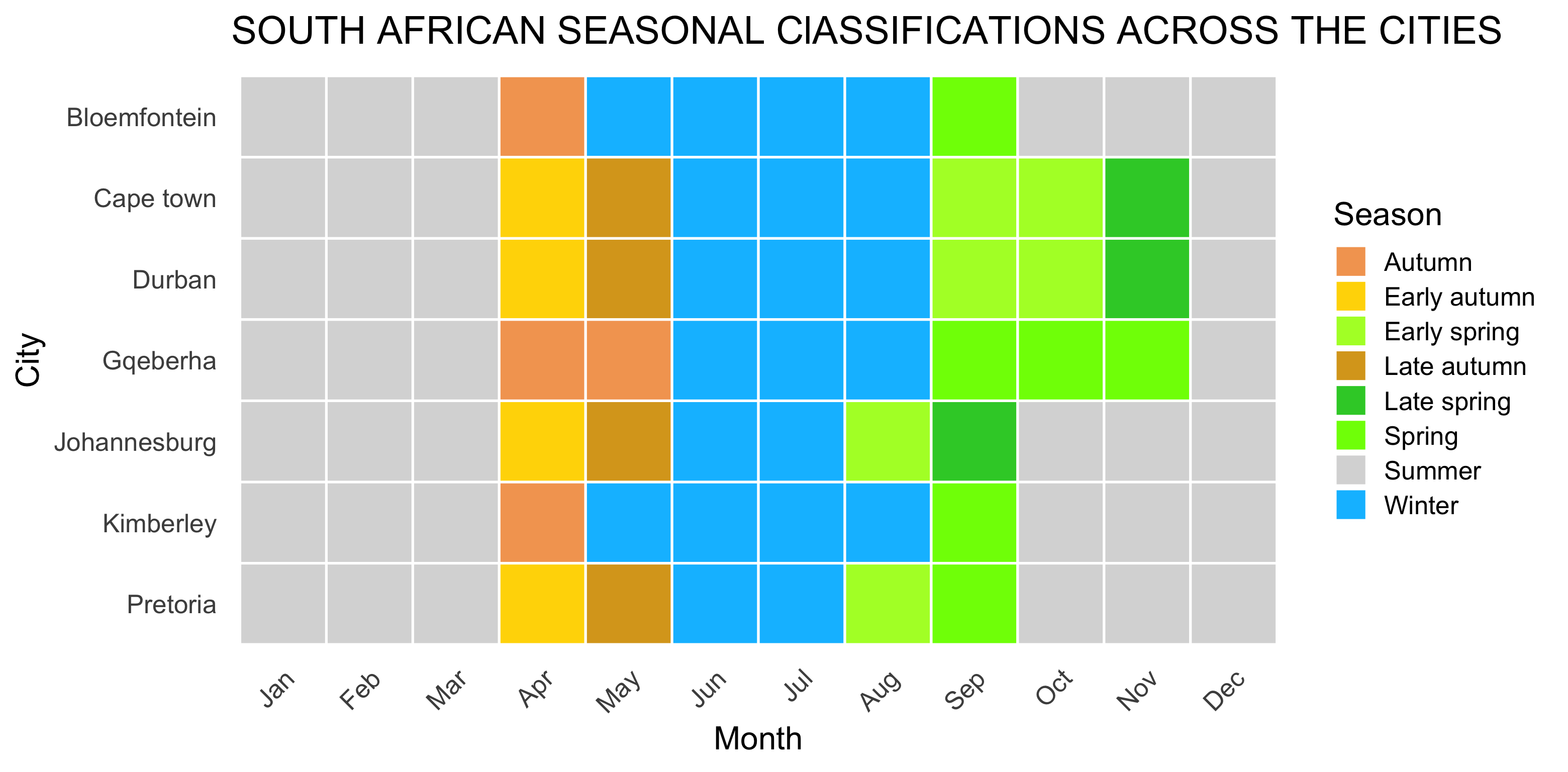


**Fig. S8** A heatmap showing grass, tree and weed pollen seasons and peak months across the cities in South Africa


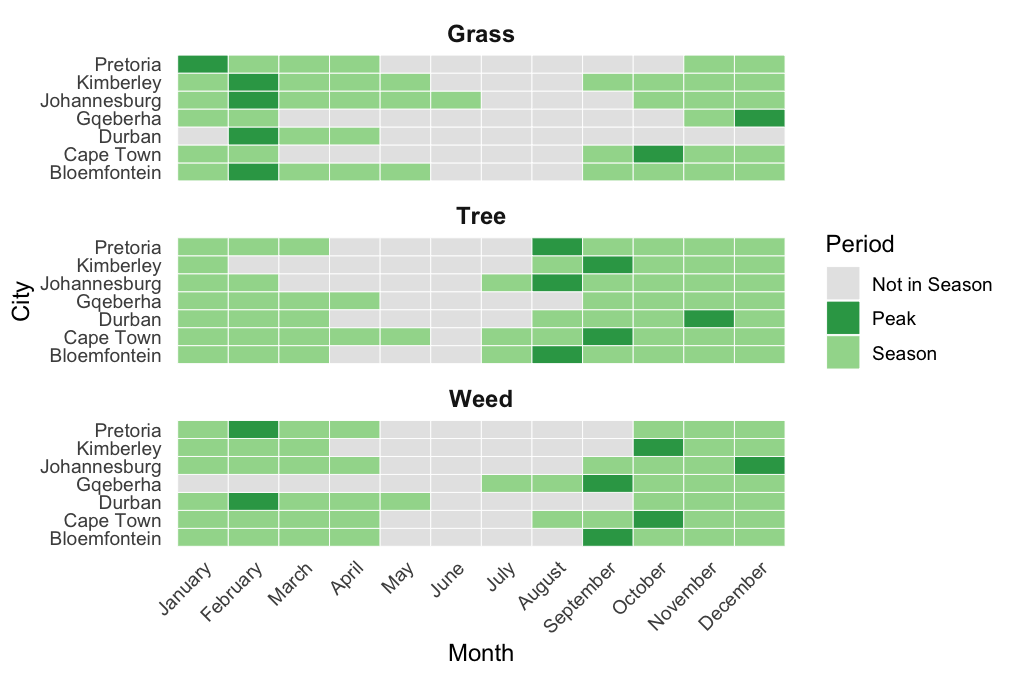


**SUPPLEMENTARY TABLES**

**Table S1** The geography and climate of the biomes in South Africa

| **Biome** | **City** | **Köppen**  **Climate /**  **Humidity**  **Range (%)** | **Rainfall Pattern /**  **Annual Average Rainfall** | **Average  Temperatures**  **(Min-Max)** | **Elevation** | **Population** | **GPS Coordinates** | **Dominant**  **Wind Pattern** |
| --- | --- | --- | --- | --- | --- | --- | --- | --- |
| Fynbos | Cape Town | Mediterranean  60-85% | Winter  515 mm | 11.0 °C-21.5 °C | 0 - 1,590 m | 4.8 million | 33°55′31″S  18°25′26″E | Strong South-easterly |
| Grassland | Johannesburg | Subtropical  Highland  40-75% | Summer  713 mm | 9.5 °C-21.9 °C | 1753 m | 8 million | 26°12′16″S  28°2′44″E | Mostly northern winds, with some western or southern winds |
| Savanna | Pretoria | Subtropical  45-80% | Summer  673 mm | 12.0 °C-25.0 °C | 1400 m | 2.9 million | 25°44′46″S  28°11′17″E | Relatively light winds |
| Grassland | Bloemfontein | Semi-arid  30-65% | Summer  559 mm | 7.0 °C- 24.4 °C | 1395 m | 0.7 million | 29°07′S  26°13′E | South-west winds, with occasional northerly/easterly winds |
| Savanna | Kimberley | Semi-arid  25%-60% | Summer  414 mm | 10.9 °C- 26.1 °C | 1184 m | 0.2 million | 28°44′18″S  24°45′50″E | Variable directions |
| Indian Ocean  Coastal Belt | Durban | Humid-  Subtropical  70-95% | Summer  1019 mm | 16.5 °C-25.2 °C | 8 m | 3.6 million | 29°53′S  31°03′E | North-west winds |
| Albany  Thicket | Gqeberha | Oceanic  65-85% | Year-round  624 mm | 13.5 °C-22.3 °C | 60 m | 1.15 million | 33°57′29″S  25°36′00″E | **South-westerly** in winter, and **north-easterly** in summer |

**Table S2** The average percentage contribution of pollen taxa to the APIn

|  |  |  |  |  |  |  |  |  |
| --- | --- | --- | --- | --- | --- | --- | --- | --- |
|  |  |  |  |  |  |  |  |  |
|  | **CAPE TOWN** | **DURBAN** | **GQEBERHA** | **JOHANNESBURG** | **PRETORIA** | **BLOEMFONTEIN** | **KIMBERLEY** | **All CITIES** |
| Classification | Percentage | Percentage | Percentage | Percentage | Percentage | Percentage | Percentage | Average Percentage |
| *Acacia* | 0.04 | 0.36 | 0.99 | 0.14 | 0.24 | 0.56 | 0.08 | 0.34 |
| *Acer* | 0.02 | 0.03 | 0.09 | 0.01 | 0 | 0 | 0.05 | 0.03 |
| Aizoaceae | 0.06 | 0.28 | 0.04 | 0 | 0 | 0.08 | 0.08 | 0.08 |
| *Amaranthus* | 0 | 0 | 2.64 | 0.46 | 0.18 | 0.02 | 0 | 0.47 |
| Amaryllidaceae | 0 | 0 | 0.44 | 0.05 | 0.01 | 0 | 0.01 | 0.07 |
| *Ambrosia* | 0 | 2.79 | 0 | 0 | 0.08 | 0 | 0.18 | 0.43 |
| Anacardiaceae | 1.13 | 1.28 | 1.69 | 0.04 | 0.06 | 0.55 | 1.86 | 0.94 |
| *Anthospermum* | 0 | 0 | 1.40 | 0 | 0.06 | 0 | 0 | 0.21 |
| Apiaceae | 0.05 | 0.06 | 0.01 | 0.01 | 0 | 0 | 0 | 0.02 |
| Araucariaceae | 0.07 | 0.16 | 0.10 | 0 | 0 | 0 | 0.05 | 0.05 |
| Arecaceae | 0.14 | 0.24 | 0.10 | 0.11 | 0.03 | 0.01 | 0 | 0.09 |
| *Artemisia* | 0.25 | 1.05 | 1.27 | 0.16 | 0.46 | 0.38 | 0.25 | 0.55 |
| Asphodelaceae | 0.03 | 0.01 | 0.35 | 0 | 0.01 | 0.02 | 0.14 | 0.08 |
| Asteraceae | 1.25 | 4.02 | 4.26 | 2.41 | 2.03 | 2.37 | 1.74 | 2.58 |
| *Betul*a | 0.33 | 4.85 | 0.21 | 4.13 | 8.05 | 1.44 | 2.7 | 3.11 |
| Boraginaceae | 0.05 | 0.19 | 0 | 0 | 0.01 | 0.01 | 0 | 0.04 |
| Brassicaceae | 0.02 | 0.10 | 0.01 | 0 | 0.01 | 0 | 0.06 | 0.03 |
| *Buddleja* | 0 | 0.01 | 0 | 0.02 | 0.34 | 8.28 | 0.19 | 1.26 |
| *Cannabis* | 0 | 0.01 | 0 | 0.46 | 0 | 0 | 0 | 0.07 |
| *Carya* | 0.01 | 0.68 | 0.23 | 0.03 | 0.05 | 0.02 | 0.58 | 0.23 |
| Caryophyllaceae | 0.15 | 0.19 | 1.76 | 1.45 | 0.46 | 0.02 | 0.27 | 0.62 |
| *Casuarina* | 0.21 | 0.71 | 4.31 | 0.52 | 0.10 | 0.16 | 0.35 | 0.91 |
| *Cedrus* | 0.38 | 0.12 | 0.90 | 0 | 0.01 | 0 | 0.02 | 0.21 |
| *Celtis* | 0.04 | 0.87 | 0.01 | 2.70 | 0.87 | 6.52 | 2.06 | 1.87 |
| Chenopodiaceae | 0.98 | 1.17 | 1.06 | 0.98 | 0.86 | 1.01 | 1.74 | 1.11 |
| *Citrus* | 0 | 0 | 0 | 0 | 0.01 | 0 | 0 | 0 |
| Combretaceae | 0.05 | 0.32 | 0.03 | 0.64 | 1.61 | 0.72 | 1.19 | 0.65 |
| Crassulaceae | 0.01 | 0.01 | 0 | 0 | 0 | 0.32 | 0 | 0.05 |
| Cupressaceae | 30.23 | 4.27 | 0.79 | 7.55 | 3.48 | 4.47 | 3.816 | 7.80 |
| Cyperaceae | 1.37 | 2.91 | 9.26 | 1.62 | 3.39 | 2.74 | 0.493 | 3.11 |
| *Delonix* | 0.04 | 0 | 0 | 0 | 0 | 0.01 | 00 | 0.01 |
| Dodonaea | 0 | 0 | 0.63 | 0.01 | 0.27 | 0.01 | 0 | 0.13 |
| Ebenaceae | 0.11 | 0.03 | 0.03 | 0 | 0 | 0 | 0 | 0.02 |
| Ericaceae | 0.88 | 0.25 | 3.02 | 0.51 | 0.33 | 0.16 | 0.09 | 0.75 |
| *Erodium* | 0.01 | 0.01 | 0.01 | 0 | 0 | 0 | 0 | 0.01 |
| *Erythrina* | 0 | 0 | 0 | 0.02 | 0.12 | 0.01 | 0 | 0.02 |
| Euclea | 0.01 | 0 | 0.03 | 0 | 0.07 | 0.02 | 0.05 | 0.03 |
| Euphorbiaceae | 0.14 | 0.27 | 1.40 | 0.40 | 0.36 | 0.01 | 0.33 | 0.41 |
| Fabaceae | 0.03 | 0.08 | 0.09 | 0.02 | 0.40 | 0.02 | 0.06 | 0.10 |
| *Fagus* | 0.15 | 0.28 | 0 | 0.10 | 0.62 | 0.13 | 0.34 | 0.23 |
| *Fraxinus* | 0.09 | 0.51 | 0 | 2.35 | 1.98 | 5.04 | 0.92 | 1.56 |
| Gentianaceae | 0 | 0 | 0 | 0.01 | 0.01 | 0 | 0 | 0 |
| Geraniaceae | 0 | 0.11 | 0.03 | 0 | 0 | 0 | 0.01 | 0.02 |
| *Helianthus* | 0 | 0 | 0 | 0 | 0.01 | 0 | 0 | 0 |
| Hippocastinacea | 0.01 | 0.15 | 0.01 | 0 | 0.04 | 0.09 | 0.03 | 0.05 |
| *Ilex* | 0 | 0 | 0 | 0.01 | 0.14 | 0 | 0 | 0.02 |
| Iridaceae | 0.02 | 0.02 | 0.10 | 0 | 0.01 | 0.01 | 0.04 | 0.03 |
| *Jacaranda* | 0.02 | 0 | 0 | 0 | 0.34 | 0.02 | 0 | 0.05 |
| Juglandaceae | 0.03 | 0.07 | 0 | 0.01 | 0 | 0.05 | 0 | 0.02 |
| *Juncus* | 0.12 | 0.12 | 0.01 | 0 | 0.01 | 0.02 | 0.02 | 0.04 |
| Lamiaceae | 0 | 0 | 0 | 0.01 | 0.04 | 0.04 | 0 | 0.01 |
| Liliaceae | 0.01 | 0.04 | 0.93 | 0.03 | 0.01 | 0 | 0.01 | 0.15 |
| *Liquidambar* | 0.01 | 0.02 | 0 | 0.08 | 0 | 0 | 0.06 | 0.02 |
| Loranthaceae | 0 | 0.03 | 0 | 0 | 0.01 | 0 | 0 | 0 |
| Malvaceae | 0.03 | 0.20 | 0.01 | 0.03 | 0.02 | 0.09 | 0.08 | 0.07 |
| *Melia* | 0 | 0 | 0 | 0 | 0 | 0.03 | 0 | 0 |
| Moraceae | 1.45 | 13.17 | 0.19 | 7.82 | 25.29 | 8.19 | 2.9 | 8.43 |
| Myricaceae | 0.78 | 1.88 | 5.48 | 0.01 | 0.05 | 1.17 | 0.22 | 1.27 |
| Myrtaceae | 10.92 | 5.41 | 2.80 | 2.58 | 2.96 | 0.99 | 0.74 | 3.77 |
| *Nuxia* sp | 0.96 | 0.32 | 0 | 0 | 0 | 0 | 0.08 | 0.12 |
| Oenotheracea | 0 | 0.41 | 0.07 | 0 | 0 | 0 | 0 | 0.07 |
| Oleaceae | 2.57 | 1.41 | 3.56 | 1.73 | 1.90 | 5.93 | 3.81 | 2.99 |
| Oxalidaceae | 0 | 0 | 0.23 | 0 | 0 | 0 | 0.09 | 0.05 |
| *Parietaria* | 0.04 | 0.52 | 0 | 0.09 | 0.12 | 0 | 0.19 | 0.14 |
| *Persicaria* | 0.07 | 0.39 | 0 | 0.15 | 0 | 0 | 0.29 | 0.13 |
| Picea | 0.01 | 0 | 0 | 0.01 | 0 | 0 | 0.01 | 0 |
| Pinaceae | 0.02 | 0 | 0 | 0 | 0 | 0 | 0 | 0.02 |
| *Pinus* | 9.46 | 3.38 | 5.04 | 2.08 | 1.83 | 3.25 | 1.09 | 3.73 |
| Plantaginaceae | 1.20 | 2.23 | 0.60 | 0.33 | 0.32 | 1.22 | 0.3 | 0.89 |
| *Platanus* | 4.06 | 0.79 | 0.20 | 31.48 | 12.34 | 9.38 | 1.27 | 8.50 |
| Poaceae | 17.26 | 21.88 | 28.08 | 14.79 | 21.02 | 26.13 | 62.4 | 27.37 |
| *Podocarpus* | 0.21 | 0.91 | 0.47 | 1.44 | 0.36 | 0.39 | 0.21 | 0.57 |
| Polygonaceae | 0.24 | 0.52 | 0.26 | 0.04 | 0.01 | 0.10 | 0.56 | 0.25 |
| *Populus* | 0.14 | 0.41 | 0.06 | 0.63 | 0.50 | 3.77 | 0.39 | 0.84 |
| *Prosopis* | 0.02 | 0.14 | 0 | 0.01 | 1.18 | 0.11 | 0.35 | 0.26 |
| Proteaceae | 0.18 | 0.38 | 1.27 | 0.01 | 0.02 | 0.02 | 0.08 | 0.28 |
| *Quercus* | 1.85 | 0.55 | 0 | 5.17 | 1.75 | 0.41 | 0.2 | 1.42 |
| Ranunculaceae | 0.12 | 0.07 | 0.03 | 0 | 0 | 0 | 0.05 | 0.04 |
| Restionaceae | 0.62 | 0.73 | 1.44 | 0 | 0.01 | 0 | 0.05 | 0.41 |
| Rhamnaceae | 0.03 | 0.01 | 0.12 | 0.01 | 0.01 | 0.02 | 0.01 | 0.03 |
| *Rhus* / *Searsia* | 0.85 | 0.40 | 1.17 | 0.73 | 0.51 | 1.60 | 0.91 | 0.84 |
| Rosaceae | 0.05 | 0 | 0 | 0 | 0 | 0 | 0 | 0.01 |
| *Rumex* | 0.76 | 0.59 | 0.04 | 0.02 | 0.08 | 0.05 | 0.53 | 0.30 |
| Rutaceae | 0.09 | 0.03 | 0.04 | 0 | 0 | 0 | 0.01 | 0.02 |
| *Salix* | 0.03 | 0.03 | 0 | 0.27 | 0.32 | 0.03 | 0.1 | 0.11 |
| Sapotaceae | 0 | 0.03 | 0 | 0 | 0.01 | 0 | 0 | 0.01 |
| *Saxifraga* | 0 | 0.03 | 2.01 | 0.01 | 0 | 0 | 0.06 | 0.30 |
| *Schinus* | 0.67 | 0 | 0 | 0 | 0 | 0 | 0 | 0.67 |
| Sclerocarya | 0 | 0 | 0.25 | 0 | 0.03 | 0 | 0 | 0.04 |
| Scrophulariaceae | 0.03 | 0.03 | 0 | 0 | 0 | 0.01 | 0.01 | 0.01 |
| Solanaceae | 0.01 | 0.02 | 0 | 0.03 | 0 | 0 | 0 | 0.01 |
| *Stoebe* | 0.25 | 0.40 | 2.38 | 0.78 | 1.09 | 0.05 | 0.16 | 0.94 |
| *Taraxacum* | 0.03 | 0.01 | 0.12 | 0 | 0.02 | 0.01 | 0.01 | 0.03 |
| *Theylepteris* | 0 | 0.01 | 0 | 0.01 | 0 | 0 | 0 | 0 |
| Thymelaceae | 0.17 | 0.25 | 2.39 | 0.09 | 0.04 | 0 | 0.13 | 0.52 |
| Tiliaceae | 0.10 | 0.09 | 0.26 | 0.13 | 0.02 | 0.03 | 0.02 | 0.09 |
| Typhaceae | 1.38 | 0.22 | 2.83 | 0.22 | 0.18 | 0.02 | 0.18 | 0.72 |
| *Ulmus* | 0.86 | 1.55 | 0.01 | 1.50 | 0.04 | 1.03 | 0.34 | 1.12 |
| Umbelliferae | 0 | 0.05 | 0 | 0.01 | 0.01 | 0 | 0 | 0.01 |
| Urticaceae | 2.43 | 5.08 | 0.12 | 0.05 | 0.23 | 0.51 | 1.84 | 1.46 |
| *Zea mays* | 0.04 | 0.18 | 0.03 | 0.13 | 0.15 | 0.02 | 0.21 | 0.11 |
| Zygophyllaceae | 0.01 | 0 | 0.06 | 0 | 0 | 0.05 | 0.01 | 0.02 |

**Table S3** Summary of the number of pollen taxa that were recorded at each city in South Africa

| **Site** | **Total** | **Grass** | **Trees** | **Weeds** |
| --- | --- | --- | --- | --- |
| Albany Thicket (Gqeberha) | 70 | 2 | 31 | 37 |
| Grassland (Bloemfontein) | 78 | 2 | 41 | 35 |
| Savanna (Pretoria) | 84 | 2 | 44 | 38 |
| Fynbos (Cape Town) | 83 | 2 | 41 | 40 |
| Indian Ocean  Coastal Belt (Durban) | 82 | 2 | 37 | 43 |
| Grassland (Johannesburg) | 82 | 2 | 40 | 40 |
| Savanna (Kimberley) | 78 | 2 | 38 | 38 |
| Total | 103 | 2 | 50 | 51 |

**Table S4** Summary of pollen taxa that contributed at least 3% of the APIn across the seven biomes in South Africa

|  | **Biome/ City** | **2019** | **2020** | **2021** | **2022** | **2023** |
| --- | --- | --- | --- | --- | --- | --- |
| **COASTAL** | Fynbos (Cape Town) | Cupressaceae, *Pinus*, Poaceae, Myrtaceae, *Platanus*, *Olea* | Poaceae, Cupressaceae, *Pinus*, Myrtaceae, *Platanus*, Restionaceae | Cupressaceae, Myrtaceae  Poaceae, *Pinus*, *Platanus* | Cupressaceae, *Pinus*  Poaceae, Myrtaceae  Urticaceae, *Platanus* | Cupressaceae, Poaceae  Myrtaceae, *Pinus*  Plantaginaceae, *Morus* |
|  | Indian Ocean Coastal Belt (Durban) | Poaceae, *Betula,* *Morus*, Myrtaceae, Asteraceae, *Pinus*, *Ambrosia*, Cyperaceae | Poaceae, *Morus*  Asteraceae, Myrtaceae, *Betula*, Cupressaceae, *Pinus* | Poaceae, *Morus*  Urticaceae, Myrtaceae, *Pinus*, Cupressaceae, Plantaginaceae, Asteraceae, Cyperaceae, | *Morus*, Poaceae, Urticaceae, Ulmaceae, Myrtaceae, Cupressaceae, Myricaceae, *Pinus* | Poaceae, *Morus*  *Betula*, *Ambrosia*  Myrtaceae  Cupressaceae, *Pinus*, Urticaceae |
|  | The Albany thicket (Gqeberha) | Poaceae, *Pinus*, Cyperaceae, Asteraceae  *Casuarina*, Proteaceae, Myrtaceae | Poaceae, Cyperaceae  *Olea*, Myricaceae, Asteraceae, *Casuarina*, Ericaceae, Caryophyllace, Anacardiacee, *Pinus* | Poaceae, *Casuarina*  Cyperaceae, *Olea,* *Amaranthus*, *Pinus*, *Stoebe*, Anacardiacee, Myricaceae, Asteraceae, Ericaceae | Poaceae, Myricaceae, Thymelaceae, Cyperaceae  *Amaranthus*, Myrtaceae *Pinus*, Asteraceae, *Rhus* / *Searsia*, *Anthospermm* | Poaceae, Typhaceae  Cyperaceae, *Saxifraga*  *Pinus*, Myricaceae, Asteraceae, Restionaceae, Ericaceae |
| **INLAND** | Grassland  (Johannesburg) | *Platanus*, Poaceae, Cupressaceae, *Morus*, *Betula,* *Quercus*, Asteraceae | Platanus, Poaceae, *Morus*, Cupressaceae, *Quercus*, *Betula*, Asteraceae | *Platanus*, Poaceae *Morus*, Cupressaceae, *Betula*, *Fraxinus,* *Quercus* | *Platanus,* Poaceae, Cupressaceae, *Quercus*, *Celtis*, *Morus*, Ulmaceae, Myrtaceae, *Olea* | *Platanus*, Poaceae, Cupressaceae, *Morus*, *Celtis*, *Quercus*, *Fraxinus* |
|  | Savanna  (Pretoria) | Poaceae, *Morus*  *Platanus*, *Betula*, Cupressaceae, Myrtaceae | Poaceae, *Morus,*  Cupressaceae, Stoebe-type | Poaceae, *Morus*  Cyperaceae, *Platanus*, *Betula*, *Prosopis*, Asteraceae, Myrtaceae | *Morus*, *Platanus*  Poaceae, *Betula*, *Fraxinus*, Cupressaceae | *Morus*, Poaceae  *Platanus*, *Betula,* Cupressaceae |
|  | Grassland (Bloemfontein) | Poaceae, Oleaceae  *Buddleja*, Cupressaceae  *Morus*, *Fraxinus* | Poaceae, *Buddleja Fraxinus*, *Platanus*, Cupressaceae, *Pinus*, Asteraceae | Poaceae, *Morus*, *Celtis*  *Platanus*, Myricaceae, *Searsia* (*Rhus*)*,* Asteraceae, *Olea* | *Celtis*, Poaceae, Cupressaceae, *Platanus*, Moraceae, *Populus*, *Olea,* *Buddleja* | *Platanus*, *Morus*, Poaceae, *Populus*, *Olea*, Cyperaceae, *Pinus*, *Betula* |
|  | Savanna Kimberley | Poaceae | Poaceae, *Olea*  Cupressaceae | Poaceae, Urticaceae, Cupressaceae, *Celtis*  *Olea*, Anacardiaceae | Poaceae, *Betula*, Cupressaceae, *Morus*, *Platanus*, Anacardiaceae | Poaceae, Oleaceae  *Celtis*, Cupressaceae, *Morus* |

**Table S5 Summary of pollen season characteristics from 2019 to 2024 across biomes**

| **Biome (City)** | | **Pollen Type** | **Season Start** | **Season End** | **Duration in days** | **Peak Month**  **(Max week and year)** |
| --- | --- | --- | --- | --- | --- | --- |
|  |  |  | **Mean (range)** | **Mean (range)** | **Mean (range)** |  |
| **COASTAL** | **Fynbos (Cape Town)** | Grass | Sep (6-23 Sep) | Feb (Nov-May) | 144 (81-243) | Oct (26 Oct-1 Nov 2020) |
|  |  | Tree | Jul | May (Jan-Jun) | 297 (187-351) | Sep (13-19 Sep 2021) |
|  |  | Weed | Aug (Jul-Oct) | Apr (Mar-Jun) | 232 (173-269) | Oct (4-10 Oct 2021) |
|  | **Indian Ocean Coastal Belt (Durban)** | Grass | Feb (Jan-Mar) | Apr (Feb-May) | 71 (20-104) | Feb (17-23 Feb 2020) |
|  |  | Tree | Aug (Jul-Aug) | Mar (Feb-Mar) | 212 (186-225) | Nov (27 Feb-5 Mar 2023) |
|  |  | Weed | Oct (Aug-Jan) | May (Mar-Apr) | 186 (142-224) | Feb (5-11 Apr 2021) |
|  | **Albany Thicket (Gqeberha)** | Grass | Nov (Aug-Feb) | Feb (Oct-Apr) | 92 (42-157) | Dec (1-7 Apr 2024) |
|  |  | Tree | Sep (Aug-Dec) | April only | 116 | N/A |
|  |  | Weed | Jul/Aug | Dec (Oct-Mar) | 105 (16-234) | Sep (18-24 Sep 2023) |
| **INLAND** | **Grassland (Johannesburg)** | Grass | Oct (Jul-Dec) | Jun (May-Jun) | 222 (157-298) | Feb (24 Feb-1 Mar 2020) |
|  |  | Tree | Jul | Feb (Dec-Jun) | 224 (157-279) | Aug (31 Aug-6 Sep 2020) |
|  |  | Weed | Sep (Jul-Dec) | Apr (Jan-Jun) | 233 (148-315) | Dec (5-11 Apr 2021) |
|  | **Savanna (Pretoria)** | Grass | Nov (Sep-Dec) | Apr (Mar-May) | 150 (98-194) | Jan (24-30 Jan 2022) |
|  |  | Tree | Aug (Jul-Aug) | Mar (Dec-Jun) | 237 (137-317) | Aug (22-28 Aug 2022) |
|  |  | Weed | Oct (Aug-Dec) | Apr/May | 172 (117-250) | Feb (31 Jan-6 Feb 2022) |
|  | **Grassland (Bloemfontein)** | Grass | Sep | May (Apr-May) | 246 (223-278) | Feb (3-9 Feb 2020) |
|  |  | Tree | Jul-Aug | Mar (Jan-Jun) | 221 (182-309) | Aug (7-13 Sep 2020) |
|  |  | Weed | Sep (Aug-Nov) | Apr (Jan-Jun) | 217 (151-256) | Sep (18-24 Sep 2023) |
|  | **Savanna (Kimberley)** | Grass | Sep (Aug-Oct) | May | 247 (223-276) | Feb (17-23 Jan 2022) |
|  |  | Tree | Aug (9-25 Aug) | Jan (Oct-Jun) | 163 (69-302) | Sep (6-12 Sep 2021) |
|  |  | Weed | Oct (Aug-Feb) | Mar (Oct-Apr) | 155 (55-248) | Oct (23-29 Aug 2021) |
